# Efficacy and acceptability of third-wave psychotherapies in the treatment of depression: a network meta-analysis of controlled trials

**DOI:** 10.3389/fpsyt.2023.1189970

**Published:** 2023-10-05

**Authors:** Cora Schefft, Christian Heinitz, Anne Guhn, Eva-Lotta Brakemeier, Philipp Sterzer, Stephan Köhler

**Affiliations:** ^1^Department of Psychiatry and Neurosciences, Charité—Universitätsmedizin Berlin, Corporate Member of Freie Universität Berlin and Humboldt—Universität zu Berlin, Berlin, Germany; ^2^Department of Clinical Psychology and Psychotherapy, University of Greifswald, Greifswald, Germany

**Keywords:** third-wave psychotherapies, cognitive-behavioral therapy, depression, major depressive disorder, psychotherapy, network meta–analysis

## Abstract

**Introduction:**

In recent decades, various new psychotherapy approaches have been developed in an effort to overcome issues of non-response, referred to as “third-wave psychotherapies.” How third-wave therapies perform in comparison to each other, to classical CBT, or other common comparators in the treatment of depression has not yet been systematically assessed.

**Methods:**

We firstly determined the scope of the term “third-wave” by conducting a systematic search. The identified approaches were then used as search terms for the systematic review and network meta-analysis (NMA). We searched MEDLINE, CENTRAL, PsychINFO and Web of Science from inception until 31 July 2022. We assessed randomized controlled trials comparing third-wave psychotherapies to each other, CBT, treatment as usual (TAU), medication management, active control conditions, or waitlist (WL) in adult populations with depressive disorders. The treatments included were acceptance and commitment therapy, behavioral activation, cognitive behavioral analysis system of psychotherapy, dialectical behavioral therapy, mindfulness-based cognitive therapy, meta-cognitive therapy, positive psychotherapy and schema therapy. The primary outcome was depression severity (efficacy) at study endpoint, and the secondary outcome was all-cause discontinuation (acceptability). This review was registered in PROSPERO, identifier CRD42020147535.

**Results:**

Of 7,971 search results, 55 trials were included in our NMA (5,827 patients). None of the third-wave therapies were more efficacious than CBT but most were superior to TAU [standardized mean differences (SMD) ranging between 0.42 (95% CI −0.37; 1.19) and 1.25 (0.48; 2.04)]. Meta-cognitive therapy (MCT) was more efficacious than three other third-wave therapy approaches. None of the third-wave treatments were more acceptable than WL or CBT. Twenty-seven percent of the trials were rated as low risk of bias. Confidence in the evidence was largely low according to GRADE. Inconsistency emerged for a small number of comparisons.

**Interpretations:**

Third-wave therapies are largely efficacious and acceptable alternatives to CBT when compared to TAU, with few differences between them. The evidence so far does not point toward superiority or inferiority over CBT. Patient-level research may offer possibilities for tailoring individual psychotherapies to the needs of individual patients and future trials should make this data available. The evidence base needs to be broadened by sufficiently powered trials.

## 1. Introduction

Depressive disorders represent a major challenge in public health, with a 12–month prevalence of 5.37%–6.9% in the European Union and an estimated lifetime prevalence of 6%–25% (1). Depression contributes significantly to the worldwide burden of disease and is globally ranked the 6th most common cause of disability–adjusted life years in the age group of 25–49 years (2).

In the treatment of major depressive disorder (MDD), psychotherapy is recommended as a first–line option by several treatment guidelines (3, 4). Different types of psychotherapy have demonstrated efficacy in the treatment of MDD (5), with response rates of 41% (compared with 17% in usual care) and a number needed to treat of 5.3 (6). In terms of the number of randomized controlled trials (RCT), cognitive–behavioral therapy (CBT) ranks first (7, 8) although other psychotherapy approaches, such as psychodynamic, or interpersonal psychotherapies (IPT), have been shown to be effective in the treatment of MDD (9–11) via several meta-analyses (12–14).

In the past decades, various new psychotherapeutic approaches have been developed, aiming to improve current ones (6, 15). These new approaches were jointly referred to as the “third-wave” of behavioral therapies (16). The first wave of behavioral therapy predominantly drew from the principles of operant classical conditioning; however, the second wave added the “C” to behavioral therapy to reflect the addition of cognitive components such as modifying dysfunctional beliefs, following the “cognitive revolution” of the 1970s. The third–wave of CBT encompasses a range of approaches (17). Hayes and Hoffmann (17) summarized four key features of third-wave CBT psychotherapies: a focus on context and function; being built on other strands of CBT; a focus on broad and flexible repertoires vs. signs and symptoms; and integrating humanistic, existentialist, analytical, or system-oriented approaches. Generally speaking, third-wave psychotherapies focus on patients’ relationships with a certain behavior or thought rather than on their respective content. Conceptually, foci on mindfulness, acceptance, relationships and meta-cognitions are added. Interventions most commonly referred to as third-wave psychotherapies are acceptance and commitment therapy (ACT), mindfulness-based cognitive therapy (MBCT), dialectical behavior therapy (DBT), functional-analytical psychotherapy (FAP), and behavioral activation (BA). Less frequently cited are meta-cognitive therapy (MCT), schema therapy (ST), integrative behavioral couples therapy (IBCT), cognitive behavioral analysis system of psychotherapy (CBASP), positive psychotherapy (PP), and compassion-focused therapy (CFT) (18).

Over the last decade, there has been a growing number of RCTs and meta-analyses investigating the efficacy of third-wave psychotherapies (19–22). Previous reviews and meta-analyses, however, did not focus on clinical depression solely but on a variety of disorders and conditions (16, 18), while others that focused on depression, pooled different third-wave psychotherapies into one category for meta-analytical comparison (23). In this article, we focused on patients with clinical depressive disorders and aimed to synthesize data on the efficacy of individual third-wave psychotherapies in this patient group. We firstly identified the scope of the concept of third-wave psychotherapies for the treatment of clinical depression using a systematic literature review. Secondly, based on the results of our preliminary review, we included in our analysis available efficacy and acceptability data of several third-wave psychotherapy approaches. We performed a network meta-analysis (NMA) to compare individual third-wave psychotherapies (a) to CBT; (b) to other common comparators, such as waitlist (WL), treatment as usual (TAU), medication management (MM), and active controls (ActiveCt; e.g., “sham” psychotherapy); and (c) head-to-head.

## 2. Methods

### 2.1. PROSPERO and PRISM

We registered the protocol in PROSPERO (CRD42020147535; Supplementary Text S1). We followed the Preferred Reporting Items for Systematic Reviews and Meta-Analysis in its extended version for NMAs (24).

### 2.2. Selection of third-wave psychotherapies

To firstly define the scope of interventions belonging to the category of third-wave psychotherapies, we combined information from previously published meta-analyses (16, 18) and a systematic database search in MEDLINE and PsycINFO from inception to 31 December 2019. We combined the search terms, “third-wave” and “depression.” The search yielded 137 articles in PsycINFO (books excluded) and 72 in MEDLINE, 24 of which were duplicates.

The literature search identified the following psychotherapies that were mentioned at least once as third-wave psychotherapies: ACT, BA, CBASP, CFT, DBT, emotion-focused therapy (EFT), FAP, IBCT, MCT, MBCT, mindfulness-based stress reduction (MBSR), rumination-focused CBT (RFCBT), PP, ST, and well-being therapy (WBT).

Based on these results of our search, we included all of the mentioned psychotherapies in our main search for the systematic review except for RFCBT because of its strong overlap with CBT and EFT because of its origin in client-centered therapy. We did not include MBSR since it was conceptualized for a broader health-related context, while MBCT is the depression-specific integration of mindfulness practice in a psychotherapy framework (25).

### 2.3. Data sources

For our NMA, we searched several databases (MEDLINE, Cochrane CENTRAL, Web of Science, PsycINFO) from inception to 31 July 2022. We searched for RCTs using the search terms “depress*” or “depression” and the respective forms of treatment (Supplementary Text S2). We also searched trial registries clinicaltirals.gov and the International Clinical Trials Registry Platform (ICTRP) as well as dissertations (via PsycINFO) for any unpublished data. Studies were searched, screened, selected and data was extracted independently by two authors (CS, CH). Inconsistencies were resolved by discussion between the two authors in consultation with the third and the last author.

### 2.4. Study selection

We included trials that compared third-wave psychotherapies:

- with cognitive therapy (CT)/CBT; or
- with a control group, such as
  - WL,
  - TAU (treatment as usual or care as usual commonly refers to a regimen of varying intensity. TAU does not follow a protocol that is structured within the trial. Most often, it includes antidepressant treatment and follow-up visits to a psychiatrist. Treatments comparing against TAU usually include TAU in the intervention group as well, i.e., they compare intervention + TAU vs. TAU),
  - MM (antidepressant medication treatment following a structured protocol or algorithm specified within the trial),
  - ActiveCt conditions (a condition structured within the trial that is roughly equal to treatment intensity in the intervention arm but not applying the active intervention. For example “sham” or “placebo” psychotherapies or psychoeducation only. Comparison against an ActiveCt condition is meant to test whether a treatment’s specific interventions are more efficacious than non-specific factors common to all psychotherapies); or
- head-to-head.

A main rationale in clustering control conditions was to differentiate structured conditions (MM, ActiveCt) from non-structured ones (WL, TAU). The clustering criteria for nodes are in Supplementary Text S3. Further inclusion criteria were: Adult population, English or German language, a primary diagnosis of major depressive disorder (MDD) or episode, persistent depressive disorder (PDD) according to the DSM-IV or -5 criteria or ICD-10 criteria, depression severity was measured by standard measures of depressive symptoms (Supplementary Text S4; Supplementary Table S5); trials delivered inpatient or outpatient treatments face-to-face, in groups, digitally, or via telephone.

Patient populations with comorbid medical conditions or pregnant women were not excluded.

### 2.5. Main outcomes and measures

We defined the differences in depression severity between arms as our continuous primary outcome of efficacy. If data from several outcome measures were reported, we included data according to a prespecified hierarchy, favoring clinician-rated outcomes over self-report (Supplementary Text S4). As a proxy for acceptability, we included all-cause discontinuation as a dichotomous secondary outcome. All-cause discontinuation was defined as the number of patients who withdrew from the study after commencing treatment but before the endpoint. Primary outcome data was obtained at the endpoint of the intervention.

### 2.6. Data extraction and synthesis

For efficacy, standardized mean differences (SMD) were calculated as *Cohen’s d*. The superiority of a treatment was expressed by a positive value. For acceptability, summary odds ratios (ORs) were calculated. An OR > 1 indicated a higher probability of discontinuation for the first than for the second treatment.

Effect estimates were synthesized using Bayesian random effects (RE) NMA assuming a common intertrial variance (heterogeneity, tau^2^). We used the package *gemtc* (26, 27) in RStudio (R version 4.2.1) (28) applying Monte Carlo Markov Chain sampling as implemented in JAGS, and BUGSnet (29) for network graphs. For continuous outcomes, normal likelihoods were assumed; for dichotomous outcomes, binomial likelihoods were assumed. Results are presented as relative effect sizes and ORs, with 95% credible intervals (CrIs). Vague prior distributions were assumed for the baseline effects, the treatment effects relative to the baseline effect (both normally distributed), and the heterogeneity (uniform distribution) (26). Model specification, parametrization of priors, and assessment of convergence are provided in Supplementary Text S6. Treatment rankings are reported as the surface under the cumulative ranking curve (SUCRA) (30). Inconsistency was assessed locally in node–splitting models as automated in *gemtc* (31) and globally by comparing the fit (deviance information criterion, DIC) of an inconsistency model (32). Pairwise meta-analyses were calculated in *meta* (33). Heterogeneity was assessed using the *I^2^* statistic and *p*-values from *Cochran’s Q-test*. We tested for small study effects (publication bias) by calculating Egger’s test for pairwise comparisons with k ≥ 3 (34).

We assessed sources of heterogeneity in pre-specified meta-regression models: (1) high vs. low risk of bias; (2) manualized vs. non-manualized control conditions; and (3) severity of depression at baseline as a covariate. We performed *post-hoc* sensitivity analyses by (1) only including trials that focused on PDD and/or treatment-resistant populations, (2) excluding special/comorbid populations, and (3) excluding trials with WL or MM conditions since they might violate the transitivity assumption.

Risk of bias was assessed for the primary outcome using the Cochrane Collaboration’s risk of bias tool 2, which was adapted for psychological interventions given that participants are not blind to the intervention they receive (Supplementary Text S7) (35, 36). Risk of bias ratings were performed by two authors (CS and CH) and all disparities were solved by discussion. Confidence in effect estimates for efficacy outcomes was rated using the Grading of Recommendations Assessment, Development, and Evaluation (GRADE) framework, implemented in CINeMA (37).

## 3. Results

### 3.1. Characteristics of included studies

**Figure 1:**
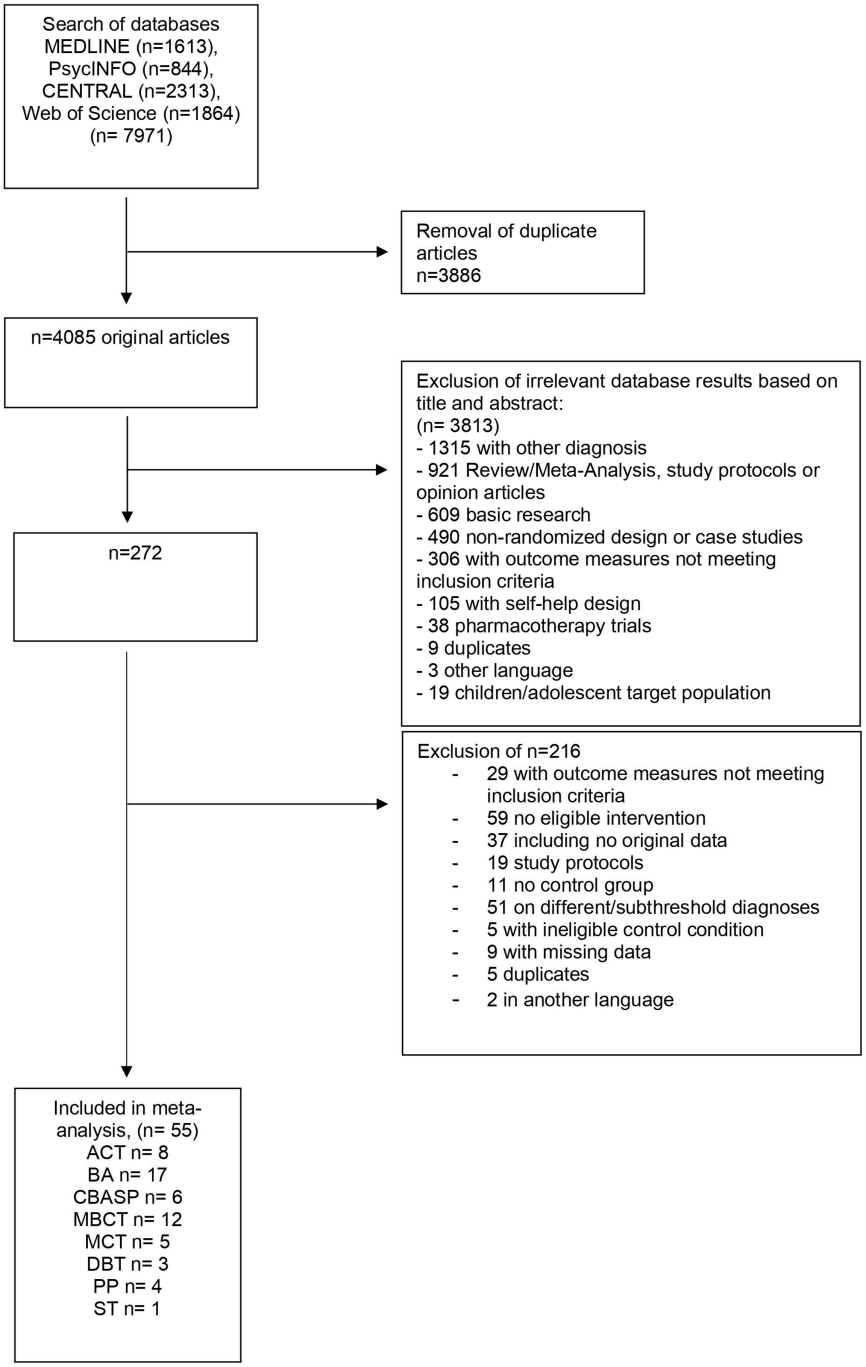
Flow-chart of the study selection process. ACT, acceptance and commitment therapy; BA, behavioral activation therapy; CBASP, cognitive behavioral analysis system of psychotherapy; DBT, dialectical behavioral therapy; MBCT, mindfulness-based cognitive therapy; MCT, meta-cognitive therapy; PP, positive psychotherapy; ST, schema therapy.

The database search yielded 7,971 results. A total of 272 full-texts remained after excluding duplicates and screening titles and abstracts, 55 of which were eligible for inclusion in the NMA (Figure 1). No eligible trials were retrieved for WBT, FAP, or IBCT. The final set of 55 studies comprised 72 direct comparisons (Supplementary Table S8). The comparisons included 5,827 patients for efficacy, and 5,757 for acceptability data. A total of 3,009 patients were randomized to third–wave psychotherapies, and 2,818 patients were randomized to control conditions. The mean age of patients was 39.53 (SD 7.98) years (Supplementary Figure S9), the mean percentage of female participants was 65.5% (SD 18.5). Thirty-three studies investigated psychotherapy in a face-to-face format, 18 included group treatments, and four trials included internet- or telephone-delivered formats. The median duration of trials was 12 weeks (inter-quartile range 8–16). Sixteen trials recruited patients from the Americas, thereof 14 from the United States, 24 from Europe, three from Australia and New Zealand, and 12 from Asia, 10 thereof from Iran (Supplementary Table S10). All trials included patients diagnosed with MDD. In 16 trials, persistent and/or treatment-resistant courses were exhibited by at least 30% (mean 87.5%, SD 24.05) of patients. Four trials included special populations or patients with comorbidities: postpartum women, multiple sclerosis, physical disabilities, and chronic pain. Study characteristics are summarized in detail in Supplementary Table S11.

### 3.2. Network plot

**Figure 2:**
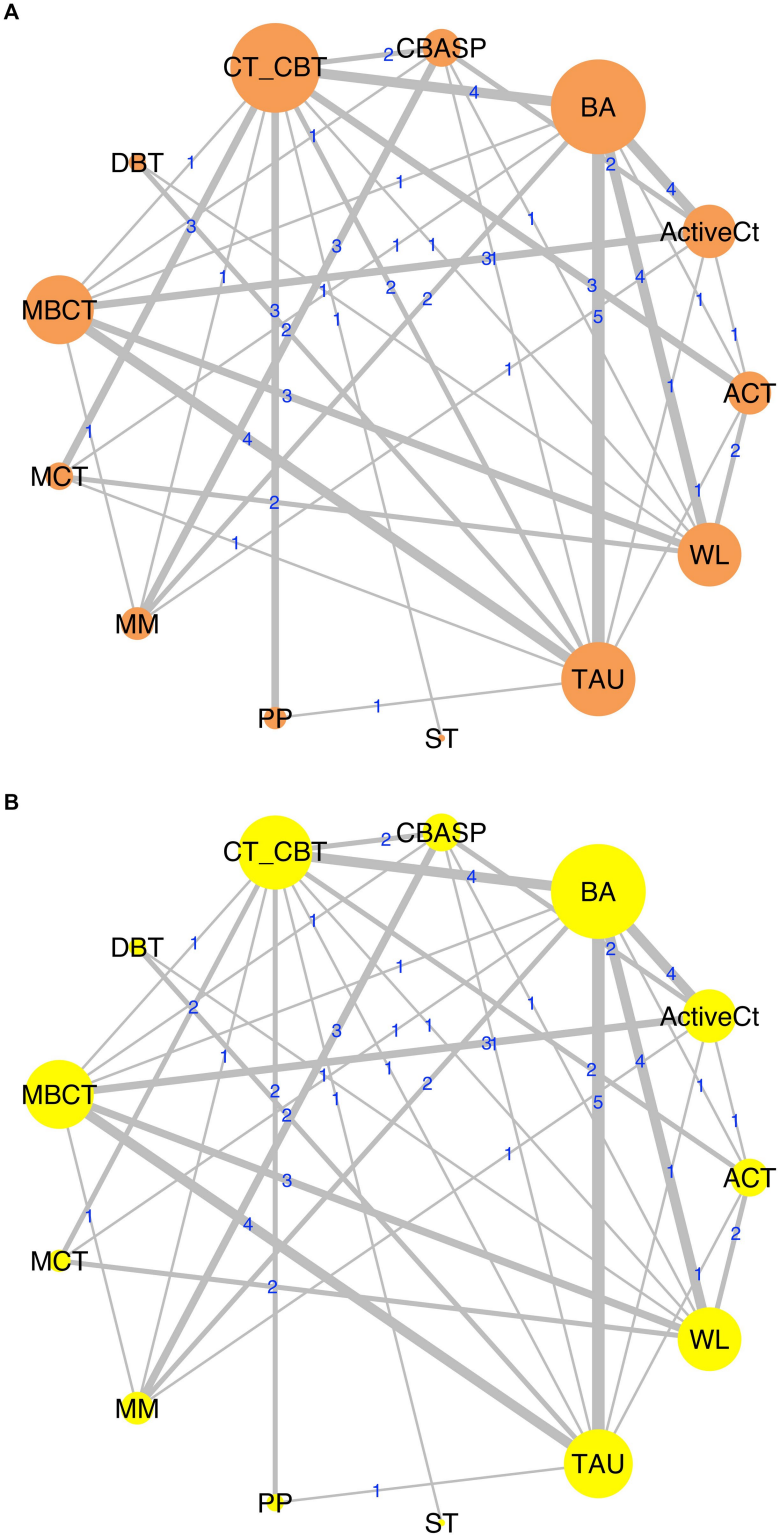
Network graphs of efficacy **(A)** and acceptability **(B)** data. ACT, acceptance and commitment therapy; ActiveCt, active control group; BA, behavioral activation therapy; CBASP, cognitive behavioral analysis system of psychotherapy; CT/CBT, cognitive therapy/cognitive-behavioral therapy; DBT, dialectical behavioral therapy; MBCT, mindfulness-based cognitive therapy; MCT, meta-cognitive therapy; MM, medication management; PP, positive psychotherapy; ST, schema therapy; TAU, treatment as usual; WL, waitlist.

The network geometries for both efficacy and acceptability are displayed in Figures 2A,B. The efficacy network included four head-to-head comparisons between third-wave psychotherapies. Seventeen comparisons compared a third-wave psychotherapy with CT/CBT (*n* = 1,630), 13 with WL (*n* = 903), 17 with TAU (*n* = 938), six with MM (*n* = 1,286), and ten with an ActiveCt (*n* = 1,427; Supplementary Table S12). All third–wave treatments except DBT were directly compared with CT/CBT.

### 3.3. Network meta-analysis

**Figure 3:**
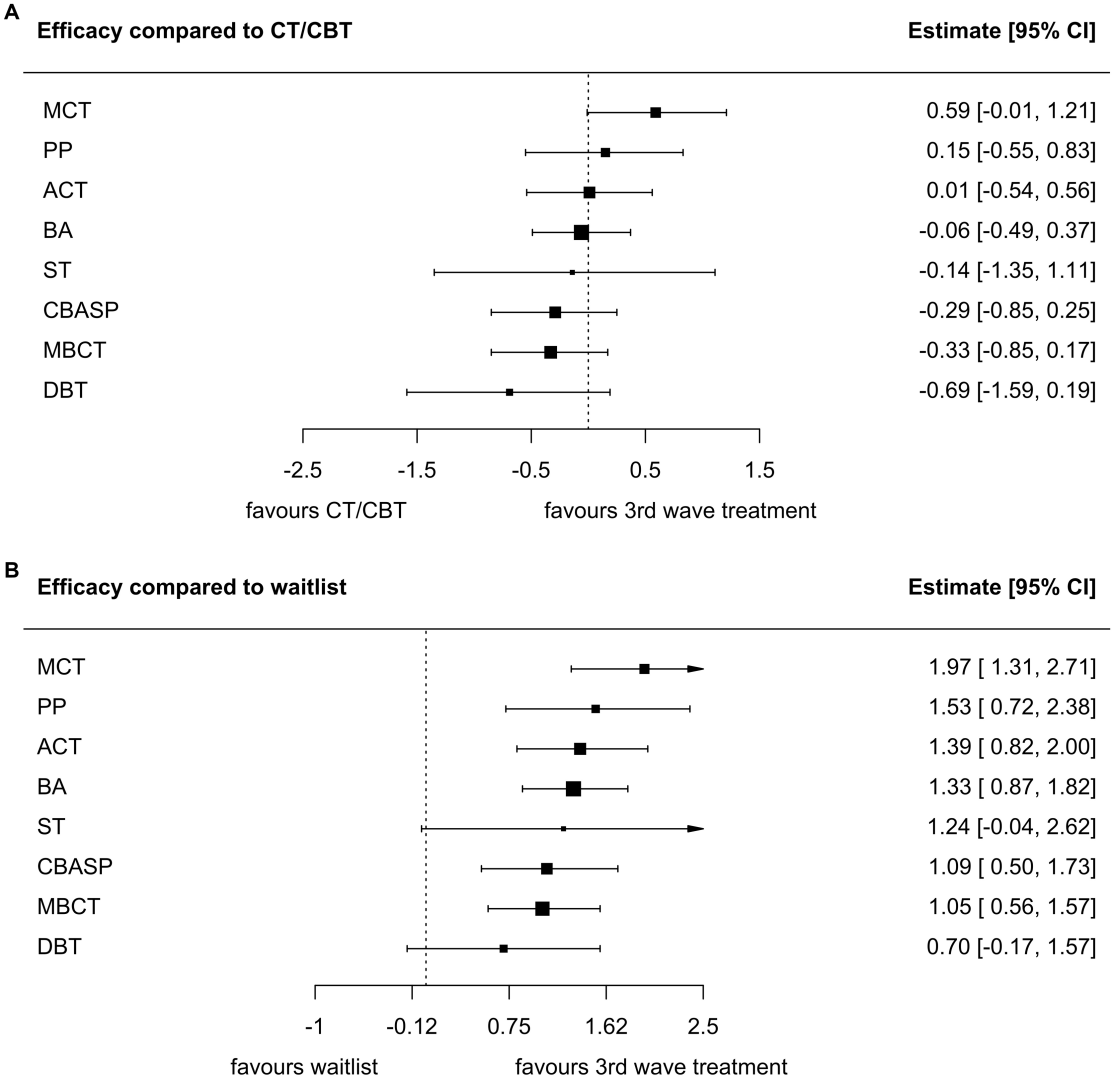
Forest plots of treatment efficacy of third-wave therapies in comparisons with CT/CBT **(A)** and WL **(B)** as treatments of reference. ACT, acceptance and commitment therapy; BA, behavioral activation therapy; CBASP, cognitive behavioral analysis system of psychotherapy; DBT, dialectical behavioral therapy; MBCT, mindfulness-based cognitive therapy; MCT, meta-cognitive therapy; PP, positive psychotherapy; ST, schema therapy.

**Figure 4:**
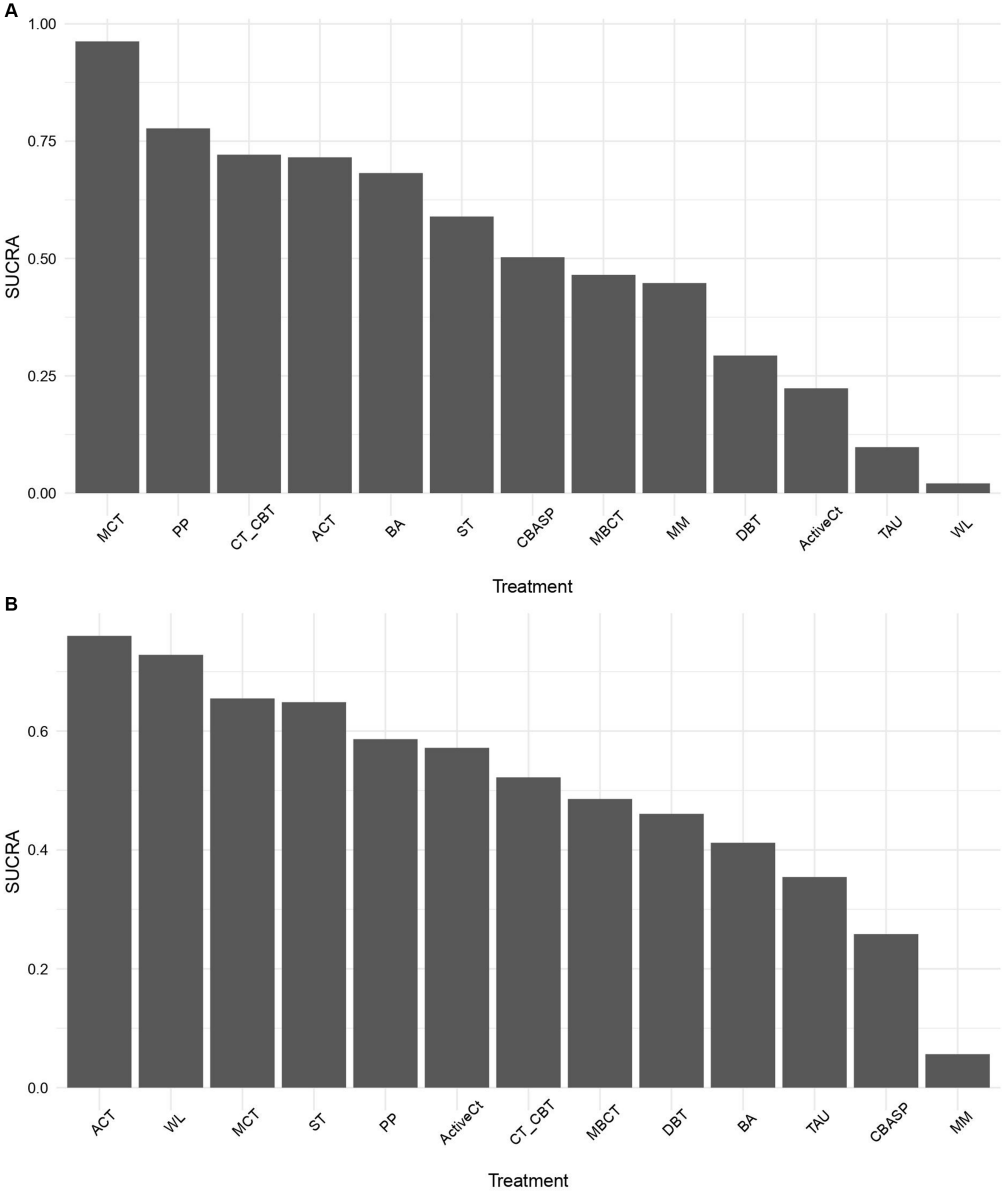
Surface und the cumulative ranking curve (SUCRA) plots of the efficacy **(A)** and acceptability **(B)** of third-wave psychotherapies. ACT, acceptance and commitment therapy; ActiveCt, active control group; BA, behavioral activation therapy; CBASP, cognitive behavioral analysis system of psychotherapy; CT/CBT, cognitive therapy/cognitive-behavioral therapy; DBT, dialectical behavioral therapy; MBCT, mindfulness-based cognitive therapy; MCT, meta-cognitive therapy; MM, medication management; PP, positive psychotherapy; ST, schema therapy; TAU, treatment as usual; WL, waitlist.

None of the third-wave psychotherapies differed from CT/CBT in terms of efficacy. All third–wave psychotherapies except DBT and ST were significantly superior to WL and TAU conditions for lowering depression scores, with effect sizes ranging from 0.78 to 1.99 (Figures 3A,B, TAU in Supplementary Table S13). BA, ACT, PP, and MCT were more efficacious than ActiveCt. Only MCT was more efficacious than MM. In head-to-head comparisons (Table 1), MCT showed superior efficacy to MBCT (SMD 0.92, 95% CrI 0.23–1.65), DBT (SMD 1.27, 95% CrI 0.3–2.33) and CBASP (SMD 0.88, 95% CrI 0.14–1.67). MCT had the highest probability of all treatments to rank first in terms of efficacy, followed by BA. WL ranked last (Figure 4).

**Table 1:** Relative effects for the efficacy and acceptability of third–wave psychotherapies.

ACT	1.45 (0.47, 4.44)	1.77 (0.63, 5.05)	2.27 (0.73, 7.39)	1.52 (0.55, 4.22)	1.7 (0.35, 8.33)	1.62 (0.53, 4.95)	1.17 (0.26, 5.16)	**3.71 (1.14, 12.55)**	1.35 (0.31, 5.7)	1.11 (0.16, 7.77)	1.92 (0.66, 5.75)	1.12 (0.37, 3.49)
**0.78 (0.15, 1.43)**	ActiveCt	1.22 (0.58, 2.61)	1.57 (0.72, 3.53)	1.06 (0.44, 2.46)	1.19 (0.27, 5.16)	1.12 (0.53, 2.36)	0.82 (0.2, 3.19)	**2.59 (1.09, 6.23)**	0.93 (0.24, 3.46)	0.77 (0.11, 4.95)	1.34 (0.54, 3.22)	0.78 (0.3, 1.97)
0.06 (−0.51, 0.64)	**−0.71 (−1.19, −0.26)**	BA	1.28 (0.58, 2.94)	0.87 (0.43, 1.72)	0.96 (0.24, 3.86)	0.91 (0.44, 1.9)	0.66 (0.18, 2.39)	2.1 (0.93, 4.85)	0.76 (0.22, 2.56)	0.63 (0.1, 3.78)	1.09 (0.53, 2.2)	0.63 (0.28, 1.4)
0.3 (−0.38, 0.99)	−0.48 (−1.04, 0.07)	0.23 (−0.3, 0.79)	CBASP	0.68 (0.3, 1.48)	0.75 (0.17, 3.29)	0.71 (0.3, 1.63)	0.52 (0.12, 1.93)	1.65 (0.76, 3.53)	0.59 (0.16, 2.12)	0.49 (0.07, 3.03)	0.85 (0.33, 2.08)	0.49 (0.19, 1.27)
0.01 (−0.53, 0.55)	**−0.77 (−1.32, −0.24)**	−0.06 (−0.48, 0.36)	−0.29 (−0.84, 0.25)	CT_CBT	1.12 (0.27, 4.71)	1.05 (0.48, 2.36)	0.77 (0.24, 2.32)	**2.44 (1.03, 5.99)**	0.89 (0.3, 2.59)	0.72 (0.14, 3.86)	1.26 (0.55, 2.8)	0.73 (0.31, 1.77)
0.7 (−0.23, 1.66)	−0.08 (−0.98, 0.83)	0.63 (−0.22, 1.5)	0.4 (−0.54, 1.35)	0.69 (−0.16, 1.59)	DBT	0.95 (0.23, 3.94)	0.69 (0.11, 4.1)	2.18 (0.48, 10.07)	0.79 (0.14, 4.39)	0.64 (0.07, 5.99)	1.14 (0.32, 3.94)	0.66 (0.16, 2.69)
0.35 (−0.28, 0.97)	−0.43 (−0.92, 0.04)	0.28 (−0.17, 0.73)	0.04 (−0.52, 0.61)	0.33 (−0.17, 0.84)	−0.35 (−1.25, 0.51)	MBCT	0.73 (0.18, 2.75)	2.32 (0.95, 5.64)	0.84 (0.23, 2.97)	0.68 (0.11, 4.39)	1.2 (0.54, 2.61)	0.7 (0.31, 1.55)
−0.58 (−1.35, 0.17)	**−1.36 (−2.13, −0.62)**	−0.64 (−1.31, 0)	**−0.88 (−1.67, −0.14)**	−0.59 (−1.21, 0.01)	**−1.27 (−2.33, −0.3)**	**−0.92 (−1.65, −0.23)**	MCT	3.16 (0.8, 13.6)	1.15 (0.24, 5.53)	0.93 (0.13, 7.32)	1.65 (0.42, 6.55)	0.95 (0.25, 3.9)
0.38 (−0.34, 1.11)	−0.4 (−1.01, 0.2)	0.31 (−0.24, 0.88)	0.08 (−0.48, 0.62)	0.37 (−0.22, 0.97)	−0.32 (−1.31, 0.64)	0.03 (−0.57, 0.63)	**0.96 (0.17, 1.78)**	MM	0.36 (0.09, 1.36)	0.3 (0.04, 1.93)	0.52 (0.19, 1.34)	0.3 (0.11, 0.82)
−0.13 (−0.99, 0.73)	**−0.91 (−1.76, −0.08)**	−0.2 (−0.98, 0.59)	−0.44 (−1.28, 0.42)	−0.15 (−0.82, 0.55)	−0.83 (−1.92, 0.23)	−0.48 (−1.29, 0.34)	0.44 (−0.44, 1.37)	−0.51 (−1.39, 0.38)	PP	0.81 (0.11, 6.11)	1.43 (0.42, 5)	0.83 (0.23, 3.29)
0.15 (−1.2, 1.49)	−0.63 (−1.98, 0.71)	0.09 (−1.22, 1.38)	−0.15 (−1.49, 1.17)	0.14 (−1.09, 1.36)	−0.55 (−2.08, 0.94)	−0.19 (−1.52, 1.13)	0.72 (−0.63, 2.12)	−0.23 (−1.59, 1.13)	0.29 (−1.13, 1.68)	ST	1.75 (0.27, 11.25)	1.02 (0.16, 6.82)
**1.12 (0.52, 1.74)**	0.34 (−0.2, 0.88)	**1.06 (0.63, 1.5)**	**0.82 (0.24, 1.41)**	**1.11 (0.64, 1.61)**	0.42 (−0.37, 1.19)	**0.78 (0.31, 1.25)**	**1.7 (1.02, 2.43)**	**0.74 (0.12, 1.38)**	**1.25 (0.48, 2.04)**	0.97 (−0.33, 2.3)	TAU	0.58 (0.23, 1.48)
**1.39 (0.83, 2)**	**0.61 (0.06, 1.2)**	**1.33 (0.87, 1.82)**	**1.09 (0.51, 1.72)**	**1.38 (0.89, 1.93)**	0.69 (−0.16, 1.57)	**1.05 (0.57, 1.57)**	**1.97 (1.31, 2.7)**	**1.01 (0.38, 1.69)**	**1.53 (0.71, 2.37)**	1.24 (−0.05, 2.61)	0.27 (−0.25, 0.82)	WL

Treatments are listed in alphabetical order. Significant values are printed in bold. The lower left quadrant lists SMDs (95% CrI) for efficacy comparisons between the respective column-defining and row-defining treatment. A positive SMD reflects superiority of the column-defining treatment, and a negative value reflects superiority of the row-defining treatment. Acceptability comparisons as ORs (95% CrI) are listed in the and the upper right quadrant. ORs < 1 favor the column-defining treatment, ORs > 1 favor the row-defining treatment. ACT, acceptance and commitment therapy; ActiveCt, active control group; BA, behavioral activation therapy; CBASP, cognitive behavioral analysis system of psychotherapy; CT/CBT, cognitive therapy/cognitive-behavioral therapy; DBT, dialectical behavioral therapy; MBCT, mindfulness-based cognitive therapy; MCT, meta-cognitive therapy; MM, medication management; PP, positive psychotherapy; ST, schema therapy; TAU, treatment as usual; WL, waitlist.

In terms of acceptability, all third-wave psychotherapies were tolerated as well as CT/CBT. Enrollment in a WL condition was not associated with higher dropout rates (Supplementary Table S14). None of the third-wave psychotherapies was more or less acceptable than TAU or ActiveCt. However, ACT had lower discontinuation rates than MM (OR 0.27 95% CrI 0.08, 0.88). ACT and WL had the highest probabilities of ranking first, meaning that discontinuation was least likely in these conditions, and CBASP and MM had the lowest (Figure 4).

Heterogeneity was estimated to be substantial (*I^2^* = 77%) for efficacy measures and low (*I^2^* = 36%) for acceptability measures (38). Egger’s test for small study effects was significant for two comparisons (MBCT vs. TAU and CBASP vs. MM). Summaries of all estimates and pairwise meta–analyses are reported in Supplementary Tables S13–S15 and Supplementary Text S16. The consistency models showed a better trade-off between model fit and complexity than the inconsistency models supporting the assumption of consistency in the networks (Supplementary Table S17).

Three studies contributed disproportionately high to the overall deviance in the efficacy data (Supplementary Figure S18). Patients in the respective control groups of these trials showed no improvement in depression severity over time resulting in large effect sizes. Their exclusion led to a 51% reduction in heterogeneity while the effect estimates of the NMA remained largely the same (Supplementary Table S19).

Locally, three out of 35 (8.5%) efficacy comparisons showed significant inconsistency (ACT vs. ActiveCt, MCT vs. WL, and BA vs. WL; Supplementary Table S21). Herein, for two of the inconsistent comparisons data was derived from the highly deviant trials mentioned above. For acceptability outcomes, five out of 34 comparisons (14.75%) were inconsistent (Supplementary Table S22).

### 3.4. Sensitivity and subgroup analyses

Given the high proportion of studies investigating persistent and/or treatment-resistant depression (TRD; 28.6%), we performed a sensitivity analysis including only the respective subset of trials (16 trials; Supplementary Figure S23). Effect estimates for all treatments in this subnetwork trended toward a higher efficacy of the third-wave treatments (except for ST) compared with CT/CBT; however, all CrIs overlapped with the those of the full network, and included zero. Exclusion of trials with comorbidities reduced heterogeneity by 23%, and excluding trials with WL controls by 28%. Effect estimates did not change significantly. Inclusion of the covariates risk of bias, baseline depression severity (Supplementary Figure S24), and manualization of the control group did not significantly affect any of the estimates. The exclusion of WL controlled trials increased heterogeneity in the acceptability network by 9.55%. The exclusion of MM controlled trials did not alter effect estimates, except for a 17% increase in heterogeneity in the efficacy network. All subgroup and sensitivity analyses are in Supplementary Tables S19, S20.

### 3.5. Risk of bias assessment

Twenty-eight of the trials (51%) had adequate sequence generation and allocation concealment, 25 (45%) had low risk of bias due to masked assessments, 26 (47%) trials had low attrition rates or performed adequate intent-to-treat analyses and 20 trials (36%) were of low risk of reporting bias, 8 trials (15%) had retrievable protocols. Overall, 15 trials (27%) were rated as low risk of bias overall. Thirty-one (56%) showed some risk of bias and 9 (16%) trials had high risk ratings (Supplementary Figure S25). Certainty in direct comparisons was predominantly low to moderate according to GRADE criteria; only few direct comparisons were of very low certainty while indirect comparisons were rated mostly low and very low certainty (Supplementary materials S26–S29) (39). The references and raw data of all included trials are in Supplementary Table S30 and Supplementary material S31.

## 4. Discussion

In this systematic review and NMA, we firstly identified the scope of the concept of “third–wave psychotherapies” in a systematic database search; and secondly aggregated 55 studies of the resulting treatments of ACT, BA, CBASP, DBT, MBCT, MCT, PP, and ST in over 5,800 patients treated for MDD and compared these treatments with each other and the most common comparators of CT/CBT (CBT for brevity), WL, MM, TAU, and ActiveCt. This is, to our knowledge, the first network meta-analysis that compared third-wave psychotherapies head-to-head, to CBT and other distinct control conditions for this indication.

All third-wave treatments were more efficacious than WL and TAU, except DBT and ST which did not show significant inferiority or superiority. DBT was conceived and is applied predominantly in the context of borderline personality disorder rather than MDD (40). It addresses other symptoms, which makes the lack of efficacy plausible. For schema therapy, the evidence for efficacy in MDD is still too sparse to draw firm conclusions (41, 42). ACT, BA, MBCT, MCT, and PP were more efficacious than ActiveCt conditions which suggests that specific interventions of the treatments are beneficial beyond non-specific psychotherapy effects. However, none of the third–wave treatments were more efficacious than CBT. In the head-to-head comparisons, only MCT was more efficacious than MBCT, DBT, and CBASP. However, the effect of MCT was partly driven by two trials in which patients in the control conditions did not improve on severity measures over the course of the trials, therefore possibly inflating MCT’s effect. In a more recent high quality trial (43) (*n* = 155), MCT did not show superiority over CBT in lowering depression scores in the primary outcome. This highlights the need for sufficiently powered trials that overcome the limitations of early small studies reporting large effect sizes (44).

Our results are in line with previous NMAs that compared different sets of psychotherapy approaches (e.g., CBT, interpersonal, psychodynamic, and problem-solving approaches) for unipolar depression which demonstrated that different psychotherapies are efficacious and acceptable, with only little to no significant differences between them (23, 45). However, to our knowledge, this is the first network meta-analysis that differentiates between individual therapies for this indication and thus allows comparison with each other and with established CBT.

The superior efficacy of an active psychological intervention over WL conditions replicates findings of several previous meta-analyses, in which WL conditions were thought to act as a nocebo (46–48). The exclusion of WL conditions as a whole in our sensitivity analyses did not affect effect estimates but reduced overall heterogeneity in the efficacy network.

In regard to acceptability, none of the third–wave treatments showed lower discontinuation rates than that of CBT or WL conditions. Unexpectedly, in absolute terms, WL conditions in our analysis had one of the lowest attrition rates. Previous research on psychotherapy trials has shown that an average of 20% of patients with depression withdraw from participation in psychotherapy trials before the endpoint (49). Moreover, study discontinuation in psychotherapy trials is partly due to the early benefit from treatment (50). If participants who benefit early are excluded from the analysis, the actual effect size may be underestimated and non–acceptability may be overestimated. In contrast, participants randomized to WL conditions may wait until the end of the trial because they continue to require treatment. The opposite effect of lower acceptability, that is, higher attrition, of psychological interventions compared with WL has been shown elsewhere (23).

Notably, all studies were published in the past 20 years, which reflects the dynamic developments in the field of psychotherapy for depression. The developments have been driven and informed by high rates of treatment resistance to well-established psychotherapies, such as CBT and psychodynamic approaches. Our findings do not provide evidence for superiority or inferiority of third–wave approaches over CBT. What might be the reasons for this? Firstly, equal efficacy between treatments does not render them equal or obsolete. Equally effective therapies can and should co-exist since population-level efficacy does not translate into individual response probabilities. As with first-line antidepressant medication, patients need alternatives to choose from. Secondly, global equal efficacy might obscure heterogeneity in the spectrum of depressive disorders which is reflected in treatment resistance. Persistently depressed patients, for example, often present with distinctive complicating features (51, 52). In our sensitivity analysis that included only studies in TRD and PDD populations, third–wave psychotherapies showed a trend toward superiority compared to CBT. Therefore, patient-level variables that mediate differential treatment responses need to be identified, such as experiential avoidance mediating response in studies comparing CBT and ACT (53) or childhood trauma mediating CBASP effects (54). Efforts in this direction are already being taken, by identifying response predictors that can be used by machine learning tools in the development of clinical decision support systems for psychotherapy (55).

In line with the notion of differential benefits between patients, a recent meta–analysis of variance ratios revealed significant heterogeneity in the treatment effects of CBT and third-wave psychotherapies. A finding which helps promote efforts to implement algorithms toward personalized psychotherapy (56) or process–based approaches for CBT (17).

Our study has certain strengths but also several limitations. One of its strengths is the rigorous inclusion criteria (clinical diagnosis of depression) which homogenizes the included samples. However, regarding transitivity of the network, some violations may arguably hold (57). For example, several trials included only patients with a history of PDD or TRD. The included samples consisted of moderately to severely depressed patients and for both conditions antidepressant medication is recommended. Moreover, concomitant treatment has been found to be superior to single treatment (58). It is therefore ecologically valid that most of the included trials intentionally applied or tolerated combined treatments. However, not all of them did and precision of effect sizes might be affected by the heterogeneity of treatment regiments. Possible violations of transitivity may result in statistical inconsistency (59), some of which was observed in our dataset. In our sensitivity analyses we accounted for these possible sources of inconsistency and were able to substantially reduce heterogeneity by removing outliers. Effect sizes were largely robust to our sensitivity analyses. A further limitation lies in the variety of control conditions. Despite their similarities, homonymous control conditions of different trials may offer substantially different treatments, especially if non–manualized, like TAU (60, 61). Lastly, only a 27% fraction of trials had a low risk of bias rating and the certainty in estimates was mostly low. In future studies, focus should preferentially shift toward showing that a “new” therapy is more efficacious, acceptable time or cost efficient than an established one in a patient group or subgroup.

Here we present a first NMA that integrates a systematic definition of the scope of third–wave therapies and the inclusion of data under rigorous criteria. In summary, the majority of third–wave psychotherapies can be regarded as more efficacious than TAU or WL. However, to date, there is no evidence suggesting that they overcome the limitations in efficacy of acceptability of CBT.

## Data availability statement

The original contributions presented in the study are included in the article/Supplementary material, further inquiries can be directed to the corresponding author.

## Author contributions

CS and CH had full access to all data in the study and take responsibility for the integrity of the data and the accuracy of the data analysis. CS, SK, AG, PS, and E-LB conceived and designed the study. CS, CH, SK, and AG selected the articles and extracted the data. CS and CH analyzed the data. CS, SK, AG, PS, and E-LB interpreted the data. CS and SK prepared the original draft of the manuscript. All authors contributed to the article and approved the submitted version.
