# Supplementary material for: Efficacy and acceptability of third-wave psychotherapies in the treatment of depression: a network meta-analysis of controlled trials
Source: Front Psychiatry. 2023 Oct 5;14:1189970. doi: 10.3389/fpsyt.2023.1189970 (PMC10585267; doi:10.3389/fpsyt.2023.1189970)
Supplement: Supplementary file 1 [file Data_Sheet_1.docx]

**Supplementary Online Content**

**Appendix**

Table of contents

[Text S1: Changes to the protocol 2](#_Toc146525398)

[Text S2: Search terms 2](#_Toc146525399)

[Text S3: Clustering of studies based on control conditions 3](#_Toc146525400)

[Text S4: Hierarchy of outcome measures 4](#_Toc146525401)

[Frequency distribution of scales used in the data set 4](#_Toc146525402)

[Table S5: Severity cut-offs 4](#_Toc146525403)

[Text S6: Modeling 6](#_Toc146525404)

[Arm-data preparation 6](#_Toc146525405)

[Model compilation and MCMC simulation 6](#_Toc146525406)

[Prior distributions 6](#_Toc146525407)

[Convergence status 6](#_Toc146525408)

[Text S7: Risk of Bias assessment 7](#_Toc146525409)

[Dichotomization for inclusion of the Risk of Bias covariate into meta-regression 7](#_Toc146525410)

[Summary of the Risk of Bias rating 7](#_Toc146525411)

[Table S8: Number of studies retrieved per treatment per database 8](#_Toc146525412)

[Figure S9: Age distribution per comparison 9](#_Toc146525413)

[Table S10: Distribution of countries 10](#_Toc146525414)

[Table S11: Overview of study characteristics 11](#_Toc146525415)

[1. ACT trials included in the analysis 11](#_Toc146525416)

[2. BA trials included in the analysis 12](#_Toc146525417)

[3. CBASP studies included in the analysis 14](#_Toc146525418)

[4. MBCT studies included in the analysis 15](#_Toc146525419)

[5. MCT studies included in the analysis 17](#_Toc146525420)

[6. DBT studies included in the analysis 17](#_Toc146525421)

[7. PP studies included in the analysis 18](#_Toc146525422)

[8. SCHEMA studies included in the analysis 18](#_Toc146525423)

[Table S12: Distributions of studies over comparisons 20](#_Toc146525424)

[Tables S13 and S14: Relative effects tables 22](#_Toc146525425)

[Table S13: Efficacy 22](#_Toc146525426)

[Table S14: Acceptability 23](#_Toc146525427)

[Table S15: Results of pairwise meta-analyses - Efficacy data 25](#_Toc146525428)

[Table S9: Results of pairwise meta-analyses - Acceptability data 31](#_Toc146525429)

[Text S16: Summary of random effects pairwise meta–analyses 38](#_Toc146525430)

[Table S17: Model comparison consistency vs. inconsistency models 38](#_Toc146525431)

[Figure S18: Leverage plots 39](#_Toc146525432)

[Tables S19 and S20: Subgroup and sensitivity analyses 40](#_Toc146525433)

[Table S19: Efficacy data 40](#_Toc146525434)

[Table S20: Acceptability data 42](#_Toc146525435)

[Table S21: Table of node-splitting analysis for efficacy data 44](#_Toc146525436)

[Table S22: Table of node-splitting analysis for acceptability data 47](#_Toc146525437)

[Figure S24: Severity distribution per comparison 52](#_Toc146525438)

[Figure S25: Results of the Risk of Bias assessment 53](#_Toc146525439)

[Text S26: GRADE criteria (16) 54](#_Toc146525440)

[Table S29: Results of the GRADE rating 57](#_Toc146525441)

[Table S30: Individual study data and effect sizes 61](#_Toc146525442)

[References S31: References for the included studies 65](#_Toc146525443)

[References 68](#_Toc146525444)

# Text S1: Changes to the protocol

1. Review question: Interpersonal therapy was excluded as a comparator since it was not a third-wave psychotherapy and not frequently used as a comparator, excessively complicating the network.

2. Search dates: Due to COVID-19 restrictions, resources within the department had to be reallocated forcing our research activities into a hiatus. This is why we decided to update the searches at a later point, refreshing our data set.

3. Inclusion criteria: The comparator set was more modified to include: waitlist, treatment as usual, medication management, active controls, CBT.

4. Population: Healthy controls were not included since the included designs needed to compare patient samples randomized to treatment conditions.

5. Risk of Bias assessment: Risk of bias was assessed according to the RoB2 tool as described below.

6. Subgroup analyses were modified: No influence of age of the sample was included since this is subject to ecological bias. Subclinical symptomatology was not included since only clinically depressed populations were included. Duration of treatment/number of session were not included as covariates. Instead, we were interested in the covariates: Risk of bias, manualization, treatment resistance/persistent depressive populations.

# Text S2: Search terms

**CENTRAL**

ID Search Hits

#1 (("depression" OR "depressive disorder") AND dialectical behavior):ti,ab,kw (Word variations have been searched)

#2 (("depression" OR "depressive disorder") AND ("behavioral activation" OR "behavioural activation") ):ti,ab,kw in Trials (Word variations have been searched)

#3 (("depression" OR "depressive disorder") AND ("metacognitive therapy") ):ti,ab,kw in Trials (Word variations have been searched)

#4 (("depression" OR "depressive disorder") AND ("mindfulness-based cognitive therapy") ):ti,ab,kw in Trials (Word variations have been searched)

#5 (("depression" OR "depressive disorder") AND ("schematherapy" OR "schema therapy") ):ti,ab,kw in Trials (Word variations have been searched)

#6 (("depression" OR "depressive disorder") AND ("CBASP" OR "cognitive behavioral analysis system of psychotherapy") ):ti,ab,kw in Trials (Word variations have been searched)

#7 (("depression" OR "depressive disorder") AND ("positive psychotherapy") ):ti,ab,kw in Trials (Word variations have been searched)

#8 (("depression" OR "depressive disorder") AND ("Acceptance and commitment therapy" OR "Acceptance and commitment psychotherapy") ):ti,ab,kw in Trials (Word variations have been searched)

#9 (("depression" OR "depressive disorder") AND ("emotion-focused therapy" OR "emotion-focused psychotherapy") ):ti,ab,kw in Trials (Word variations have been searched)

#10 (("depression" OR "depressive disorder") AND ("wellbeing therapy" OR "wellbeing psychotherapy") ):ti,ab,kw in Trials (Word variations have been searched)

**PubMed**

"cognitive behavioral analysis system of psychotherapy"[All Fields] OR "CBASP"[All Fields]

"acceptance and commitment"[All Fields] AND ("depressive disorder"[MeSH Terms] OR ("depressive"[All Fields] AND "disorder"[All Fields]) OR "depressive disorder"[All Fields] OR "depression"[All Fields] OR "depression"[MeSH Terms])

"schema therapy"[All Fields] AND ("depressive disorder"[MeSH Terms] OR ("depressive"[All Fields] AND "disorder"[All Fields]) OR "depressive disorder"[All Fields] OR "depression"[All Fields] OR "depression"[MeSH Terms])

"behavioral activation"[All Fields] AND ("depressive disorder"[MeSH Terms] OR ("depressive"[All Fields] AND "disorder"[All Fields]) OR "depressive disorder"[All Fields] OR "depression"[All Fields] OR "depression"[MeSH Terms])

"metacognitive therapy"[All Fields] AND ("depressive disorder"[MeSH Terms] OR ("depressive"[All Fields] AND "disorder"[All Fields]) OR "depressive disorder"[All Fields] OR "depression"[All Fields] OR "depression"[MeSH Terms])

"mindfulness based cognitive therapy"[All Fields] AND ("depressive disorder"[MeSH Terms] OR ("depressive"[All Fields] AND "disorder"[All Fields]) OR "depressive disorder"[All Fields] OR "depression"[All Fields] OR "depression"[MeSH Terms])

dialectical behavior therapy AND ("depressive disorder"[MeSH Terms] OR ("depressive"[All Fields] AND "disorder"[All Fields]) OR "depressive disorder"[All Fields] OR "depression"[All Fields] OR "depression"[MeSH Terms])

positive psychotherapy AND ("depressive disorder"[MeSH Terms] OR ("depressive"[All Fields] AND "disorder"[All Fields]) OR "depressive disorder"[All Fields] OR "depression"[All Fields] OR "depression"[MeSH Terms])

("functional analytic psychotherapy") AND ((depression OR depressive))

"integrative behavioral couple therapy"

"compassion focused therapy"

# Text S3: Clustering of studies based on control conditions

Since different studies used a variety of control conditions, we clustered control conditions into the following categories based on author consensus: 1. Cognitive therapy/ cognitive behavioral therapy (CT/CBT): For studies in which cognitive (behavioural) therapy as active control condition. Trials that stated they applied CT or CBT but did not quote a manual were also entered into this category. 2. Active control condition (ActiveCt): Summarizes control conditions like psychoeducation, supportive therapy etc.. Active control conditions can be characterized as structured interventions that are not specific to the disorder and do not apply the assumed effective therapy components of the intervention group but equal the intervention in dose/intensity of contact and are structured within the trial. 3. Waitlist (WL): All control conditions that randomized participants to a condition where they would receive the treatment in question after a given period of time or conditions where participants received no treatment at all. 4. Medication management (MM): All control conditions that included a protocol/algorithm for an antidepressant regimen structured within the trial but no psychological intervention. 5. Treatment as usual (TAU): Control conditions that were not structured within the trial but had patients pursue treatment independently.

# Text S4: Hierarchy of outcome measures

Deviation from protocol: we adjusted the hierarchy of outcome measures given that self-report measures might be biased dur to participants being in the known of their treatment. We therefore prioritized the outcome measures according to the following hierarchy:

1. HRSD
2. MADRS
3. IDS-C
4. QIDS-C
5. BDI
6. IDS-SR
7. QIDS-SR
8. PHQ-9/-8
9. CES-D
10. EPDS

## Frequency distribution of scales used in the data set

| measure | Number of comparisons |
| --- | --- |
| BDI | 30 |
| CESD | 0 |
| EPDS | 1 |
| HRSD17 | 21 |
| HRSD21 | 1 |
| HRSD24 | 8 |
| HRSD28 | 1 |
| IDSC | 0 |
| IDSSR | 2 |
| MADRS | 3 |
| PHQ9 | 4 |
| QIDSC | 1 |
| Sum | 72 |

#

# Table S5: Severity cut-offs

In order to determine the severity cutoffs for depression we used the table provided in <http://www.ids-qids.org/interpretation.html> (accessed July, 1^st^ 2020) and converted all HRSD (-17,- 21 and -24 item versions), IDS-SR, QIDS-SR scores to a 4-level severity rating. For the remainder of scales we used cutoffs as suggested in the cited publications.

In one case which used the 28-item version of the HRSD(1), we decided to base baseline severity ratings on baseline BDI data. A second case, which used QIDS-C outcome ratings, MADRS baseline scores (but not outcome scores) were available and used for the assessment of baseline severity (2).

.

| title of scale | Abbreviation | | grade of severity | | | | | |
| --- | --- | --- | --- | --- | --- | --- | --- | --- |
|  | |  | | 1 | 2 | 3 | 4 |  |
| Beck Depression Inventory (3) | | BDI/BDI-II | | 0 - 13 minimal | 14 -19 mild | 20 – 28 moderate | ≥ 29 severe |  |
| Center of Epidemiologic Studies – Depression Scale (4, 5) | | CES-D | | 0 - 9 none | 10 - 15 mild | 16 - 24 moderate | ≥ 25 severe |  |
| Edinburgh postnatal depression scale (6)^[[1]](#footnote-1)^ | | EPDS | | 0 – 8 none |  | NA | NA |  |
| Hamilton Rating Scale for Depression-17 item (7) | | HRSD-17 | | 0 – 7 none | 8 – 19 mild | 20 – 25 moderate | ≥ 26 severe |  |
| Inventory of Depressive Symptomatology, Clinician Rated | | IDS-C | | 0 - 13 none, 14 - 22 mild | 23 - 30 moderate | 31 - 38 moderate to severe | 39+ severe |  |
| Inventory of Depressive Symptomatology, Self-report (8) | | IDS-SR | | 0 - 15 none, 16 - 24 mild |  | 25 - 32 moderate, 33 - 40 moderate to severe | 41+ severe |  |
| Montgomery–Asberg Depression Rating Scale (9-11) | | MADRS | | 0 – 6 none | 7 – 17mild | 18 – 30 moderate | ≥ 31 - 60 severe |  |
| Patient Health Questionnaire (12) | | PHQ-9 | | 1 - 4 minimal, 5 - 9 mild | 10 - 14 moderate | 15 - 19 moderately severe | 20 - 27 severe |  |
| Quick Inventory of Depressive Symptomatology. Self-report (13) | | QIDS-SR | | 0 – 5 none, 6 – 10 mild | 11 – 15 moderate | 16 – 20 severe | and 21 – 27 very severe |  |

# Text S6: Modeling

## Arm-data preparation

In multi-arm trials. comparing treatments A.B and C. standard errors of the base arm of a reference treatment (B) were determined according to

$se_{B}=\sqrt{\left( se_{\left( AB \right)}^{2}+se_{\left( CB \right)}^{2}-se_{\left( AC \right)}^{2} \right)/2}$.

**Model compilation and MCMC simulation**

Four Markov chain simulations were run to approximate the posterior distributions for the basic parameters with a discarded burn-in of 5000 iterations and 100 000 inference iterations per chain with a thinning factor of ten.

**Prior distributions**

Choice of priors (14): Prior distributions were specified for all relative effects of the basic parameters. $d$_x.y_. the random-effects variance σ^2^ and the baseline effects μ. The random-effects variance is uniformly distributed with parameters σ $\sim$ U(0,u). The prior variance u of the random-effects variance distribution is derived from maximum likelihood estimators as described in the original publication (14). For likelihood functions please refer to the original publication. Relative effects and baseline effect d_x.y_ and μ are normally distributed with parameters $d$ _x.y_ and μ $\sim$ N(n, η^2^) (14). The resulting prior distributions for our random effects meta-analysis data were

For efficacy data:

$$\sigma\sim\mathcal{U}\left( 0, 5.77 \right)$$

$$d_{x.y}. \mu\sim\mathcal{N}\left( 0, \left( 7.49\times{10}^{3} \right) \right)$$

For acceptability data:

$$\sigma\mathcal{\sim U}\left( 0, 3.22 \right)$$

$$d_{x.y}.\mu\sim\mathcal{N}\left( 0, \left( 2.333\times{10}^{3} \right) \right).$$

**Convergence status**

Convergence status was assessed by density. Gelman-Rubin-Brooks plots and by potential scale reduction factors. which were <1.001 for the efficacy model and <1.002 for acceptability models. Indicating good convergence of the simulations.

# Text S7: Risk of Bias assessment

In assessing risk of bias, we adapted the guidelines in the Revised Cochrane risk-of-bias tool for randomized trials (RoB 2) (15) to the requirements of RCTs in the field of psychological interventions.

We assessed the domains of risk of bias arising from the randomization process (sequence generation, allocation concealment, differences in baseline characteristics), bias in measurement of the outcome (blinding of outcome assessment), bias due to missing outcome data, and bias in selection of the reported result.

Risk of bias due to randomization was rated low if the report stated the process of generating the randomization sequence and the concealment of allocation. If the report stated that external personnel was responsible for randomization and allocation risk of bias was rated low. Risk of bias was rated high if there was no randomization, quasi-randomization, allocation followed date of admission or was in any other way predictable. Reports stating that participants were randomized but don’t provide detailed information were rated to have some risk of bias.

Given that blinding of therapists and participants is virtually impossible in controlled trials on psychological interventions, we did not assess risk of bias due to unmasking of participants.

However, the lack of masking on the side of the participants is reflected in the ratings of risk of bias due to masking of outcome assessment. All trials that compared an active intervention to a waitlist condition, medication only or treatment as usual AND used a patient self-report as the only outcome were rated to as high risk of bias because participants were aware of their treatment allocation. Trials that used two active treatments in which both groups received an intervention AND only used self-report measures were rated to be at least of some risk of bias. Trials describing unmasked outcome assessment by clinicians were also rated high risk of bias. Low risk of bias was coded if the personnel assessing outcomes was described as masked to treatment allocation.

Risk of bias due to missing outcome data: Low: if adequately imputed and sensitivity analyses reported for a range of possible outcomes, if <= 25% AND balanced between groups (less than 5% difference when active comparison is used or 10% if compared against wait-list). Some: if no information was given. High: Higher than 25% missing data OR in total lower than 25% but imbalanced in number between groups OR in total lower than 25% and imbalanced regarding the reasons for discontinuations between groups. In the latter case the reasons for dropping out are likely associated with the true value of the outcome or the treatment condition and therefore the outcome is more likely to be biased (i.e. dropping out due to dissatisfaction with allocation to treatment as usual condition).

Risk of bias due to selective reporting: Low if protocol is available (published previously and procedures adhered to protocol), if the trial was registered in official registry before data collection and registered protocol contains the outcomes specified which are in accordance with the outcomes published. Some: no protocol available, all expected outcomes reported. High: some outcomes that are relevant for the review and meta-analysis are not reported, data that was planned to be collected is not reported.

**Dichotomization for inclusion of the Risk of Bias covariate into meta-regression**

For each category low risk of bias was coded as zero, some risk of bias was coded as 1 and high risk was coded as 2. Values of all four domains were added and their median assessed. If the sum of risk of bias was larger than the median risk of bias of all studies the study was categorized as high risk of bias, if it was lower or equal than the median it was categorized as low risk of bias.

**Summary of the Risk of Bias rating**

Selection bias was low for 28 studies, high for eight, and intermediate for 19 studies. Twenty–five studies had a low risk of masking bias, 11 had a high risk, and 19 had some risk because they either compared against WL, used self–report instruments, and/or raters were not blinded. Twenty–five studies showed high rates of attrition without adequate imputation of missing values, four studies showed some concern, and 26 had a low risk of attrition bias. Seven studies were at a high risk of reporting bias because of missing data, 28 had an intermediate level of risk, and 20 had a low risk. A graphical overview can be found in figure S25.

# Table S8: Number of studies retrieved per treatment per database

|  | MEDLINE | PSYC_INFO | CENTRAL | WoS |  |
| --- | --- | --- | --- | --- | --- |
|  |  |  |  |  |  |
| ACT | 316 | 95 | 437 | 306 |  |
| BA | 555 | 265 | 816 | 740 |  |
| CBASP | 103 | 80 | 98 | 168 |  |
| MBCT | 381 | 138 | 435 | 263 |  |
| DBT | 40 | 34 | 177 | 24 |  |
| SCHEMA | 38 | 18 | 40 | 77 |  |
| MCT | 66 | 22 | 91 | 94 |  |
| PP | 30 | 5 | 50 | 26 |  |
| WBT | 5 | 9 | 88 | 29 |  |
| FAP | 34 | 38 | 5 | 39 |  |
| IBCT | 29 | 37 | 17 | 10 |  |
| CFT | 16 | 10 | 59 | 88 |  |
| SUM | 1613 | 844 | 2313 | 1864 | 7971 |
|  |  |  |  |  |  |
|  |  |  |  | Duplicates | 3886 |
|  |  |  |  |  | 4085 |

# Figure S9: Age distribution per comparison

**Figure S9. Boxplot of mean age of participants per comparison**. Legend: ACT = Acceptance and commitment therapy, ActiveCt = Active control group, BA = Behavioral activation therapy, CBASP = Cognitive behavioral analysis system of psychotherapy, CT/CBT =Cognitive therapy/Cognitive behavioral therapy, DBT = Dialectical behavioral therapy, MBCT = Mindfulness-based cognitive therapy, MCT = Meta-cognitive therapy, MM= Medication management, PP = Positive psychotherapy, ST = Schema therapy, TAU = Treatment as usual, WL= Waitlist.

# Table S10: Distribution of countries

| \| Country \| absolute \| % \| \| --- \| --- \| --- \| \| USA \| 14 \| 25.45 \| \| Canada \| 1 \| 1.82 \| \| Brazil \| 1 \| 1.82 \| \| UK \| 6 \| 10.91 \| \| Germany \| 5 \| 9.09 \| \| Netherlands \| 4 \| 7.27 \| \| Sweden \| 3 \| 5.45 \| \| Austria \| 1 \| 1.82 \| \| Denmark \| 1 \| 1.82 \| \| Norway \| 1 \| 1.82 \| \| Finland \| 1 \| 1.82 \| \| Spain \| 1 \| 1.82 \| \| Italy \| 1 \| 1.82 \| \| Australia \| 2 \| 3.64 \| \| New Zealand \| 1 \| 1.82 \| \| Korea \| 1 \| 1.82 \| \| Iran \| 10 \| 18.18 \| \| Indonesia \| 1 \| 1.82 \| |  |  |
| --- | --- | --- | --- | --- | --- | --- | --- | --- | --- | --- | --- | --- | --- | --- | --- | --- | --- | --- | --- | --- | --- | --- | --- | --- | --- | --- | --- | --- | --- | --- | --- | --- | --- | --- | --- | --- | --- | --- | --- | --- | --- | --- | --- | --- | --- | --- | --- | --- | --- | --- | --- | --- | --- | --- | --- | --- | --- | --- | --- |

# Table S11: Overview of study characteristics

| 1. ACT trials included in the analysis | | | | | | |
| --- | --- | --- | --- | --- | --- | --- |
| Authors | Mean age (sd), female gender | Sample | Conditions (n) | Medication | Length of treatment (n dropout) | Outcome measures |
| A-Tjak et al. 2018 | 40.45 (12.55) 50% | DSM-IV criteria for MDD, age 18-65 years | 1. Control: Cognitive behavioral therapy (n=38) | Yes (65%) | 20 sessions, 45-55 min, 30 weeks (n=6) | SCID, HRSD, QIDS |
|  | 42.52 (12.21), 52.7% |  | 2. Acceptance and commitment therapy (n=44) | Yes (63%) | 20 sessions, 45-55 min, 30 weeks (n=6) |  |
| Far et al. 2017 | 25.2 (4.2), 100% | DSM-IV-TR criteria for MDD | 1. Control: Cognitve therapy (n=9) | NA | 12 sessions, 6 weeks (n=0) | SCID, BDI-II |
|  |  |  | 2. Acceptance and commitment therapy (n=10) | NA | 12, 6 weeks (n=0) |  |
| Folke et al. 2012 | 46.25 (7.88), 81.3% | DSM-IV criteria for unipolar depressive disorder, unemployment/temporary sick leave, age 18-65 years | 1. Control: Treatment as usual (n=16) | NA | 6 weeks (n=6) | BDI |
|  | 40.56 (10.14), 94.4% |  | 2. Acceptance and commitment therapy (n=18) | NA | 1 individual, 60-90 min; 5 group, 120-180 min, 6 weeks (n=4) |  |
| Kyllonen et al. 2018 | 50 (12), 75% | ICD-10 depressive disorder diagnosis, age 18-65 years | 1. Control: Waitlist, no treatment (n=55) | No | 6 weeks (n=4) | Diagnostic interview not specified, BDI-II |
|  |  |  | 2. Acceptance and commitment therapy (n=60) | No | 6 sessions, 60 min, 6 weeks (n=0) |  |
| Lappalainen et al. 2015 | 51.9 (12.88), 68.4% | DSM-IV-TR criteria for MDE, stable medication regimen, age ≥ 18 years | 1. Control: Waitlist (n=20) | Yes (20.5%) | 7 weeks (n=0) | Custom structured interview, BDI-II |
|  |  |  | 2. Internet-based Acceptance and commitment therapy (n=18) | Yes (20.5%) | 6 modules, 6 weeks (n=1) |  |
| Ledari et al. 2018  100% TRD | 36.14 (4.05), 85.71% | DSM-IV criteria for MDD, BDI-II score > 14, treatment-resistant, stable medication regimen, age 18-60 years | 1. Control: Brief Behavioral activation (n=3) | Yes | 12 sessions, 12 weeks (n=3) | SCID, BDI-II |
|  |  |  | 2. Acceptance and commitment therapy (n=4) | Yes | 12 sessions, 12 weeks (n=2) |  |
| Zemestani et al. 2020 | 24.45 (4.2)m 73% | physical disability (25%–50% impairment); DSM-5 criteria for moderate or severe depression, BDI-II total score>20, at least a high school diploma | 1. Control: Active control (general psychoedu- cation regarding depression and coping strategies) (n=29) | NA | 8 sessions, 8 weeks (n=1) | SCID, BDI-II |
|  |  |  | 2. Acceptance and commitment therapy (n=23) | NA | 8 sessions, 90 min, 8 weeks (n=7) |  |
| Zettle et al. 2011 | 41.3 (sd), 100% | BDI score ≥ 20, HRSD ≥14, T-score ≥ 70 on Minnesota Multiphasic Personality Inventory (Hathaway & McKinley, 1942) | 1. Control: Cognitive therapy (n=13) | No | 12 sessions, 90 min, 12 weeks (n=0) | BDI-II |
|  |  |  | 2. Acceptance and commotimtent therapy (n=12) | No | 12 sessions, 90 min, 12 weeks (n=0) |  |
| 2. BA trials included in the analysis | | | | | | |
| Authors, class of control | Mean age (SD), female gender | Sample | Conditions (n) |  | Length of treatment (n dropout) | Outcome measures |
| Arjadi et al. 2018 | 24.5 (4.93), 81% | PHQ-9 score ≥ 10, DSM-V criteria for MDD or persistent depressive disorder, age ≥ 16 years | 1. Control: Internet-based psychoeducation (n=154) | No | 10 weeks (n=9) | SCID, PHQ-9, IDS-SR |
|  |  |  | 2. Internet-based Behavioral activation treatment (n=159) | No | 8 modules, 30-45 min, 10 weeks (n=39) |  |
| Bolinski et al. 2018 | 35.3 (12.6), 52.2% | Diagnosis of depression (SCID); BDI-II score > 20 | 1. Control: Cognitive behavioral therapy (n=23) | No | 24 sessions, 16 weeks (n=0) | SCID, BDI-II |
|  | 42.2 (15.9), 50% |  | 2. Behavioral activation (n=20) | No | 24 sessions, 16 weeks (n=0) |  |
| Cullen et al., 2003 | 37.9 (20-61), 35% | Criteria for MDD, BDI-II score ≥ 20, Revised HRSD ≥ 14 | 1, Control: Waitlist (n=8) | No | 6 weeks (n=0) | SCID, BDI-II, RHRSD |
|  |  |  | 2. Behavioral activation (n=9) | No | 10 weeks (n=3) |  |
| Dimidijan et al. 2006  30% chronic | 39.90 (10.97), 66.0% | DSM-IV criteria for MDD, BDI score ≥ 20, HRSD score ≥ 14; aged 18-60 years, majority recurrent (57%) or chronic (30%). | 1. Control: Cognitive therapy (n=45) | No | 24 sessions, 50 min, 16 weeks (n=6) | SCID, BDI, HRSD |
|  |  |  | 2. Control: Antidepressant pharmacotherapy (n=100) | Yes | 28 sessions, 30-45 min, 16 weeks (n=44) |  |
|  |  |  | 3. Control: Pill Placebo (n=53), not included in analysis | No | 28 sessions, 8 weeks |  |
|  |  |  | 4. Behavioral activation (n=43) | No | 24 sessions, 50 min, 16 weeks (n=7) |  |
| Ekers et al. 2011 | 44.72 (24-63), 62% | ICD-10 diagnosis for depression, stable/no medication (within 6 weeks) | 1. Control: Treatment as usual (n=24) | Yes | 12 weeks (n=2) | Clinical Interview Schedule Revised,  BDI |
|  |  |  | 2. Behavioral activation (n=23) | Yes | 12 sessions, 12 weeks (n=7) |  |
| Fereidooni et al. 2015 | 32.7 (6.9), 0% | Diagnosis of an affective disorder, except bipolar disorder, aged 20-50 years | 1. Control: Pharmacotherapy (n=8) | Yes | 4 weeks (insufficient information) | SCID, BDI-II, BADS |
|  | 31.3 (6.4), 0% |  | 2. Control: Alternative treatment (movie and discussion) (n=8) | Yes | 7 sessions, 4 weeks (insufficient information) |  |
|  | 32.6 (6.8), 0% |  | 3. Group-based Behavorial activation (n=8) | Yes | 7 sessions, 4 weeks (insufficient information) |  |
| Hemmany et al. 2019 | 38.7 (10.8) 87% | DSM-IV or ICD-10 criteria for MDD, on antidepressant medications for at least 2 months,  HRSD ≥ 15, BDI-II ≥ 20, aged 18 - 60 years | 1. Control: Treatment as usual (Antidepressant therapy,naturalistic treatment) (n=26) | Yes | 12 weeks (n=16) | MINI,HRSD, BDI-II |
|  |  |  | 2. Control:Trial based Cognitive behavioral therapy (n=26) | Yes | 12 sessions, 12 weeks (n=13) |  |
|  |  |  | 3. Behavioral activation (n=24) | Yes | 12 sessions, 12 weeks (n=10) |  |
| Hopko et al. 2003 | 30.5 (9), 36% | Diagnosis of MDD, inpatient setting | 1. Control: Supportive psychotherapy (n=15) | Yes | >6 sessions, 20 min, 2 weeks/until hospital discharge (insufficient infromation) | BDI |
|  |  |  | 2. Behavioral activation for inpatients (n=10) | Yes | >6 sessions, 20 min, 2 weeks/until hospital discharge (insufficient inforamtion) |  |
| Kanter et al. 2014 | 38.1 (10.8), 79.1% | DSM-IV diagnosos of MDD, HRSD-17 ≥ 16; LatinX, aged 18-65 years | 1. Control: Treatment as usual (n=22) | Yes (9%) | 12 sessions, 50 min, 12 weeks (n=10) | MINI, QIDS-SR, PHQ-9 |
|  |  |  | 2. Behavioral activation (n=21) | Yes (14%) | 12 sessions, 50 min, 12 weeks (n=5) |  |
| Lee et al. 2021 | 36.1 (17), 58% | DSM-IV diagnosos of MDD or dysthymic disorder. Aged  18 - 80 years. | 1. Control: Treatment as usual (n=33) | Yes (85% ) | 10 weeks (n=17) | SCID or MINI, HRSD, CES-D |
|  |  |  | 2. Behavioral activation (groups of 2-5) (n=31) | Yes (94% ) | 10 sessions, 10 weeks (n=9) |  |
| Ly et al. 2014 | 36.1 (10.8) 70% | DSM-IV diagnosis of MDD, ≥ 1 episode of partial remission, PHQ-9 score ≥5 , age ≥ 18 years | 1. Control: Smartphone-based Mindfulness-based cogntitive therapy (n=41) | Yes (34.1%) | 8 weeks (n=5) | MINI, BDI-II, PHQ-9 |
|  |  |  | 2. Smartphone-based Behavioral activation (n=40) | Yes (40%) | 8 weeks (n=4) |  |
| Moradveisi et al. 2013 | 31.37 (8.97), 85% | DSM-IV diagnosis MDD; BDI-II score ≥ 19; HRSD ≥ 14 | 1. Control: Antidepressant pharmacotherapy (n=50) | Yes | 8 sessions, 10-20 min, 12 weeks (n=15) | SCID, BDI-II, HRSD |
|  |  |  | 2. Behavioral activation (n=50) | No | 16 sessions, 12 weeks (n=5) |  |
| Nyström et al. 2017 | 42.0 (13.5), 76% | DSM-IV diagnosis of mild to moderate depression, MADRS-SR score 15-35, aged ≥ 18 years | 1. Control: Treatment as usual (n=53) | Yes | 12 weeks (n=2) | SCID, MADRS-S, PHQ-9 |
|  |  |  | 2. Control: Physical activity (n=121) (not included in analysis) | Yes | 8 modules, 12 weks (n=14) |  |
|  |  |  | 3. Behavioral activation: BA-L, based on Lewinsohn's model, 1974 (n=63) | Yes | 8 modules, 12 weeks (n=8) |  |
|  |  |  | 4. Behavioral activation: BA-M, based on Martell et al., 2010 (n=49) | Yes | 8 modules, 12 weeks (n=2) |  |
| O'Mahen et al. 2014 | mean (sd), 100% | ICD-10 criteria for MDD, depressed postpartum women (within one year prior study), EPDS score > 12, aged > 18 years | 1. Control: Treatment as usual, peer support via chat system (n=42) | Yes (66%) | 17 weeks (n=8) | Diagnostic interview not specified, EPDS |
|  |  |  | 2. Web-based Behavioral activation (n=41) | Yes (59%) | 12 modules, 17 weeks (n=3) |  |
| Richards et al. 2017 | 43.5 (14.1), 66% | DSM-IV criteria for MDD; aged ≥ 18 years | 1. Control: Cognitive-behavioral therapy (n=175) | Yes (79%) | 20 (+4 optional sessions), 60 min, 16 weeks (n=24) | SCID, PHQ-9 |
|  |  |  | 2. Intervention: Behavioral activation (n=189) | Yes(78%) | 20 (+4 optional sessions), 60 min, 16 weeks (n=36) |  |
| Soucy et al. 2017 | 34.74 (12.24), 75% | PHQ score mild to moderate, aged 18–65, DSM-IV criteria for MDD not mandatory but met by majority. | 1. Control: Waitlist, no treatment (n=20) | Yes (31,6%) | 16 sessions, 8 weeks (n=2) | MINI, PHQ-9 |
|  | 33.16 (9.35), 63% |  | 2. Intervention: Physical activity, psychoeducation (n=20) (not included) |  | mean sessions 3.61, 8 weeks (n=4) |  |
|  | 30.20 (8.23), 80% |  | 3. Behavioral activation self-help manual (n=20) | Yesv (25%) | mean sessions 3.7 (significantly more total activation time), 8 weeks (n=5) |  |
| 3. CBASP studies included in the analysis | | | | | | |
| Authors | Mean age (SD), female gender | Sample | Conditions (n) |  | Length of treatment (n dropout) | Outcome measures |
| Keller et al. 2000  100%chronic | 43 (10.7), 65.3% | DSM-IV criteria for chronic MDD (≥2 years), aged 18-75 years, HRSD-35 score ≥ 20 at screening and baseline | 1. Control: Antidepressant pharmacotherapy (n=220) | Yes | 200-600 mg/d, 12 weeks (n=53) | SCID, HRSD |
|  |  |  | 2. Cognitive behavioral-analysis system of psychotherapy and antidepressant pharmacotherapy (n=226) | Yes | 16-20 sessions, 12 weeks (n=47) |  |
|  |  |  | 3. Cognitive behavioral-analysis system of psychotherapy (n=216) 🡪 2. and 3. were pooled. | No | 16-20 sessions, 12 weeks (n=43) |  |
| Kocsis et al. 2009  100%chronic | 43.2 (13.4), 49% | DSM-IV criteria for MDD (≥4 weeks) and depressive symptoms ≥2 years without remission, criteria for double depression, chronic major depression, or recurrent major depression, HRSD score ≥ 20, aged 18-75 years | 1. Control: Antidepressant pharmacotherapy (n=96) | Yes | 12 weeks (n=16) | SCID, HRSD, QIDS |
|  | 46.4 (11.7), 57.9% |  | 2. Antidepressant pharmacotherapy and brief supportive psychotherapy (n=195) | Yes | 16-20 sessions, 12 weeks (n=27) |  |
|  | 45.3 (11.9), 56% |  | 3. Antidepressant pharmacotherapy and Cogntive behavioral-analysis system of psychotherapy (n=200) | Yes | 16-20 sessions, 12 weeks (n=25) |  |
| Rief et al. 2018  35.8% chronic | 36.10 (11.9), 53.4% | DSM-IV diagnosis of MDD, a BDI score of >14, ability to practice physical exercise | 1. Control: Cognitive behavioral therapy, CBT-E (physical exercises) or CBT-M (mindful sensory perception tasks), cognitive therapy; (n=88) | Yes | 16 sessions, 16 weeks (n=15) | SCID, BDI-II |
|  | 38.8 (13.7), 47.6% |  | 2. Control: Waitlist (n=42) | Yes | 16 weeks (n=5) |  |
|  | 40.4 (13.0), 67.4% |  | 3. Cognitive behavioral-analysis system of psychotherapy (n=43) | Yes | 16 weeks (n=6) |  |
| Schramm et al. 2015  100%chronic | 42.70 (11.58), 53.33% | DSM-IV criteria for current chronic MDD (min 1yr) or recurrent MDE ( ≥ 3 episodes with the preceding  episode no more than 2.5 years before the onset of the current  episode), aged 18-65 years, MADRS score ≥18, medication free >2 weeks before baseline | 1. Control: Antidepressant pharmacotherapy (n=31) | Yes | 18 sessions, 20 min, 28 weeks (n=7) | SCID, MADRS, IDS-SR |
|  | 44.59 (9.51), 55.17% |  | 2. Cognitive behavioral-analysis system of psychotherapy, extended treatment, if neccessary (Escitalopram); (n=29) | No | 22 sessions, 28 weeks (n=2) (data at 8 weeks included in NMA) |  |
| Schramm et al. 2017  100% chronic | 44.9 (11.8), 66% | DSM-IV diagnosis of chronic MDD, double depression, recurrent without remission, aged 18-65 years, HRSD-24 ≥ 20 | 1. Control: Supportive psychotherapy (n=131) | No | >32 sessions, 48 weeks (n=35) | SCID, HRSD-24, IDS-SR |
|  |  |  | 2. Cognitive behavioral-analysis system of psychotherapy (n=137) | No | >32 sessions, 48 weeks (n=24) |  |
| Wiersma et al. 2014  100% chronic | 43.0 (10.1), 51.4% | DSM-IV diagnosis of chronic MDD, aged 18-65 years | 1. Control: Treatment as usual (n=72) including psychotherapy (CBT) | Yes | mean 23 sessions, 45 min, 52 weeks (n=19) | MINI, IDS-SR |
|  | 40.1 (10.8), 68.7% |  | 2. Cognitive behavioral-analysis system of psychotherapy (n=67) | Yes | 24 sessions, 45 min, 52 weeks (n=16) |  |
| 4. MBCT studies included in the analysis | | | | | | |
| Author | Mean age (SD), female gender | Sample | Conditions (n) |  | Length of treatment (n dropout) | Outcome Measures |
| Barnhofer et al. 2009  67.5% chronic depression | 41.79 (9.52), 64.29%, | >3 episodes of major depression/chronic depression, current diagnosis of Major Depression or residual symptoms following a full episode (4 DSM- IV criteria for < 2 weeks or >5 criteria half of the days) 🡪 67.5% chronic depression, aged 18-65 years | 1. Control: Treatment as usual (n= 15) | Yes | 8 weeks (n=2) | SCID, BDI-II |
|  | 42.07 (11.34), 71.43%, |  | 2. Mindfulness-based therapy (n=16) | Yes | 8 (1/w), 120 min, 8 weeks (n=2) |  |
| Chiesa et al. 2015 | 46.70 (10.89), 75% | DSM-IV-TR MDD diagnosis, single or recurrent episode; antidepressants at adequate dosages >8 weeks, failure to achieve remission at screening visit, HRSD-21 score ≥8, aged 18-65 years | 1. Control: Psychoeducation and aerobic activity (30-45 min, 6/w); (n=20) | Yes | 8 sessions, 120 min, 8 weeks (n=4) | MINI, HRSD, BDI-II |
|  | 50.91 (11.48), 70% |  | 2. Mindfulness-based therapy (n=23) | Yes | 8 (1/w), 120 min, 8 weeks (n=3) |  |
| Cladder-Micus et al. 2018  TRD 100% | 47.1 (10.25), 62% | DSM-IV criteria for current MDE ≥12 months, IDS-SR score ≥ 21, adequate trial of antidepressant medication and previous psychological treatment during the current episode ( ≥10 CBT or IPT sessions or <10 sessions if discontinued because of patient's withdrawal) 🡪 TRD, age ≥ 18 | 1. Control: Treatment as usual (n=52) | Yes (82%) | 8 weeks (n=5) | MINI, IDS-SR |
|  |  |  | 2. Mindfulness-based cognitive therapy and treatment as usual, 8-12 patients per group (n=44) | Yes (75%) | 8 sessions, 150 min, 8 weeks (n=5) |  |
| De Jong et al. 2017 | 50.7 (11.4), 75% | Chronic pain ≥3 months, DSM-IV criteria for MDD, Dysthymic Disorder, Depressive Disorder Not Otherwise Specified (NOS), QIDS-C16 score ≥6, stable psychotherapy, analgetics and antidepressant medication (within 8 weeks prior), aged ≥18 years; | 1. Control: Waitlist (n=14) (86% MDD, 14% NOS) | Yes (58%) | 8 weeks (n=0) | SCID, QIDS-C16, HRSD-17 |
|  |  |  | 2. Mindfulness-based therapy (n=19), (85% MDD; 15% NOS) | Yes (44%) | 8 sessions, 120 min, 8 weeks (n=7) |  |
| Eisendrath et al. 2015 | 37.0 (10.8), 65% | DSM-IV-TR criteria for MDD, HRSD-17 score ≥17 | 1. Control: Antidepressant pharmacotherapy (n=20) | Yes | 50-200 mg/d, 8 weeks (n=0) | SCID, HRSD17 |
|  | 35.3 (10.0), 78% |  | 2. Mindfulness-based therapy (adapted); (n=23) | No | 8 sessions, 135 min, 8 weeks (n=0) |  |
| Eisendrath et al. 2016  100% TRD  59% cronic | 45.2 (11.19), 76.7% | DSM-IV criteria for MDD, HRSD-17 score ≥14; antidepressant medications required with two or more adequate trials prescribed during the current episode, aged ≥18 years (TRD) | 1. Control: Treatment as usual: 'Health Enhancement Program' (n=86) | Yes | 8 sessions, 135 min, 8 weeks (n=14) | SCID, HRSD17 |
|  | 47.1 (13.46), 75.9% |  | 2. Mindfulness-based therapy (n=87) | Yes | 8 sessions, 135 min, 8 weeks (n=11) |  |
| Foroughi et al. 2020  100% TRD | 29.8 (10.45) 72.3% | DSM-IV criteria for MDD, lack of response to adequate  doses of two antidepressants for sufficient time (18  weeks); BDI-II ≥ 17; aged 18 -  50 years. | 1. Control: Antidepressants only (n=15) | Yes | 8 weeks (n=6) | SCID, HRSD, BDI-II |
|  |  |  | 2. Mindfulness-based cognitive therapy (n=15) | Yes | 8 sessions, 8 weeks (n=5) |  |
| Hamidian et al. 2013 | na | Diagnostic criteria of dysthymia or double depression (at least 2 years), >18 years | 1. Control: Antidepressant pharmacotherapy (n=22) | Yes | 8 weeks (n=3) | SCID, BDI-II, |
|  | na |  | 2. Mindfulness-based cognitive therapy and antidepressant pharmacotherapy (n=22) | Yes | 8 sessions, 150 min, 8 weeks (n=3) |  |
| Manicavasgar et al. 2011 | 45 (12.94), 65% | DSM-IV criteria for MDD (CIDI-AUTO), BDI-II ≥ 20, no/stable medication intake (preceeding 3 months), ≥ 18 years | 1. Control: Cognitive behavioral group therapy (n=39) | Yes | 8 sessions, 135 min, 8 weeks (n=12) | CIDI-AUTO, BDI-II |
|  | 47 (13.84), 63% |  | 2. Mindfulness-based cognitive therapy and antidepressant pharmacotherapy (n=30) | Yes | 8 sessions, 135 min, 8 weeks (n=4) |  |
| Michalak et al. 2015  100% chronic | 54.0 (13.24), 65.7% | DSM-IV diagnosis of MDE, depressive symptoms ≥ 2 years without remission, chronic major depression (MDE ≥ 2 years), double depression, recurrent major depression | 1. Control: Treatment as usual (n=35) | Yes (53%) | 8 weeks (n=3) | HRSD, BDI-II |
|  | 48.4 (11.5), 58.3% |  | 2. Mindfulness-based cognitive therapy (n=36) | Yes (75%) | 8 sessions, 150 min, 8 weeks (n=8) |  |
|  | 50.2 (10.5), 62.9% |  | 3. Cognitive behavioral-analysis system of psychotherapy in group setting (n=35) | Yes (76%) | 8 sessions, 150 min, 8 weeks (n=10) |  |
| Shapero et al. 2018 | 36.89 (15.83), 67% | HRSD-28 score ≥11, no prior experiences with MBCT (or other systematic mindfulness programs), 18-65 years | 1. Control: Waitlist, treatment as usual (n=18) (86% MDD) | Yes | 8 weeks (n=6) | MINI, BDI-II, HRSD-28 |
|  | 39.77 (10.60), 59% |  | 2. Mindfulness-based cognitive therapy (n=22) (78% MDD) | Yes | 8 sessions, 120 min, 8 weeks (n=6) |  |
| van Aalderen et al. 2012 | 47.7 (11.1), 72% | DSM-IV criteria for three or more depressive episodes, stable antidepressant medication for at least 6 weeks and during study phase | 1. Control: Treatment as usual, not otherwise specified (n=108), participation in MBCT after 3 months of waiting 🡪 Waitlist control | Yes | 8 weeks, excluded from analysis n=5 (missing values) | SCID, HRSD, BDI |
|  | 47.3 (11.5), 70% |  | 2. Mindfulness-based cognitive therapy (n=111) | Yes | 8 weeks, dropout n=9, excluded from analysis n=9 (missing values) |  |
| 5. MCT studies included in the analysis | | | | | | |
| Authors | Mean age (SD), female gender | Sample | Conditions (n) |  | Length of treatment (n dropout) | Outcome measures |
| Ashouri et al. 2013 | 32.48 (7.71), 60.6% | DSM-IV criteria for MDD, no psychological therapies (within 6 months prior), 18-65 years | 1. Control: TAU: Antidepressant medication only (n=13) | Yes | insufficient information | SCID ,BDI-II |
|  |  |  | 2. Control: Cognitive behavioral therapy (n=10) | Yes |  |  |
|  |  |  | 3. Meta-cogntive therapy (n=10) | Yes |  |  |
| Callesen et al. 2020 | 35 (12.5) 69% female | meeting diagnostic DSM-IV-TR criteria for MDD, aged 18–70 years | 1. Control: Cognitive behavioral therapy n=82 | Yes (35% of total sample) | Up to 24 sessions of 60 minutes. Mean delivered 6.7 (SD = 4.7): | SCID, HRSD, BDI-II |
|  |  |  | 2. Metacognitive therapy n=73 | Yes | Up to 24 sessions of 60 minutes. Mean delivered 5.5 (SD = 2.4) |  |
| Hagen et al. 2017 | 35.4 (8.8), 52.6% | DSM-IV criteria for primary depression disorder (single episode or recurrent depression), aged ≥18 years | 1. Control: waitlist (n=17) | No | 10 weeks (n=2) | SCID, SCID-II, HRSD, BDI |
|  | 32.2 (11.7), 65.0% |  | 2. Meta-cognitive therapy (n=20) | No | 10 weeks (n=0) |  |
| Jordan et al. 2014 | 35.0 (13.0), 48% | DSM-IV criteria for MDD, bipolar II or bipolar not-otherwise-specified- depressed, aged 18–65 years | 1. Cognitive behavioral therapy (n=25) | No | 12 sessions, 12 weeks (n=6) | SCID, QIDS-SR, QIDS-C |
|  | 37.2 (12.7), 48% |  | 2. Metacognitive therapy (n=23) | No | 12 sessions, 12 weeks (n=5) |  |
| Zemestani et al. 2015 | 24.2 (), 60.98% | DSM-IV criteria for MDD, BDI-II score >19, students 18-30 years | 1. Control: waitlist (n=15) | No | 8 sessions, 90 min, 8 weeks (n=0) | SCID, BDI-II |
|  |  |  | 2. Behavioral activation (n=15) | No | 8 sessions, 90 min, 8 weeks (n=0) |  |
|  |  |  | 3. Meta-cognitive therapy (n=15) | No | 8 sessions, 90 min, 8 weeks (n=0) |  |
| 6. DBT studies included in the analysis | | | | | | |
| Authors | mean/median age (SD), %female | Inclusion | Conditions (n) |  | Length of treatment (n dropout) | Outcome measures |
| Harley et al. 2008  100% TRD | 41.8 (), 75% | DSM-IV criteria for MDD, treatment-resistant to stable dose of antidepressant, 18-65 years | 1. Control: waitlist/treatment as usual (n=9) (weekly contact, ongoing antidepressant treatment) | Yes | 28 sessions, 120 min, 16 weeks (n=2) | SCID, HRSD, BDI |
|  |  |  | 2. Dialectical behavioral therapy, group format (n=10) | Yes | 16 sessions, 90 min, 16 weeks (n=3) |  |
| Lynch et al. 2003 | 66 (5.0), 85% | Duke Depression Evaluation Schedule criteria for MDD, HRSD score ≥18, BDI score ≥19, antidepressant medication as prescribed by study psychiatrist, ≥ 60 years old | 1. Control: Antiderpressant pharmacotherapy (n=17) | Yes | >2 medication management visits, 28 weeks (n=9) | HRSD, BDI |
|  |  |  | 2. Dialectical behavioral therapy, group format (n=17) | Yes | 28 sessions, 120 min,; 28 telephone coaching sessions, 30 min; 28 weeks (n=5) |  |
| Lynch et al. 2018  100% TRD , 84.3% chronic | 47 (11), 68.5% | DSM-IV criteria for MDD, HRSD score ≥15, refractory or chronic depression, adequate antidepressant medication (at least 6 weeks without response), ≥ 18 years old | 1. Control: Treatment as usual (n=88) | Yes | 3 follow-up visits, (n=16) | SCID, HRSD |
|  |  |  | 2. Dialectical behavioral therapy (focus on radical openness) and treatment as usual, individual and group format (n=162) | Yes | 29 sessions, 60 min; 27 skills classes, 150 min; 29 weeks (n=24). (Data at 7 months included in NMA) |  |
| 7. PP studies included in the analysis | | | | | | |
| Authors | Mean age, (SD), female gender | Sample | Conditions |  | Length of treatment | Outcome measures |
| Ashgaripoor et al. 2012 | 26.88 (8.10), 66.7% | DSM-IV-TR criteria for MDD, at least junior high school degree, no history of psychotherapy, 20-40 years | 1. Control: Cognitive behavioral group therapy (n=9) | Yes | 12 sessions, 120 min, 12 weeks (n=0) | SCID, BDI-II |
|  | 26 (3.64), 77.7% |  | 2. Positive psychotherapy (group) (n=9) | Yes | 12 sessions, 120 min, 12 weeks (n=0) |  |
| Chaves et al. 2016 | 50.43 (10.16), 100% | DSM-  IV diagnosis of major depression or dysthymia, using the  SCID structured interview . 21.9 % dysthymic | 1. Control: cognitive behavioral group therapy (n=49) | Yes (61.2%) | 10 weeks (2h/week) groups of 15 max. | SCID, BDI-II, MADRS |
|  |  |  | 2. Positive psychotherapy (group) (n=47) | Yes (60%) | 10 weeks (2h/week) groups of 15 max. |  |
| Furchtlehner et al. 2019 | 40.66 (12.4), 64.1% | DSM-IV criteria for single episode or recurrent, MDD in partial remission (mild to moderate), dysthymia with or without  double depression and at least normal intelligence, 18-60 years | 1. Control: cognitive behavioral group therapy (n=46) | Yes. No information on percentage of participants taking medication | 14 sessions, 120 min, 14 weeks (n=11) | SCID, BDI-II |
|  |  |  | 2. Positive psychotherapy (group) (n=46) |  | 14 sessions, 120 min, 14 weeks (n=9) |  |
| Seligman et al. 2006 | NA (NA), 75% | DSM-IV-TR criteria for MDD, no ongoing treatment for depression | 1. Control: Treatment as usual (n=15) | No | (n=6) | SCID, HRSD |
|  |  |  | 2. Positive psychotherapy (n=13) | No | 14 sessions, 12 weeks (n=2) |  |
| 8. SCHEMA studies included in the analysis | | | | | | |
| Authors | Mean age, (SD), female gender | Sample | Conditions |  | Length of treatment | Outcome measures |
| Carter et al. 2013  chronic 67% | 38.2 (12.0), 66% | DSM-IV criteria for MDD, centrally active drug prohibited, majority chronic 67% , ≥18 years | 1. Control: Cognitive behavioral therapy (n=50) | No | >18 sessions, 52 weeks (n=25) | SCID, MADRS, BDI-II |
|  | 38.5 (11.4), 72% |  | 2. Schema therapy (n=50) | No | >18 session, 52 weeks (n=20) (data at 6 months included in NMA) |  |

# Table S12: Distributions of studies over comparisons

|  | Comparison | | number of studies | number of patients | number of drop-outs |  |
| --- | --- | --- | --- | --- | --- | --- |
| 1 | ACT vs. ActiveCt | | 1 | 60 | 5 |  |
| 2 | ACT vs. BA | | 1 | 12 | 5 |  |
| 3 | ACT vs. CT_CBT | | 3 | 126 | 16 |  |
| 4 | ACT vs. TAU | | 1 | 34 | 10 |  |
| 5 | ACT vs. WL | | 2 | 158 | 5 |  |
| 6 | ActiveCt vs. BA | | 4 | 393 | 62 |  |
| 7 | ActiveCt vs. CBASP | | 2 | 655 | 78 |  |
| 8 | ActiveCt vs. MBCT | | 3 | 319 | 52 |  |
| 9 | ActiveCt vs. MM | | 1 | 291 | 43 |  |
| 10 | ActiveCt vs. TAU | | 1 | 16 | 0 |  |
| 11 | BA vs. CT_CBT | | 4 | 617 | 93 |  |
| 12 | BA vs. MBCT | | 1 | 81 | 11 |  |
| 13 | BA vs. MCT | | 1 | 30 | 0 |  |
| 14 | BA vs. MM | | 2 | 226 | 54 |  |
| 15 | BA vs. TAU | | 5 | 258 | 71 |  |
| 16 | BA vs. WL | | 4 | 269 | 36 |  |
| 17 | CBASP vs. CT_CBT | | 2 | 270 | 50 |  |
| 18 | CBASP vs. MBCT | | 1 | 70 | 11 |  |
| 19 | CBASP vs. MM | | 3 | 1017 | 190 |  |
| 20 | CBASP vs. TAU | | 1 | 70 | 11 |  |
| 21 | CBASP vs. WL | | 1 | 85 | 11 |  |
| 22 | CT_CBT vs. MBCT | | 1 | 69 | 16 |  |
| 23 | CT_CBT vs. MCT | | 3 | 242 | 73 |  |
| 24 | CT_CBT vs. MM | | 1 | 130 | 35 |  |
| 25 | CT_CBT vs. PP | | 3 | 206 | 40 |  |
| 26 | CT_CBT vs. ST | | 1 | 100 | 25 |  |
| 27 | CT_CBT vs. TAU | | 2 | 75 | 29 |  |
| 28 | CT_CBT vs. WL | | 1 | 130 | 20 |  |
| 29 | DBT vs. TAU | | 2 | 269 | 42 |  |
| 30 | DBT vs. WL | | 1 | 24 | 5 |  |
| 31 | MBCT vs. MM | | 1 | 43 | 7 |  |
| 32 | MBCT vs. TAU | | 4 | 181 | 22 |  |
| 33 | MBCT vs. WL | | 3 | 298 | 38 |  |
| 34 | MCT vs. TAU | | 1 | 23 |  |  |
| 35 | MCT vs. WL | | 2 | 69 | 2 |  |
| 36 | PP vs. TAU | | 1 | 28 | 8 |  |
|  |  | efficacy | | | accepatbility | |
|  |  | studies per condition | patients per condition | | studies per condition | patients per condition |
| 1 | ACT | 8 | 199 | | 7 | 189 |
| 2 | ActiveCt | 10 | 705 | | 10 | 705 |
| 3 | BA | 18 | 865 | | 18 | 865 |
| 4 | CBASP | 7 | 951 | | 7 | 951 |
| 5 | CT_CBT | 17 | 849 | | 14 | 821 |
| 6 | DBT | 3 | 177 | | 3 | 177 |
| 7 | MBCT | 13 | 499 | | 13 | 499 |
| 8 | MCT | 5 | 153 | | 4 | 143 |
| 9 | MM | 6 | 502 | | 6 | 502 |
| 10 | PP | 4 | 115 | | 3 | 106 |
| 11 | ST | 1 | 50 | | 1 | 50 |
| 12 | TAU | 14 | 371 | | 13 | 358 |
| 13 | WL | 12 | 391 | | 12 | 391 |

# Tables S13 and S14: Relative effects tables

## Table S13: Efficacy

| **Cognitive therapy/Cognitive Behavioral Therapy** | | | |
| --- | --- | --- | --- |
| treatment | Posterior Mean | 95% CrI |  |
| ACT | 0.01 | [-0.53; | 0.55] |
| BA | -0.06 | [-0.48; | 0.36] |
| CBASP | -0.29 | [-0.84; | 0.25] |
| DBT | -0.69 | [-1.59; | 0.16] |
| MBCT | -0.33 | [-0.84; | 0.17] |
| MCT | 0.59 | [-0.01; | 1.21] |
| PP | 0.15 | [-0.55; | 0.82] |
| ST | -0.14 | [-1.36; | 1.09] |
|  |  |  |  |
| **Medication Management** | |  |  |
| treatment | Posterior Mean | 95% CrI |  |
| ACT | 0.38 | [-0.34; | 1.11] |
| BA | 0.31 | [-0.24; | 0.88] |
| CBASP | 0.08 | [-0.48; | 0.62] |
| DBT | -0.32 | [-1.31; | 0.64] |
| MBCT | 0.03 | [-0.57; | 0.63] |
| **MCT** | **0.96** | **[0.17;** | **1.78]** |
| PP | 0.51 | [-0.38; | 1.39] |
| ST | 0.23 | [-1.13; | 1.59] |
|  |  |  |  |
| **Active Control`** | | | |
| treatment | Posterior Mean | 95% CrI |  |
| **ACT** | **0.78** | **[0.15;** | **1.43]** |
| **BA** | **0.71** | **[0.26;** | **1.19]** |
| CBASP | 0.48 | [-0.07; | 1.04] |
| DBT | 0.08 | [-0.83; | 0.98] |
| MBCT | 0.43 | [-0.04; | 0.92] |
| **MCT** | **1.36** | **[0.62;** | **2.13]** |
| **PP** | **0.91** | **[0.08;** | **1.76]** |
| ST | 0.63 | [-0.71; | 1.98] |
|  |  |  |  |
| **Treatment as Usual** | | | |
| treatment | Posterior Mean | 95% CrI |  |
| **ACT** | **1.12** | **[0.52;** | **1.74]** |
| **BA** | **1.06** | **[0.63;** | **1.5]** |
| **CBASP** | **0.82** | **[0.24;** | **1.41]** |
| DBT | 0.42 | [-0.37; | 1.19] |
| **MBCT** | **0.78** | **[0.31;** | **1.25]** |
| **MCT** | **1.7** | **[1.02;** | **2.43]** |
| **PP** | **1.25** | **[0.48;** | **2.04]** |
| ST | 0.97 | [-0.33; | 2.3] |
|  |  |  |  |
| **Waitlist** | | | |
| treatment | Posterior Mean | 95% CrI |  |
| **ACT** | **1.39** | **[0.83;** | **2]** |
| **BA** | **1.33** | **[0.87;** | **1.82]** |
| **CBASP** | **1.09** | **[0.51;** | **1.72]** |
| DBT | 0.69 | [-0.16; | 1.57] |
| **MBCT** | **1.05** | **[0.57;** | **1.57]** |
| **MCT** | **1.97** | **[1.31;** | **2.7]** |
| **PP** | **1.53** | **[0.71;** | **2.37]** |
| ST | 1.24 | [-0.05; | 2.61] |

## Table S14: Acceptability

| **Cognitive therapy/Cognitive Behavioral Therapy** | | | |
| --- | --- | --- | --- |
| treatment | Posterior Mean | 95% CrI |  |
| ACT | 0.65 | [0.24; | 1.81] |
| BA | 1.16 | [0.58; | 2.33] |
| CBASP | 1.48 | [0.68; | 3.39] |
| DBT | 1.11 | [0.27; | 4.71] |
| MBCT | 1.05 | [0.48; | 2.36] |
| MCT | 0.77 | [0.24; | 2.32] |
| PP | 0.89 | [0.3; | 2.58] |
| ST | 0.72 | [0.14; | 3.85] |
|  |  |  |  |
| **Medication Management** | | | |
| treatment | Posterior Mean | 95% CrI |  |
| **ACT** | **0.27** | **[0.08;** | **0.88]** |
| BA | 0.48 | [0.21; | 1.07] |
| CBASP | 0.61 | [0.28; | 1.33] |
| DBT | 0.46 | [0.1; | 2.07] |
| MBCT | 0.43 | [0.18; | 1.06] |
| MCT | 0.32 | [0.07; | 1.25] |
| PP | 0.36 | [0.09; | 1.36] |
| ST | 0.3 | [0.04; | 1.93] |
|  |  |  |  |
| **Active Control** | | | |
| treatment | Posterior Mean | 95% CrI |  |
| ACT | 0.69 | [0.23; | 2.12] |
| BA | 1.23 | [0.58; | 2.61] |
| CBASP | 1.57 | [0.72; | 3.54] |
| DBT | 1.18 | [0.27; | 5.17] |
| MBCT | 1.12 | [0.53; | 2.36] |
| MCT | 0.81 | [0.2; | 3.18] |
| PP | 0.94 | [0.24; | 3.46] |
| ST | 0.77 | [0.11; | 4.93] |
|  |  |  |  |
| **Treatment as Usual** | | | |
| treatment | Posterior Mean | 95% CrI |  |
| ACT | 0.52 | [0.17; | 1.53] |
| BA | 0.92 | [0.45; | 1.9] |
| CBASP | 1.17 | [0.48; | 3.03] |
| DBT | 0.88 | [0.25; | 3.15] |
| MBCT | 0.84 | [0.38; | 1.87] |
| MCT | 0.61 | [0.15; | 2.37] |
| PP | 0.7 | [0.2; | 2.38] |
| ST | 0.57 | [0.09; | 3.75] |
|  |  |  |  |
| **Waitlist** | | | |
| treatment | Posterior Mean | 95% CrI |  |
| ACT | 0.9 | [0.29; | 2.69] |
| BA | 1.58 | [0.71; | 3.48] |
| CBASP | 2.03 | [0.8; | 5.26] |
| DBT | 1.54 | [0.37; | 6.28] |
| MBCT | 1.43 | [0.65; | 3.21] |
| **MCT** | 1.06 | [0.25; | 3.91] |
| PP | 1.2 | [0.31; | 4.47] |
| ST | 0.98 | [0.14; | 6.17] |

# Table S15: Results of pairwise meta-analyses - Efficacy data

| ACT vs. ActiveCt |
| --- |
|  |
| ACT vs. BA |
|  |
| ACT vs. CT_CBT |
|  |
| Egger’s Test : intercept= 0.83 (95% CI: -6.6; 8.24), t= 0.22, p=0.87 |
| ACT vs. TAU |
|  |
| \| ACT vs. WL \| \| --- \| |
|  |
| ActiveCt vs. MM |
|  |
| ActiveCt vs. TAU |
|  |
| BA vs. ActiveCt |
|  |
| Egger’s Test : intercept= 0.14 (95% CI: -1.09; 1.37), t= 0.22, p=0.85 |
| BA vs. CT_CBT |
|  |
| Egger’s Test : intercept= 0.18 (95% CI: -1.46; 1.82), t= 0.22, p=0.85 |
| BA vs. MBCT |
|  |
| BA vs. MCT |
|  |
| BA vs. MM |
|  |
| BA vs. TAU |
|  |
| Egger’s Test : intercept= 1.1 (95% CI: -4.55; 6.7), t= 0.37, p=0.73 |
| BA vs. WL |
|  |
| Egger’s Test : intercept= 5.4 (95% CI: 2.4; 8.36), t= 3.54, p=0.07 |
| CBASP vs. ActiveCt |
|  |
| CBASP vs. CT_CBT |
|  |
| CBASP vs. MBCT |
|  |
| CBASP vs. MM |
|  |
| Egger’s Test : intercept= -3.3 (95% CI: -3.68; -2.83), t= -15.04, p=0.042 |
| CBASP vs. TAU |
|  |
| CBASP vs. WL |
|  |
| CT_CBT vs. MM |
|  |
| CT_CBT vs. TAU |
|  |
| CT_CBT vs. WL |
|  |
| DBT vs. TAU |
|  |
| DBT vs. WL |
|  |
| MBCT vs. ActiveCt |
|  |
| Egger’s Test : intercept= 0.8 (95%CI: 0.39; 1.21) t=3.81, p= 0.16 |
| MBCT vs. CT_CBT |
|  |
| MBCT vs. MM |
|  |
| MBCT vs. TAU |
|  |
| Egger’s Test : intercept= 5.6 (95%CI: 4.53; 6.76) t=9.92, p= 0.01 |
| MBCT vs. WL |
|  |
| Egger’s Test : intercept=2.17 (95%CI: -12.46; 16.79) t=0.29, p= 0.82 |
| MCT vs. CT_CBT |
|  |
| Egger’s Test : intercept=1.02 (95%CI: -2.07; -4.12) t=0.65, p= 0.63 |
| MCT vs. TAU |
|  |
| MCT vs. WL |
|  |
| PP vs. CT_CBT |
|  |
| PP vs. CT_CBT |
|  |
| Egger’s Test : intercept= -3.05 (95%CI: -13.58; 7.48) t=-0.57, p= 0.67 |
| PP vs. TAU |
|  |
| ST vs. CT_CBT |
|  |

# Table S9: Results of pairwise meta-analyses - Acceptability data

| ACT vs. ActiveCt |
| --- |
|  |
| ACT vs. BA |
|  |
| ACT vs. CT_CBT |
|  |
| ACT vs. TAU |
|  |
| \| ACT vs. WL \| \| --- \| |
|  |
| ActiveCt vs. MM |
|  |
| ActiveCt vs. TAU |
|  |
| BA vs. ActiveCt |
|  |
| BA vs. CT_CBT |
|  |
| BA vs. MBCT |
|  |
| BA vs. MCT |
|  |
| BA vs. MM |
|  |
| BA vs. TAU |
|  |
| BA vs. WL |
|  |
| CBASP vs. ActiveCt |
|  |
| CBASP vs. CT_CBT |
|  |
| CBASP vs. MBCT |
|  |
| CBASP vs. MM |
|  |
| CBASP vs. TAU |
|  |
| CBASP vs. WL |
|  |
| CT_CBT vs. MM |
|  |
| CT_CBT vs. TAU |
|  |
| CT_CBT vs. WL |
|  |
| DBT vs. TAU |
|  |
| DBT vs. WL |
|  |
| MBCT vs. ActiveCt |
|  |
| MBCT vs. CT_CBT |
|  |
| MBCT vs. MM |
|  |
| MBCT vs. TAU |
|  |
| MBCT vs. WL |
|  |
| MCT vs. CT_CBT |
|  |
| MCT vs. TAU |
|  |
| MCT vs. WL |
|  |
| PP vs. CT_CBT |
|  |
| PP vs. TAU |
|  |
| ST vs. CT_CBT |
|  |

## Text S16: Summary of random effects pairwise meta–analyses

All third–wave treatments were more efficacious than WL. None of the third–wave treatments was more efficacious than CT/CBT. Heterogeneity was high (*I^2^* > 50%) in 7 out of 18 pairwise efficacy comparisons fit for pooling data (MCT vs. WL, MBCT vs. TAU, BA vs. WL, CBASP vs. CT/CBT, CBASP vs. MM, ACT vs. CT/CBT, and PP vs. CT/CBT). For acceptability, CBASP had higher discontinuation rates than did TAU and MBCT (each k = 1). and BA had significantly lower discontinuation rates than did MM (k = 2). None of the remaining acceptability comparisons showed significant superiority of one condition over another. Four out of 18 pooled effect sizes showed high heterogeneity between trials (*I^2^* > 50%).

# Table S17: Model comparison consistency vs. inconsistency models

| **1. Efficacy** |  |  |
| --- | --- | --- |
|  | Consistency Model | Inconsistency Model |
| Residual deviance | 81.65 | 88.64 |
| Parameters | 51.48 | 57.93 |
| DIC | 133.13 | 146.56 |
| data points | 63.00 | 71.00 |
|  |  |  |
| **2. Acceptability** |  |  |
|  | Consistency Model | Inconsistency Model |
| Residual deviance | 107.28 | 109.95 |
| Parameters | 81.60 | 87.62 |
| DIC | 188.88 | 197.57 |
| data points | 111.00 | 111.00 |

# Figure S18: Leverage plots

**A**

**B**

**Figure S18. Leverage plots for efficacy data (A) and acceptability data (B).** Leverage plots depict each studies’ contribution to the overall deviance against (x-axis) the leverage (the contribution of each individual observation to its own fitted value, y-axis).

High deviance studies (individual deviance >2) in A are Zemestani et al. (2020), Zemestani et al. (2016) and Foroughi et al. (2020).

# Tables S19 and S20: Subgroup and sensitivity analyses

All coefficients reported are relative to CT/CBT

**Table S19: Efficacy data**

Heterogeneity parameters are given as standard deviations (tau).

Heterogeneity estimate tau for full network: 0.57 (95% CrI 0.40–0.79)

| **Effects** | **Covariate: Baseline severity. range 1 (mild) to 4 (severe). Effect estimates at covariate centering value = 3.0418** | | | **Covariate: Risk of bias dichotomized high vs. low (effect estimates for covariate value = 0 = low risk of bias)** | | | **Covariate: Manualized control group (effect estimated for covariate value = 1 = manualized)** | | | **Excluding trials with medication management controls (k=49 studies)** | |
| --- | --- | --- | --- | --- | --- | --- | --- | --- | --- | --- | --- |
| **treatment** | **Posterior Mean** | **95% CrI** |  | **Posterior Mean** | **95% CrI** |  | **Posterior Mean** | **95% CrI** |  | **Posterior Mean** | **95% CrI** |
| **ACT** | 0.01 | [-0.56; | 0.57] | -0.02 | [-0.71; | 0.67] | 0.01 | [-0.55; | 0.58] | -0.03 | [-0.65; 0.59] |
| **BA** | -0.06 | [-0.52; | 0.38] | -0.09 | [-0.7; | 0.51] | -0.06 | [-0.51; | 0.39] | -0.13 | [-0.66;  0.39] |
| **CBASP** | -0.29 | [-0.87; | 0.25] | -0.32 | [-0.97; | 0.31] | -0.29 | [-0.9; | 0.3] | -0.27 | [-1;  0.44] |
| **DBT** | -0.70 | [-1.62; | 0.19] | -0.73 | [-1.75; | 0.26] | -0.69 | [-1.61; | 0.21] | -0.77 | [-1.79; 0.22] |
| **MBCT** | -0.34 | [-0.88; | 0.17] | -0.37 | [-1.05; | 0.29] | -0.34 | [-0.87; | 0.2] | -0.42 | [-1.03; 0.17] |
| **MCT** | 0.59 | [-0.01; | 1.23] | 0.57 | [-0.09; | 1.27] | 0.59 | [-0.02; | 1.25] | 0.59 | [-0.08; 1.3] |
| **PP** | 0.13 | [-0.59; | 0.84] | 0.09 | [-0.87; | 1.03] | 0.15 | [-0.58; | 0.86] | 0.12 | [-0.66; 0.88] |
| **ST** | -0.13 | [-1.38; | 1.11] | -0.20 | [-1.63; | 1.24] | -0.14 | [-1.38; | 1.11] | -0.13 | [-1.56; 1.27] |
| **sd** (tau) | 0.59 | [0.41; | 0.81] | 0.59 | [0.41; | 0.81] | 0.58 | [0.41; | 0.81] | 0.67 | [0.47; 0.93] |
| **B coefficient** | 0.04 | [-0.75; | 0.83] | 0.06 | [-0.66; | 0.76] | 0.01 | [-0.84; | 0.88] | - | - |
| **% change from full NMA tau** | +3.5% |  |  | +3.5% |  |  | +1.75% |  |  | +17% |  |
| **Residdual deviance** | 81.01 |  |  | 80.76 |  |  | 81.05 |  |  | 68.35 |  |
| **Parametera** | 52.00 |  |  | 51.70 |  |  | 52.08 |  |  | 46.25 |  |
| **DIC** | 133.01 |  |  | 132.46 |  |  | 133.13 |  |  | 114.61 |  |
| **Data points** | 63.00 |  |  | 63.00 |  |  | 63.00 |  |  | 55 |  |

|  | **Including only treatment resistant/chronically depressed patients (k=16 studies)** | | |  | **Excluding trials with waitlist controls (k=44 studies)** | | | **Excluding all trials with comorbidities (k=51 studies)** | | | **Excluding high deviance studies (resid. deviance >2) (k=52 studies)** | | |
| --- | --- | --- | --- | --- | --- | --- | --- | --- | --- | --- | --- | --- | --- |
| **treatment** | **Posterior Mean** | **95% CrI** | | **treatment** | **Posterior Mean** | **95% CrI** | | **Posterior Mean** | **95% CrI** | | **Posterior Mean** | **95% CrI** |  |
| **ACT** | 1.82 | [-0.28; | 3.9] | **ACT** | 0.26 | [-0.24; | 0.76] | -0.19 | [-0.72; | 0.33] | -0.16 | [-0.54; | 0.21] |
| **ActiveCt** | -0.06 | [-0.77; | 0.69] | **BA** | -0.15 | [-0.54; | 0.21] | 0.06 | [-0.3; | 0.41] | 0.00 | [-0.28; | 0.27] |
| **BA** | 0.20 | [-0.66; | 1.09] | **CBASP** | -0.16 | [-0.66; | 0.3] | -0.14 | [-0.6; | 0.29] | -0.12 | [-0.46; | 0.19] |
| **CBASP** | 0.17 | [-0.38; | 0.72] | **DBT** | -0.73 | [-1.55; | 0.05] | -0.62 | [-1.38; | 0.1] | -0.47 | [-1.04; | 0.08] |
| **DBT** | -0.05 | [-1.09; | 0.94] | **MBCT** | -0.16 | [-0.62; | 0.27] | -0.14 | [-0.57; | 0.29] | -0.21 | [-0.54; | 0.1] |
| **MBCT** | 0.31 | [-0.43; | 1.16] | **MCT** | 0.25 | [-0.31; | 0.84] | 0.54 | [0.05; | 1.07] | 0.41 | [0; | 0.85] |
| **MM** | 0.08 | [-0.5; | 0.72] | **PP** | 0.18 | [-0.38; | 0.71] | 0.18 | [-0.39; | 0.73] | 0.22 | [-0.22; | 0.65] |
| **ST** | -0.13 | [-1.07; | 0.8] | **ST** | -0.13 | [-1.07; | 0.79] | -0.13 | [-1.12; | 0.86] | -0.13 | [-0.85; | 0.57] |
| **TAU** | -0.62 | [-1.56; | 0.16] | **sd** (tau) | 0.41 | [0.26; | 0.61] | 0.44 | [0.27; | 0.66] | 0.28 | [0.16; | 0.43] |
| **WL** | -0.71 | [-1.55; | 0.06] |  |  |  |  |  |  |  |  |  |  |
| **sd** (tau) | 0.34 | [0.08; | 0.84] |  |  |  |  |  |  |  |  |  |  |
| **% change from full NMA tau** | -40.35% |  |  |  | -28% |  |  | -22.81% |  |  | -50.88% |  |  |
| **Residdual deviance** | 26.32 |  |  |  | 58.21 |  |  | 84.13 |  |  | 69.87 |  |  |
| **Parametera** | 17.69 |  |  |  | 36.68 |  |  | 44.45 |  |  | 36.70 |  |  |
| **DIC** | 44.01 |  |  |  | 94.90 |  |  | 128.58 |  |  | 106.57 |  |  |
| **Data points** | 20.00 |  |  |  | 49.00 |  |  | 59.00 |  |  | 59.00 |  |  |

##

## Table S20: Acceptability data

Heterogeneity parameters are given as standard deviations (tau).

Heterogeneity estimate tau for full network: 0.67 (95% CrI 0.39–1.03)

| **Effects** | **Covariate: Baseline severity. range 1 (mild) to 4 (severe). Effect estimates at covariate centering value = 3.0418** | | | **Covariate: Risk of bias dichotomized high vs. low (effect estimates for covariate value = 0 = low risk of bias)** | | | **Covariate: Manualized control group (effect estimated for covariate value = 1 = manualized)** | | | **Excluding trials with medication management controls (k=46 studies)** | |
| --- | --- | --- | --- | --- | --- | --- | --- | --- | --- | --- | --- |
| **treatment** | **Posterior Mean** | **95% CrI** |  | **Posterior Mean** | **95% CrI** |  | **Posterior Mean** | **95% CrI** |  | **Posterior Mean** | **95% CrI** |
| **ACT** | 0.61 | [0.22; | 1.71] | 0.69 | [0.22; | 2.15] | 0.67 | [0.24; | 1.89] | 0.62 | [0.21; 1.82] |
| **BA** | 1.18 | [0.59; | 2.39] | 1.23 | [0.5; | 3.02] | 1.21 | [0.59; | 2.5] | 1.22 | [0.55; 2.77] |
| **CBASP** | 1.51 | [0.69; | 3.54] | 1.55 | [0.63; | 4.04] | 1.64 | [0.68; | 4.15] | 1.43 | [0.55; 3.95] |
| **DBT** | 1.14 | [0.27; | 4.77] | 1.19 | [0.25; | 5.77] | 1.17 | [0.27; | 4.94] | 1.08 | [0.24; 5] |
| **MBCT** | 1.09 | [0.49; | 2.47] | 1.12 | [0.42; | 3.12] | 1.11 | [0.5; | 2.54] | 0.90 | [0.38; 2.24] |
| **MCT** | 0.66 | [0.18; | 2.33] | 0.77 | [0.23; | 2.33] | 0.77 | [0.23; | 2.38] | 0.74 | [0.21; 2.43] |
| **PP** | 0.99 | [0.3; | 3.1] | 0.97 | [0.21; | 4.27] | 0.88 | [0.29; | 2.63] | 0.86 | [0.26; 2.73] |
| **ST** | 0.72 | [0.13; | 3.95] | 0.80 | [0.1; | 6.28] | 0.72 | [0.13; | 3.9] | 0.72 | [0.12; 4.36] |
| **sd (tau).** | 0.68 | [0.4; | 1.06] | 0.69 | [0.41; | 1.07] | 0.69 | [0.41; | 1.06] | 0.73 | [0.39; 1.18] |
| **B coefficient** | -0.41 | [-1.88; | 1.03] | -0.11 | [-1.24; | 1.02] | 0.38 | [-1.28; | 2.11] | - | - |
| % change from full NMA tau | +1.77% |  |  | +2.8% |  |  | +2.8% |  |  | -0.09% |  |
| **Residdual deviance** | 107.32 |  |  | 107.08 |  |  | 106.82 |  |  | 93.36 |  |
| **Parametera** | 82.21 |  |  | 82.26 |  |  | 82.09 |  |  | 71.54 |  |
| **DIC** | 189.53 |  |  | 189.34 |  |  | 188.92 |  |  | 164.9 |  |
| **Data points** | 111.00 |  |  | 111.00 |  |  | 111.00 |  |  | 97 |  |

|  | **Including only treatment resistant/chronically depressed patients (k=16 studies)** | | |  | **Excluding trials with waitlist controls (k=40 studies)** | | | **Excluding all trials with comorbidities (k=49 studies)** | | |
| --- | --- | --- | --- | --- | --- | --- | --- | --- | --- | --- |
| **treatment** | **Posterior Mean** | **95% CrI** | | **treatment** | **Posterior Mean** | **95% CrI** | | **Posterior Mean** | **95% CrI** | |
| **ACT** | 0.24 | [0.01; | 9.8] | **ACT** | 0.81 | [0.25; | 2.5] | 0.48 | [0.16; | 1.39] |
| **ActiveCt** | 1.01 | [0.17; | 4.79] | **BA** | 1.08 | [0.49; | 2.39] | 1.26 | [0.63; | 2.57] |
| **BA** | 0.53 | [0.06; | 4.55] | **CBASP** | 1.61 | [0.63; | 4.4] | 1.59 | [0.73; | 3.72] |
| **CBASP** | 1.33 | [0.38; | 4.78] | **DBT** | 1.07 | [0.18; | 6.15] | 1.07 | [0.26; | 4.49] |
| **DBT** | 0.53 | [0.05; | 5.16] | **MBCT** | 0.89 | [0.35; | 2.28] | 1.07 | [0.48; | 2.41] |
| **MBCT** | 0.77 | [0.11; | 4.24] | **MCT** | 1.06 | [0.29; | 3.89] | 0.78 | [0.25; | 2.36] |
| **MM** | 2.10 | [0.55; | 8.89] | **PP** | 0.86 | [0.26; | 2.68] | 0.87 | [0.29; | 2.52] |
| **ST** | 0.72 | [0.09; | 5.71] | **ST** | 0.72 | [0.12; | 4.38] | 0.71 | [0.14; | 3.79] |
| **TAU** | 0.49 | [0.06; | 3.19] | **sd** (tau) | 0.73 | [0.43; | 1.15] | 0.67 | [0.4; | 1.04] |
| **WL** | 0.63 | [0.09; | 3.96] |  |  |  |  |  |  |  |
| **sd** (tau) | 0.79 | [0.19; | 1.71] |  |  |  |  |  |  |  |
| **% change from full NMA tau** | +17.7% |  |  |  | +9.55% |  |  | +0% |  |  |
| **Residdual deviance** | 37.31 |  |  |  | 80.49 |  |  | 100.03 |  |  |
| **Parametera** | 32.95 |  |  |  | 66.36 |  |  | 77.33 |  |  |
| **DIC** | 70.26 |  |  |  | 146.85 |  |  | 177.36 |  |  |
| **Data points** | 36.00 |  |  |  | 85.00 |  |  | 105.00 |  |  |

#

# Table S21: Table of node-splitting analysis for efficacy data

| Analysis of inconsistency | | | | |
| --- | --- | --- | --- | --- |
| Efficacy data | | | | |
|  | | | | |
| **Comparison** |  | **95% CrI** |  | **p.value** |
| ACT vs. ActiveCt |  |  |  | 0.00045 |
| direct | -2.7 | (-3.8; | -1.5) |  |
| indirect | -0.26 | (-0.87; | 0.33) |  |
| network | -0.78 | (-1.4; | -0.16) |  |
| ACT vs. BA |  |  |  | 0.1267 |
| direct | -1.6 | (-3.7; | 0.46) |  |
| indirect | 0.056 | (-0.54; | 0.66) |  |
| network | -0.068 | (-0.65; | 0.50) |  |
| ACT vs. CT_CBT |  |  |  | 0.48535 |
| direct | 0.19 | (-0.61; | 0.98) |  |
| indirect | -0.2 | (-0.96; | 0.57) |  |
| network | -0.012 | (-0.56; | 0.54) |  |
| ACT vs. TAU |  |  |  | 0.45955 |
| direct | -0.68 | (-2.0; | 0.70) |  |
| indirect | -1.2 | (-1.9; | -0.56) |  |
| network | -1.1 | (-1.7; | -0.53) |  |
| ACT vs. WL |  |  |  | 0.07055 |
| direct | -0.78 | (-1.7; | 0.099) |  |
| indirect | -1.8 | (-2.6; | -1.1) |  |
| network | -1.4 | (-2.0; | -0.83) |  |
| ActiveCt vs. BA |  |  |  | 0.23655 |
| direct | 0.41 | (-0.26; | 1.1) |  |
| indirect | 0.96 | (0.32; | 1.6) |  |
| network | 0.71 | (0.26; | 1.2) |  |
| ActiveCt vs. CBASP | |  |  | 0.38955 |
| direct | 0.22 | (-0.65; | 1.1) |  |
| indirect | 0.7 | (-0.054; | 1.5) |  |
| network | 0.48 | (-0.075; | 1.0) |  |
| ActiveCt vs. MBCT | |  |  | 0.94345 |
| direct | 0.46 | (-0.27; | 1.2) |  |
| indirect | 0.42 | (-0.24; | 1.1) |  |
| network | 0.44 | (-0.045; | 0.93) |  |
| ActiveCt vs. MM |  |  |  | 0.5076 |
| direct | 0.06 | (-1.2; | 1.3) |  |
| indirect | 0.52 | (-0.21; | 1.3) |  |
| network | 0.4 | (-0.20; | 1.0) |  |
| ActiveCt vs. TAU |  |  |  | 0.63525 |
| direct | -0.0000057 | (-1.5; | 1.5) |  |
| indirect | -0.39 | (-0.98; | 0.20) |  |
| network | -0.34 | (-0.88; | 0.19) |  |
| BA vs. CT_CBT |  |  |  | 0.6655 |
| direct | -0.041 | (-0.69; | 0.60) |  |
| indirect | 0.15 | (-0.45; | 0.77) |  |
| network | 0.054 | (-0.37; | 0.48) |  |
| BA vs. MBCT |  |  |  | 0.95895 |
| direct | -0.25 | (-1.5; | 1.0) |  |
| indirect | -0.29 | (-0.79; | 0.22) |  |
| network | -0.28 | (-0.73; | 0.18) |  |
| BA vs. MCT |  |  |  | 0.16595 |
| direct | -0.17 | (-1.1; | 0.72) |  |
| indirect | 0.57 | (-0.018; | 1.2) |  |
| network | 0.64 | (0.0071; | 1.3) |  |
| BA vs. MM |  |  |  | 0.8912 |
| direct | -0.3 | (-1.2; | 0.60) |  |
| indirect | -0.38 | (-1.2; | 0.40) |  |
| network | -0.31 | (-0.88; | 0.25) |  |
| BA vs. TAU |  |  |  | 0.2043 |
| direct | -0.79 | (-1.4; | -0.20) |  |
| indirect | -1.4 | (-2.0; | -0.70) |  |
| network | -1.1 | (-1.5; | -0.63) |  |
| BA vs. WL |  |  |  | 0.00105 |
| direct | -2.2 | (-3.0; | -1.5) |  |
| indirect | -0.72 | (-1.3; | -0.12) |  |
| network | -1.3 | (-1.8; | -0.87) |  |
| CBASP vs. CT_CBT | |  |  | 0.22785 |
| direct | -0.12 | (-0.98; | 0.72) |  |
| indirect | 0.53 | (-0.17; | 1.3) |  |
| network | 0.29 | (-0.25; | 0.85) |  |
| CBASP vs. MBCT |  |  |  | 0.6127 |
| direct | -0.35 | (-1.6; | 0.94) |  |
| indirect | 0.015 | (-0.64; | 0.68) |  |
| network | -0.045 | (-0.60; | 0.52) |  |
| CBASP vs. MM |  |  |  | 0.87335 |
| direct | -0.036 | (-0.75; | 0.68) |  |
| indirect | -0.13 | (-1.1; | 0.81) |  |
| network | -0.08 | (-0.63; | 0.47) |  |
| CBASP vs. TAU |  |  |  | 0.8606 |
| direct | -0.71 | (-1.9; | 0.55) |  |
| indirect | -0.83 | (-1.5; | -0.15) |  |
| network | -0.82 | (-1.4; | -0.24) |  |
| CBASP vs. WL |  |  |  | 0.29695 |
| direct | -0.52 | (-1.8; | 0.75) |  |
| indirect | -1.3 | (-2.0; | -0.57) |  |
| network | -1.1 | (-1.7; | -0.50) |  |
| CT_CBT vs. MBCT |  |  |  | 0.40945 |
| direct | 0.16 | (-1.2; | 1.5) |  |
| indirect | -0.42 | (-0.97; | 0.12) |  |
| network | -0.33 | (-0.84; | 0.17) |  |
| CT_CBT vs. MCT |  |  |  | 0.0774 |
| direct | 0.14 | (-0.57; | 0.87) |  |
| indirect | 1.3 | (0.26; | 2.3) |  |
| network | 0.59 | (-0.0027; | 1.2) |  |
| CT_CBT vs. MM |  |  |  | 0.4169 |
| direct | 0.068 | (-1.2; | 1.3) |  |
| indirect | -0.51 | (-1.2; | 0.19) |  |
| network | -0.37 | (-0.98; | 0.22) |  |
| CT_CBT vs. PP |  |  |  | 0.76355 |
| direct | 0.1 | (-0.67; | 0.85) |  |
| indirect | 0.37 | (-1.2; | 2.0) |  |
| network | 0.15 | (-0.54; | 0.82) |  |
| CT_CBT vs. TAU |  |  |  | 0.2864 |
| direct | -1.6 | (-2.6; | -0.58) |  |
| indirect | -0.95 | (-1.5; | -0.40) |  |
| network | -1.1 | (-1.6; | -0.64) |  |
| CT_CBT vs. WL |  |  |  | 0.2944 |
| direct | -0.81 | (-2.1; | 0.45) |  |
| indirect | -1.5 | (-2.1; | -0.97) |  |
| network | -1.4 | (-1.9; | -0.89) |  |
| DBT vs vs. TAU |  |  |  | 0.62115 |
| direct | -0.32 | (-1.2; | 0.60) |  |
| indirect | -0.78 | (-2.4; | 0.84) |  |
| network | -0.42 | (-1.2; | 0.36) |  |
| DBT vs. WL |  |  |  | 0.6181 |
| direct | -1 | (-2.5; | 0.53) |  |
| indirect | -0.55 | (-1.6; | 0.51) |  |
| network | -0.69 | (-1.6; | 0.15) |  |
| MBCT vs. MM |  |  |  | 0.71695 |
| direct | -0.25 | (-1.6; | 1.1) |  |
| indirect | 0.017 | (-0.67; | 0.70) |  |
| network | -0.038 | (-0.63; | 0.56) |  |
| MBCT vs. TAU |  |  |  | 0.34745 |
| direct | -1 | (-1.8; | -0.32) |  |
| indirect | -0.57 | (-1.2; | 0.11) |  |
| network | -0.78 | (-1.3; | -0.31) |  |
| MBCT vs. WL |  |  |  | 0.0665 |
| direct | -0.53 | (-1.3; | 0.21) |  |
| indirect | -1.4 | (-2.1; | -0.80) |  |
| network | -1 | (-1.6; | -0.57) |  |
| MCT vs. TAU |  |  |  | 0.1665 |
| direct | -2.6 | (-4.0; | -1.2) |  |
| indirect | -1.5 | (-2.3; | -0.75) |  |
| network | -1.7 | (-2.4; | -1.0) |  |
| MCT vs. WL |  |  |  | 0.00015 |
| direct | -3.6 | (-4.6; | -2.7) |  |
| indirect | -1.3 | (-2.0; | -0.54) |  |
| network | -2 | (-2.7; | -1.3) |  |
| PP vs. TAU |  |  |  | 0.76665 |
| direct | -1.5 | (-3.0; | 0.078) |  |
| indirect | -1.2 | (-2.1; | -0.28) |  |
| network | -1.3 | (-2.0; | -0.49) |  |

# Table S22: Table of node-splitting analysis for acceptability data

| Analysis of  inconsistency | | | | |
| --- | --- | --- | --- | --- |
| Acceptability data | | | | |
|  |  |  |  |  |
| **Comparison** |  | **95% CrI** |  | **p.value** |
| ACT vs. ActiveCt |  |  |  | 0.0682 |
| direct | -1.80 | (-5.4; | 0.77) |  |
| indirect | 0.86 | (-0.36; | 2.1) |  |
| network | 0.37 | (-0.73; | 1.5) |  |
| ACT vs. BA |  |  |  | 0.85875 |
| direct | 0.81 | (-2.0; | 3.8) |  |
| indirect | 0.53 | (-0.59; | 1.7) |  |
| network | 0.57 | (-0.45; | 1.6) |  |
| ACT vs. CT_CBT |  |  |  | 0.8652 |
| direct | 0.52 | (-1.0; | 2.1) |  |
| indirect | 0.35 | (-1.0; | 1.7) |  |
| network | 0.43 | (-0.57; | 1.4) |  |
| ACT vs. TAU |  |  |  | 0.89095 |
| direct | 0.79 | (-1.3; | 2.9) |  |
| indirect | 0.61 | (-0.66; | 1.9) |  |
| network | 0.66 | (-0.41; | 1.7) |  |
| ACT vs. WL |  |  |  | 0.20235 |
| direct | 1.60 | (-0.90; | 4.9) |  |
| indirect | -0.25 | (-1.5; | 1.0) |  |
| network | 0.11 | (-0.99; | 1.2) |  |
| ActiveCt vs. BA |  |  |  | 0.2783 |
| direct | 0.73 | (-0.51; | 1.8) |  |
| indirect | -0.12 | (-1.0; | 0.85) |  |
| network | 0.21 | (-0.55; | 0.95) |  |
| ActiveCt vs. CBASP | |  |  | 0.05805 |
| direct | -0.19 | (-1.2; | 0.84) |  |
| indirect | 1.20 | (0.15; | 2.3) |  |
| network | 0.45 | (-0.34; | 1.3) |  |
| ActiveCt vs. MBCT | |  |  | 0.94465 |
| direct | 0.14 | (-0.93; | 1.2) |  |
| indirect | 0.08 | (-0.99; | 1.2) |  |
| network | 0.11 | (-0.63; | 0.85) |  |
| ActiveCt vs. MM |  |  |  | 0.24385 |
| direct | 0.21 | (-1.4; | 1.8) |  |
| indirect | 1.30 | (0.23; | 2.4) |  |
| network | 0.95 | (0.076; | 1.8) |  |
| ActiveCt vs. TAU |  |  |  | 0.6424 |
| direct | -12.00 | (-64.; | 36.) |  |
| indirect | 0.30 | (-0.60; | 1.2) |  |
| network | 0.30 | (-0.61; | 1.2) |  |
| BA vs. CT_CBT |  |  |  | 0.8185 |
| direct | -0.18 | (-1.2; | 0.80) |  |
| indirect | -0.03 | (-1.1; | 0.98) |  |
| network | -0.15 | (-0.85; | 0.54) |  |
| BA vs. MBCT |  |  |  | 0.84645 |
| direct | -0.26 | (-2.2; | 1.7) |  |
| indirect | -0.06 | (-0.85; | 0.74) |  |
| network | -0.09 | (-0.81; | 0.63) |  |
| BA vs. MCT |  |  |  | 0.77605 |
| direct | -7.70 | (-68.; | 45.) |  |
| indirect | -0.41 | (-1.7; | 0.88) |  |
| network | -0.41 | (-1.7; | 0.87) |  |
| BA vs. MM |  |  |  | 0.07325 |
| direct | 1.50 | (0.32; | 2.8) |  |
| indirect | 0.05 | (-1.0; | 1.2) |  |
| network | 0.74 | (-0.061; | 1.6) |  |
| BA vs. TAU |  |  |  | 0.375 |
| direct | 0.36 | (-0.61; | 1.3) |  |
| indirect | -0.29 | (-1.4; | 0.83) |  |
| network | 0.09 | (-0.63; | 0.78) |  |
| BA vs. WL |  |  |  | 0.6495 |
| direct | -0.70 | (-2.0; | 0.61) |  |
| indirect | -0.32 | (-1.3; | 0.73) |  |
| network | -0.46 | (-1.2; | 0.34) |  |
| CBASP vs. CT_CBT | |  |  | 0.23105 |
| direct | 0.11 | (-1.1; | 1.4) |  |
| indirect | -0.85 | (-2.0; | 0.22) |  |
| network | -0.39 | (-1.2; | 0.38) |  |
| CBASP vs. MBCT |  |  |  | **0.0098** |
| direct | -3.00 | (-6.5; | -0.70) |  |
| indirect | 0.14 | (-0.66; | 0.98) |  |
| network | -0.34 | (-1.2; | 0.49) |  |
| CBASP vs. MM |  |  |  | 0.4978 |
| direct | 0.34 | (-0.58; | 1.3) |  |
| indirect | 0.90 | (-0.49; | 2.2) |  |
| network | 0.50 | (-0.29; | 1.3) |  |
| CBASP vs. TAU |  |  |  | **0.0067** |
| direct | -3.00 | (-6.5; | -0.67) |  |
| indirect | 0.44 | (-0.47; | 1.4) |  |
| network | -0.15 | (-1.1; | 0.72) |  |
| CBASP vs. WL |  |  |  | 0.5428 |
| direct | -0.20 | (-2.2; | 1.8) |  |
| indirect | -0.88 | (-2.0; | 0.24) |  |
| network | -0.71 | (-1.7; | 0.23) |  |
| CT_CBT vs. MBCT |  |  |  | 0.1597 |
| direct | -1.10 | (-3.1; | 0.72) |  |
| indirect | 0.31 | (-0.54; | 1.2) |  |
| network | 0.05 | (-0.72; | 0.85) |  |
| CT_CBT vs. MCT |  |  |  | **0.0117** |
| direct | 0.04 | (-1.1; | 1.2) |  |
| indirect | -18.00 | (-60.; | -1.6) |  |
| network | -0.26 | (-1.4; | 0.84) |  |
| CT_CBT vs. MM |  |  |  | 0.37235 |
| direct | 1.50 | (-0.22; | 3.3) |  |
| indirect | 0.61 | (-0.38; | 1.7) |  |
| network | 0.89 | (0.026; | 1.8) |  |
| CT_CBT vs. PP |  |  |  | 0.2756 |
| direct | 0.16 | (-1.1; | 1.4) |  |
| indirect | -1.40 | (-4.1; | 1.1) |  |
| network | -0.13 | (-1.2; | 0.95) |  |
| CT_CBT vs. TAU |  |  |  | 0.74985 |
| direct | 0.49 | (-1.3; | 2.3) |  |
| indirect | 0.17 | (-0.78; | 1.1) |  |
| network | 0.24 | (-0.57; | 1.0) |  |
| CT_CBT vs. WL |  |  |  | 0.856 |
| direct | -0.46 | (-2.4; | 1.3) |  |
| indirect | -0.28 | (-1.3; | 0.75) |  |
| network | -0.31 | (-1.2; | 0.56) |  |
| DBT vs. TAU |  |  |  | 0.96405 |
| direct | 0.10 | (-1.3; | 1.5) |  |
| indirect | 0.17 | (-2.7; | 2.9) |  |
| network | 0.12 | (-1.1; | 1.4) |  |
| DBT vs. WL |  |  |  | 0.94715 |
| direct | -0.35 | (-3.1; | 2.2) |  |
| indirect | -0.45 | (-2.2; | 1.3) |  |
| network | -0.43 | (-1.8; | 0.97) |  |
| MBCT vs. MM |  |  |  | 0.3049 |
| direct | -0.20 | (-2.5; | 2.0) |  |
| indirect | 1.00 | (0.076; | 2.0) |  |
| network | 0.83 | (-0.048; | 1.7) |  |
| MBCT vs. TAU |  |  |  | 0.50545 |
| direct | -0.02 | (-1.2; | 1.2) |  |
| indirect | 0.49 | (-0.49; | 1.4) |  |
| network | 0.18 | (-0.62; | 0.95) |  |
| MBCT vs. WL |  |  |  | 0.23865 |
| direct | -0.86 | (-2.0; | 0.28) |  |
| indirect | 0.09 | (-1.0; | 1.2) |  |
| network | -0.37 | (-1.2; | 0.43) |  |
| MCT vs. WL |  |  |  | **0.01885** |
| direct | 12.00 | (0.64; | 39.) |  |
| indirect | -0.52 | (-2.0; | 0.95) |  |
| network | -0.05 | (-1.4; | 1.3) |  |
| PP vs. TAU |  |  |  | 0.2729 |
| direct | 1.50 | (-0.82; | 4.0) |  |
| indirect | -0.06 | (-1.6; | 1.4) |  |
| network | 0.37 | (-0.87; | 1.6) |  |

**Figure S23.** Network graph subgroup Treatment resistant and persistent depression

# Figure S24: Severity distribution per comparison

**Figure S24. Boxplot of mean baseline depression severity per comparison from 1 = minimal/mild to 4 = (very) severe**. Legend: ACT = Acceptance and commitment therapy, ActiveCt = Active control group, BA = Behavioral activation therapy, CBASP = Cognitive behavioral analysis system of psychotherapy, CT/CBT =Cognitive therapy/Cognitive behavioral therapy, DBT = Dialectical behavioral therapy, MBCT = Mindfulness-based cognitive therapy, MCT = Meta-cognitive therapy, MM= Medication management, PP = Positive psychotherapy, ST = Schema therapy, TAU = Treatment as usual, WL= Waitlist.

# Figure S25: Results of the Risk of Bias assessment

# Text S26: GRADE criteria (16)

(1) Study limitations: For individual studies, risk of bias ratings across all four domains were coded as low, some concern and major concern for risk of bias (15). The contributions of the individual studies’ ratings to the available d

irect comparisons are visualized in figure S10. Risk of bias across domains was summarized for individual studies as follows:

- Low risk of bias: if none of the domains had major concern for risks of bias. No downgrade.

- Some concern: If one domain was rated to be of major concern of risk of bias. Downgrade by -1.

- High risk: If two or more domains were of major concern for risk of bias. Downgrade by -2.

In a second step, using the study contributions matrix, the studies’ respective downgrading coefficients were multiplied with the studies’ contribution weight to the mixed or indirect effects, resulting in a weighted average risk of bias.

(2) Imprecision: We considered a standardized mean difference of 0.2 and -0.2 as clinically meaningful. All confidence intervals that covered null effects and both 0.2 and -0.2 were considered to be of major concern for imprecision since no inference can be made from the effect estimate if either treatment is more effective or if they are equally effective. Confidence intervals covering null effects and either 0.2 or -0.2 on one side were considered of some concern since it cannot be inferred whether the treatments are equally effective or less/more effective. Confidence intervals covering only 0.2 or -0.2 or lie in between these boundaries were of no concern for imprecision since clear inference can be drawn on the clinical importance of the effect estimate.

(3) Heterogeneity: Heterogeneity for acceptability data was below the 50% quantile of the empirical distribution for the heterogeneity variance for subjective outcomes of non-pharmacological interventions in the published literature (16, 17). However, heterogeneity was high for efficacy data I^2^ = 73%. Major concerns were present when the predictive interval included effect sizes (0.2 and -0.2) with different implications for practice. Some concerns were present when the prediction interval extended into both clinically important and unimportant effects. No concerns were present when confidence and prediction intervals agreed in relation to clinically important effects.

(4) Inconsistency: Inconsistency was judged based on node-splitting analyses. All loops that showed significant differences between direct and indirect estimates (p<0.05) for efficacy data were downgraded by one. We did not downgrade the same network estimate for both imprecision and inconsistency.

(5) Indirectness: in judging indirectness we consider if the face validity of the transitivity assumption holds. We included only studies that included patients formally diagnosed with unipolar major depressive disorder since inclusion of subthreshold symptomatology appears to overestimate effect sizes. We assessed effect modifiers such as baseline severity and in meta-regression and assessed treatment resistant and persistent courses in a subnetwork sensitivity analysis. We downgraded singly-connected nodes since their transitivity cannot be assessed in node-splitting analyses.

(6) Publication bias: Publication bias was assessed as effects of small studies in Egger’s regression test for each pairwise meta-analysis that had k>=3 studies. When no small study effects were found, we rated the comparison as being of no concern for publication bias and major concern if significant. All other comparisons were rated as some concern.


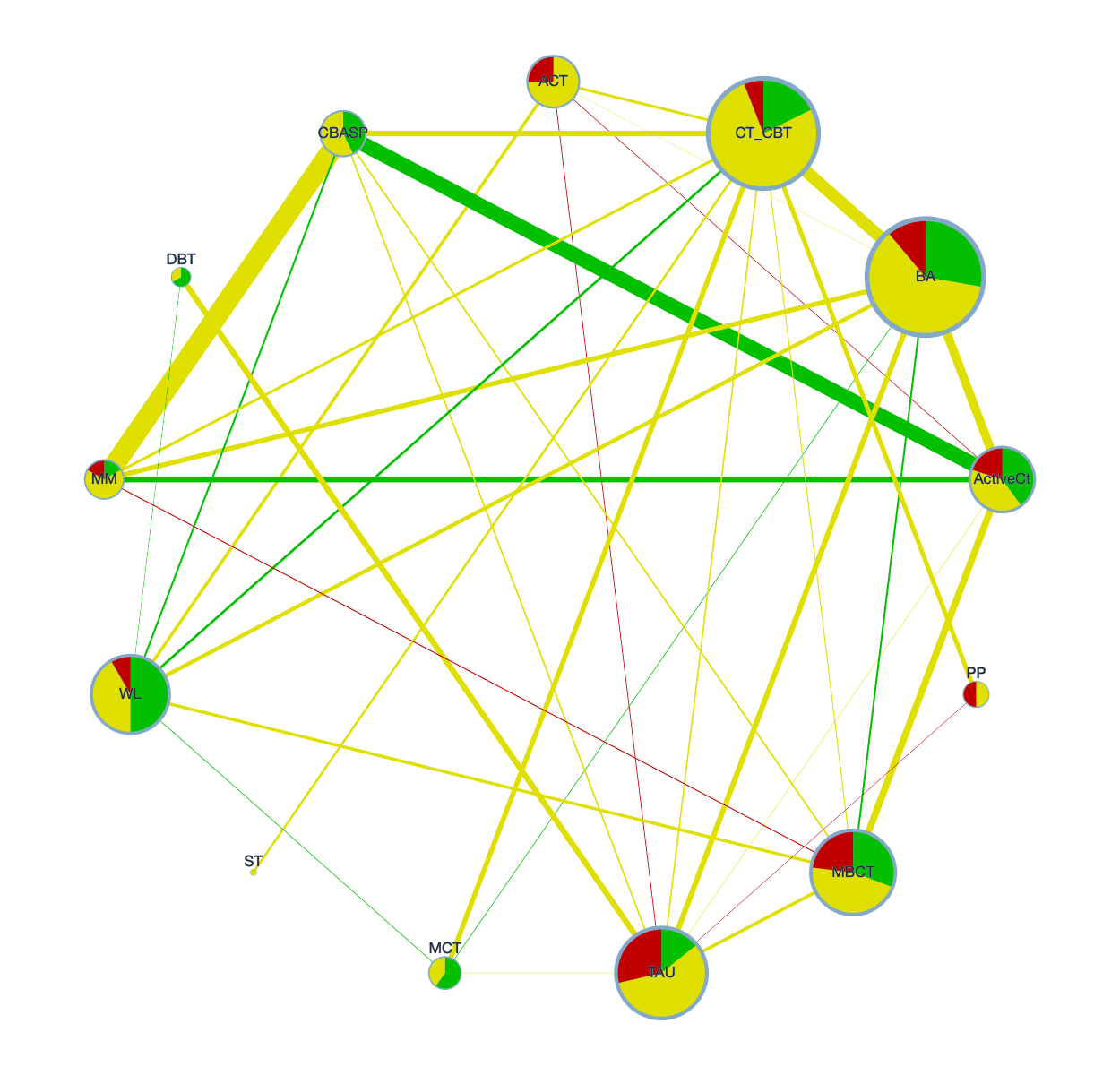


**Figure S27. Network graph displaying the distribution of the risk of bias within the network according to Cochrane’s RoB2.** Edge color corresponds to average risk of bias rating for the individual comparison. Edge size is proportionate to precision. Node size is proportionate to the number of studies and node color reflects the distribution of risk of bias in the individual node. Legend: Red: high risk of bias, yellow: some concerns, green: low risk of bias. ACT = Acceptance and commitment therapy, ActiveCt = Active control group, BA = Behavioral activation therapy, CBASP = Cognitive behavioral analysis system of psychotherapy, CT/CBT =Cognitive therapy/Cognitive behavioral therapy, DBT = Dialectical behavioral therapy, MBCT = Mindfulness-based cognitive therapy, MCT = Meta-cognitive therapy, MM= Medication management, PP = Positive psychotherapy, ST = Schema therapy, TAU = Treatment as usual, WL= Waitlist.

**Figure S28.** Bar graph showing the contribution of risk of bias ratings of the individual studies to the unique direct comparisons. Dashed line represents 30%.

## Table S29: Results of the GRADE rating

|  | Number of studies | Study limitations | Indirectness | Imprecision | Heterogeneity | Inconsistency | Publication Bias | Confidence rating |
| --- | --- | --- | --- | --- | --- | --- | --- | --- |
| Mixed estimates |  |  |  |  |  |  |  |  |
| ACT:ActiveCt | 1 | -1 | No concerns | No concerns | Some concerns | Major concerns | Some concerns | low |
| ACT:BA | 1 | -1 | No concerns | Major concerns | No concerns | No concerns | Some concerns | low |
| ACT:CT_CBT | 3 | -1 | No concerns | Major concerns | No concerns | No concerns | No concerns | low |
| ACT:TAU | 1 | -1 | No concerns | No concerns | No concerns | No concerns | Some concerns | moderate |
| ACT:WL | 2 | -1 | No concerns | No concerns | No concerns | Some concerns | Some concerns | moderate |
| ActiveCt:BA | 4 | -1 | No concerns | No concerns | Some concerns | No concerns | No concerns | moderate |
| ActiveCt:CBASP | 2 | 0 | No concerns | No concerns | Major concerns | No concerns | Some concerns | very low |
| ActiveCt:MBCT | 3 | -1 | No concerns | No concerns | Major concerns | No concerns | Some concerns | very low |
| ActiveCt:MM | 1 | -1 | No concerns | Some concerns | Some concerns | No concerns | Some concerns | low |
| ActiveCt:TAU | 1 | -1 | No concerns | Some concerns | Some concerns | No concerns | Some concerns | low |
| BA:CT_CBT | 4 | -1 | No concerns | Major concerns | No concerns | No concerns | No concerns | low |
| BA:MBCT | 1 | -1 | No concerns | Some concerns | Some concerns | No concerns | Some concerns | low |
| BA:MCT | 1 | 0 | No concerns | No concerns | Major concerns | No concerns | Some concerns | low |
| BA:MM | 2 | -1 | No concerns | Some concerns | Some concerns | No concerns | Some concerns | low |
| BA:TAU | 5 | -1 | No concerns | No concerns | No concerns | No concerns | No concerns | moderate |
| BA:WL | 4 | 0 | No concerns | No concerns | No concerns | No concerns | No concerns | moderate |
| CBASP:CT_CBT | 2 | -1 | No concerns | Some concerns | Some concerns | No concerns | Some concerns | low |
| CBASP:MBCT | 1 | -1 | No concerns | Major concerns | No concerns | No concerns | Some concerns | low |
| CBASP:MM | 3 | -1 | No concerns | Major concerns | No concerns | No concerns | Major concerns | very low |
| CBASP:TAU | 1 | -1 | No concerns | No concerns | Some concerns | No concerns | Some concerns | low |
| CBASP:WL | 1 | 0 | No concerns | No concerns | No concerns | No concerns | Some concerns | moderate |
| CT_CBT:MBCT | 1 | -1 | No concerns | Some concerns | Some concerns | No concerns | Some concerns | low |
| CT_CBT:MCT | 3 | -1 | No concerns | No concerns | Major concerns | Major concerns | No concerns | very low |
| CT_CBT:MM | 1 | -1 | No concerns | Some concerns | Some concerns | No concerns | Some concerns | low |
| CT_CBT:PP | 3 | -1 | No concerns | Major concerns | No concerns | No concerns | No concerns | low |
| CT_CBT:ST | 1 | -1 | Some concerns | Major concerns | No concerns | Major concerns | Some concerns | low |
| CT_CBT:TAU | 2 | -1 | No concerns | No concerns | No concerns | No concerns | Some concerns | moderate |
| CT_CBT:WL | 1 | 0 | No concerns | No concerns | No concerns | No concerns | Some concerns | moderate |
| DBT:TAU | 2 | 0 | No concerns | Some concerns | Some concerns | No concerns | Some concerns | low |
| DBT:WL | 1 | 0 | No concerns | Some concerns | Some concerns | No concerns | Some concerns | low |
| MBCT:MM | 1 | -1 | No concerns | Major concerns | No concerns | No concerns | Some concerns | low |
| MBCT:TAU | 4 | -1 | No concerns | No concerns | Some concerns | No concerns | Major concerns | low |
| MBCT:WL | 3 | -1 | No concerns | No concerns | No concerns | Some concerns | No concerns | low |
| MCT:TAU | 1 | -1 | No concerns | No concerns | No concerns | No concerns | Some concerns | low |
| MCT:WL | 2 | 0 | No concerns | No concerns | No concerns | No concerns | Some concerns | moderate |
| PP:TAU | 1 | -1 | No concerns | No concerns | No concerns | No concerns | Some concerns | moderate |
| **Indirect estimates** |  |  |  |  |  |  |  |  |
| ACT:CBASP | 0 | -1 | No concerns | Major concerns | No concerns | Some concerns | Some concerns | very low |
| ACT:DBT | 0 | -1 | No concerns | Some concerns | Some concerns | Some concerns | Some concerns | low |
| ACT:MBCT | 0 | -1 | No concerns | Some concerns | Some concerns | Some concerns | Some concerns | low |
| ACT:MCT | 0 | -1 | No concerns | Some concerns | Some concerns | Some concerns | Some concerns | low |
| ACT:MM | 0 | -1 | No concerns | Some concerns | Some concerns | Some concerns | Some concerns | low |
| ACT:PP | 0 | 0 | No concerns | Major concerns | No concerns | Some concerns | Some concerns | very low |
| ACT:ST | 0 | -1 | Some concerns | Major concerns | No concerns | Some concerns | Some concerns | very low |
| ActiveCt:CT_CBT | 0 | -1 | No concerns | No concerns | Some concerns | Some concerns | Some concerns | low |
| ActiveCt:DBT | 0 | -1 | No concerns | Major concerns | No concerns | Some concerns | Some concerns | very low |
| ActiveCt:MCT | 0 | -1 | No concerns | No concerns | No concerns | Some concerns | Some concerns | low |
| ActiveCt:PP | 0 | -1 | No concerns | No concerns | Some concerns | Some concerns | Some concerns | low |
| ActiveCt:ST | 0 | -1 | Some concerns | Major concerns | No concerns | Some concerns | Some concerns | very low |
| ActiveCt:WL | 0 | -1 | No concerns | No concerns | Major concerns | Some concerns | Some concerns | low |
| BA:CBASP | 0 | -1 | No concerns | Some concerns | Some concerns | Some concerns | Some concerns | low |
| BA:DBT | 0 | -1 | No concerns | Some concerns | Some concerns | Some concerns | Some concerns | low |
| BA:PP | 0 | -1 | No concerns | Major concerns | No concerns | Some concerns | Some concerns | very low |
| BA:ST | 0 | -1 | Some concerns | Major concerns | No concerns | Some concerns | Some concerns | very low |
| CBASP:DBT | 0 | -1 | No concerns | Major concerns | No concerns | Some concerns | Some concerns | very low |
| CBASP:MCT | 0 | -1 | No concerns | No concerns | Major concerns | Some concerns | Some concerns | very low |
| CBASP:PP | 0 | -1 | No concerns | Major concerns | No concerns | Some concerns | Some concerns | very low |
| CBASP:ST | 0 | -1 | Some concerns | Major concerns | No concerns | Some concerns | Some concerns | very low |
| CT_CBT:DBT | 0 | -1 | No concerns | Some concerns | Some concerns | Some concerns | Some concerns | low |
| DBT:MBCT | 0 | -1 | No concerns | Major concerns | No concerns | Some concerns | Some concerns | very low |
| DBT:MCT | 0 | 0 | No concerns | No concerns | Some concerns | Some concerns | Some concerns | low |
| DBT:MM | 0 | -1 | No concerns | Major concerns | No concerns | Some concerns | Some concerns | very low |
| DBT:PP | 0 | -1 | No concerns | Some concerns | Some concerns | Some concerns | Some concerns | low |
| DBT:ST | 0 | -1 | Some concerns | Major concerns | No concerns | Some concerns | Some concerns | very low |
| MBCT:MCT | 0 | -1 | No concerns | No concerns | Some concerns | Some concerns | Some concerns | low |
| MBCT:PP | 0 | -1 | No concerns | Some concerns | Some concerns | Some concerns | Some concerns | low |
| MBCT:ST | 0 | -1 | Some concerns | Major concerns | No concerns | Some concerns | Some concerns | very low |
| MCT:MM | 0 | -1 | No concerns | No concerns | Some concerns | Some concerns | Some concerns | low |
| MCT:PP | 0 | -1 | No concerns | Major concerns | No concerns | Some concerns | Some concerns | very low |
| MCT:ST | 0 | -1 | Some concerns | Major concerns | No concerns | Some concerns | Some concerns | very low |
| MM:PP | 0 | -1 | No concerns | Some concerns | Some concerns | Some concerns | Some concerns | low |
| MM:ST | 0 | -1 | Some concerns | Major concerns | No concerns | Some concerns | Some concerns | very low |
| MM:TAU | 0 | -1 | No concerns | No concerns | Major concerns | Some concerns | Some concerns | very low |
| MM:WL | 0 | -1 | No concerns | No concerns | Some concerns | Some concerns | Some concerns | low |
| PP:ST | 0 | -1 | Some concerns | Major concerns | No concerns | Some concerns | Some concerns | very low |
| PP:WL | 0 | -1 | No concerns | No concerns | No concerns | Some concerns | Some concerns | low |
| ST:TAU | 0 | -1 | Some concerns | Some concerns | Some concerns | Some concerns | Some concerns | low |
| ST:WL | 0 | -1 | Some concerns | No concerns | Some concerns | Some concerns | Some concerns | low |
| TAU:WL | 0 | -1 | No concerns | Major concerns | No concerns | Some concerns | Some concerns | very low |

# Table S30: Individual study data and effect sizes

|  | study | treatment | control | N intervention | Mean .intervention | SD intervention | N control | Mean control | SD control | SMD | 95% CrI for SMD | Dropouts intervention | N treated intervention | Droupouts control | N treated control | OR | 95% CrI for OR |
| --- | --- | --- | --- | --- | --- | --- | --- | --- | --- | --- | --- | --- | --- | --- | --- | --- | --- |
| 1 | A-Tjak 2018 | ACT | CT_CBT | 44 | 11.08 | 9.02 | 38 | 7.91 | 9.31 | 0.35 | [-0.09; 0.78] | 6 | 44 | 6 | 38 | 0.84 | [0.25; 2.87] |
| 2 | Arjadi 2018 | BA | ActiveCt | 159 | 8.5 | 5.75 | 154 | 10.83 | 6.21 | -0.39 | [-0.61; -0.17] | 39 | 159 | 9 | 154 | 5.24 | [2.44; 11.24] |
| 3 | Asgharipoor 2012 | PP | CT_CBT | 9 | 17.11 | 10.81 | 9 | 11.44 | 6.71 | 0.63 | [-0.32; 1.58] | NA | 9 | NA | 9 | NA | [NA; NA] |
| 4 | Ashouri 2013 | MCT | TAU | 10 | 16.1 | 2.51 | 13 | 25.55 | 3.55 | -3 | [-4.2; -1.81] | NA | 10 | NA | 13 | NA | [NA; NA] |
| 5 | Ashouri 2013 | MCT | CT_CBT | 10 | 16.1 | 2.51 | 10 | 18.3 | 4.62 | -0.59 | [-1.49; 0.3] | NA | 10 | NA | 10 | NA | [NA; NA] |
| 6 | Ashouri 2013 | CT_CBT | TAU | 10 | 18.3 | 4.62 | 13 | 25.55 | 3.55 | -1.79 | [-2.77; -0.82] | NA | 10 | NA | 10 | NA | [NA; NA] |
| 7 | Barnhofer 2009 | MBCT | TAU | 16 | 17.62 | 10.94 | 15 | 28.86 | 12.97 | -0.94 | [-1.68; -0.2] | 2 | 16 | 1 | 15 | 2 | [0.16; 24.66] |
| 8 | Bolinski 2018 | BA | CT_CBT | 20 | 20.2 | 10.6 | 23 | 18.3 | 11.6 | 0.17 | [-0.43; 0.77] | 1 | 20 | 0 | 23 | 3.62 | [0.14; 93.84] |
| 9 | Callesen 2020 | MCT | CT_CBT | 82 | 2.67 | 4.21 | 73 | 2.93 | 3.88 | -0.06 | [-0.38; 0.25] | 29 | 85 | 33 | 89 | 0.88 | [0.47; 1.64] |
| 10 | Carter 2013 | ST | CT_CBT | 50 | 12.9 | 12.3 | 50 | 11.4 | 9.5 | 0.14 | [-0.26; 0.53] | 11 | 50 | 14 | 50 | 0.73 | [0.29; 1.8] |
| 11 | Chaves 2017 | PP | CT_CBT | 47 | 23.43 | 12.39 | 49 | 22.42 | 14.01 | 0.08 | [-0.32; 0.48] | 10 | 47 | 10 | 49 | 1.05 | [0.39; 2.82] |
| 12 | Chiesa 2015 | MBCT | ActiveCt | 23 | 9.82 | 7.35 | 20 | 13.8 | 7.32 | -0.54 | [-1.15; 0.07] | 4 | 23 | 6 | 20 | 0.49 | [0.12; 2.08] |
| 13 | Cladder-Micus 2018 | MBCT | ActiveCt | 44 | 35.27 | 12.66 | 52 | 40.52 | 11.83 | -0.43 | [-0.84; -0.02] | 12 | 46 | 5 | 57 | 3.67 | [1.19; 11.35] |
| 14 | Cullen 2003 | BA | WL | 9 | 3.83 | 3.3 | 8 | 28.3 | 16.32 | -2.15 | [-3.34; -0.95] | 9 | 12 | 8 | 10 | 0.75 | [0.1; 5.69] |
| 15 | DeJong 2018 | MBCT | WL | 26 | 14.9 | 8.1 | 14 | 15.6 | 4.9 | -0.1 | [-0.75; 0.55] | 6 | 25 | 1 | 14 | 4.11 | [0.44; 38.23] |
| 16 | Dimidjian 2006 | BA | CT_CBT | 43 | 7.71 | 7.26 | 45 | 8.93 | 6.31 | -0.18 | [-0.6; 0.24] | 4 | 40 | 5 | 44 | 0.87 | [0.22; 3.48] |
| 17 | Dimidjian 2006 | BA | MM | 43 | 7.71 | 7.26 | 100 | 8.55 | 6.43 | -0.13 | [-0.48; 0.23] | 4 | 40 | 30 | 86 | 0.21 | [0.07; 0.64] |
| 18 | Dimidjian 2006 | CT_CBT | MM | 45 | 8.93 | 6.31 | 100 | 8.55 | 6.43 | 0.06 | [-0.29; 0.41] | 5 | 44 | 30 | 86 | 0.24 | [0.09; 0.67] |
| 19 | Eisendrath 2015 | MBCT | MM | 23 | 8.9 | 4.3 | 20 | 10.2 | 6.3 | -0.24 | [-0.85; 0.36] | 4 | 23 | 3 | 20 | 1.19 | [0.23; 6.11] |
| 20 | Eisendrath 2016 | MBCT | ActiveCt | 87 | 11.6 | 3.36 | 86 | 13 | 3.48 | -0.41 | [-0.71; -0.11] | 11 | 87 | 14 | 86 | 0.74 | [0.32; 1.75] |
| 21 | Ekers 2011 | BA | TAU | 23 | 11.93 | 11.84 | 24 | 27.4 | 14.01 | -1.19 | [-1.81; -0.57] | 6 | 22 | 1 | 23 | 8.25 | [0.9; 75.41] |
| 22 | Far 2017 | ACT | CT_CBT | 10 | 28.2 | 16.28 | 9 | 18.54 | 7.65 | 0.75 | [-0.19; 1.68] | NA | 10 | NA | 9 | NA | [NA; NA] |
| 23 | Fereidooni 2015 | BA | TAU | 8 | 16.25 | 2.81 | 8 | 17.37 | 2.5 | -0.42 | [-1.41; 0.57] | 0 | 8 | 0 | 8 | NA | [NA; NA] |
| 24 | Fereidooni 2015 | BA | ActiveCt | 8 | 16.25 | 2.81 | 8 | 17.37 | 2.97 | -0.39 | [-1.38; 0.6] | 0 | 8 | 0 | 8 | NA | [NA; NA] |
| 25 | Fereidooni 2015 | ActiveCt | TAU | 8 | 17.37 | 2.97 | 8 | 17.37 | 2.5 | 0 | [-0.98; 0.98] | 0 | 8 | 0 | 8 | NA | [NA; NA] |
| 26 | Folke 2012 | ACT | TAU | 18 | 15.43 | 9.61 | 16 | 22.45 | 11.13 | -0.68 | [-1.37; 0.01] | 4 | 18 | 6 | 16 | 0.48 | [0.11; 2.14] |
| 27 | Foroughi 2020 | MBCT | TAU | 10 | 5.11 | 4 | 9 | 17.11 | 3.6 | -3.14 | [-4.49; -1.8] | 5 | 15 | 6 | 15 | 0.75 | [0.17; 3.33] |
| 28 | Furchtlehner 2019 | PP | CT_CBT | 46 | 8.8 | 7.97 | 46 | 14.6 | 7.41 | -0.75 | [-1.18; -0.33] | 11 | 46 | 9 | 46 | 1.29 | [0.48; 3.49] |
| 29 | Hagen 2017 | MCT | WL | 20 | 6.4 | 6.84 | 19 | 23.89 | 7.05 | -2.52 | [-3.36; -1.68] | 0 | 20 | 2 | 19 | 0.17 | [0.01; 3.8] |
| 30 | Hamidian 2013 | MBCT | TAU | 22 | 17 | 12.2 | 22 | 24.2 | 8.7 | -0.68 | [-1.29; -0.07] | 3 | 25 | 3 | 25 | 1 | [0.18; 5.51] |
| 31 | Harley 2008 | DBT | WL | 10 | 11.3 | 5.31 | 9 | 17.11 | 6.23 | -1.01 | [-1.96; -0.05] | 3 | 13 | 2 | 11 | 1.35 | [0.18; 10.01] |

| 32 | Hemanny 2019 | BA | CT_CBT | 24 | 9.1 | 7.3 | 26 | 9.84 | 5.9 | -0.11 | [-0.67; 0.44] | 10 | 24 | 13 | 26 | 0.71 | [0.23; 2.18] |
| --- | --- | --- | --- | --- | --- | --- | --- | --- | --- | --- | --- | --- | --- | --- | --- | --- | --- |
| 33 | Hemanny 2019 | BA | TAU | 24 | 9.1 | 7.3 | 26 | 17.98 | 6.3 | -1.31 | [-1.92; -0.7] | 10 | 24 | 16 | 26 | 0.45 | [0.14; 1.39] |
| 34 | Hemanny 2019 | CT_CBT | TAU | 26 | 9.84 | 5.9 | 26 | 17.98 | 6.3 | -1.33 | [-1.93; -0.73] | 13 | 26 | 16 | 26 | 0.62 | [0.21; 1.88] |
| 35 | Hopko 2003 | BA | ActiveCt | 10 | 19.1 | 13.1 | 15 | 30.2 | 17 | -0.71 | [-1.54; 0.11] | 0 | 15 | 0 | 10 | NA | [NA; NA] |
| 36 | Jordan 2014 | MCT | CT_CBT | 23 | 8.1 | 6.6 | 25 | 7.5 | 5.9 | 0.1 | [-0.47; 0.66] | 6 | 23 | 5 | 25 | 1.41 | [0.37; 5.45] |
| 37 | Kanter 2015 | BA | ActiveCt | 21 | 11 | 9.14 | 22 | 12.83 | 9.7 | -0.19 | [-0.79; 0.41] | 5 | 19 | 9 | 20 | 0.44 | [0.11; 1.68] |
| 38 | Keller 2000 | CBASP | MM | 442 | 12.34 | 9.95 | 220 | 14.7 | 10.38 | -0.23 | [-0.4; -0.07] | 90 | 442 | 53 | 220 | 0.81 | [0.55; 1.19] |
| 39 | Kocsis 2009 | CBASP | MM | 200 | 11.29 | 8.3 | 96 | 12.28 | 8.44 | -0.12 | [-0.36; 0.12] | 25 | 200 | 16 | 96 | 0.71 | [0.36; 1.41] |
| 40 | Kocsis 2009 | CBASP | ActiveCt | 200 | 11.29 | 8.3 | 195 | 12.77 | 8.45 | -0.18 | [-0.37; 0.02] | 25 | 200 | 27 | 195 | 0.89 | [0.5; 1.59] |
| 41 | Kocsis 2009 | ActiveCt | MM | 195 | 12.77 | 8.45 | 96 | 12.28 | 8.44 | 0.06 | [-0.19; 0.3] | 96 | 195 | 16 | 27 | 0.67 | [0.29; 1.51] |
| 42 | Kyllonen 2018 | ACT | WL | 60 | 10.15 | 8.26 | 55 | 18.27 | 9.69 | -0.91 | [-1.29; -0.52] | 0 | 60 | 4 | 59 | 0.1 | [0.01; 1.94] |
| 43 | Lappalainen 2015 | ACT | WL | 18 | 13.34 | 6.75 | 20 | 17.85 | 7.34 | -0.64 | [-1.29; 0.01] | 1 | 19 | 0 | 20 | 3.32 | [0.13; 86.75] |
| 44 | Ledari 2018 | ACT | BA | 4 | 11 | 7.6 | 3 | 20.66 | 1.7 | -1.61 | [-3.33; 0.11] | 2 | 6 | 3 | 6 | 0.5 | [0.05; 5.15] |
| 45 | Lee 2021 | BA | TAU | 31 | 14.82 | 7.83 | 33 | 18.5 | 10.54 | -0.39 | [-0.89; 0.1] | 9 | 31 | 17 | 33 | 0.39 | [0.14; 1.08] |
| 46 | Ly 2014 | BA | MBCT | 40 | 10.89 | 5.92 | 41 | 12.94 | 10.18 | -0.25 | [-0.68; 0.19] | 6 | 40 | 5 | 41 | 1.27 | [0.35; 4.55] |
| 47 | Lynch 2003 | DBT | TAU | 15 | 6.9 | 8.67 | 16 | 7.77 | 4.61 | -0.13 | [-0.83; 0.58] | 1 | 17 | 1 | 17 | 1 | [0.06; 17.41] |
| 48 | Lynch 2018 | DBT | TAU | 162 | 15.97 | 8.8 | 88 | 19.74 | 7.22 | -0.46 | [-0.72; -0.19] | 24 | 147 | 16 | 88 | 0.88 | [0.44; 1.76] |
| 49 | Manicavasgar 2011 | MBCT | CT_CBT | 26 | 21.21 | 13.83 | 19 | 23.62 | 16.83 | -0.16 | [-0.75; 0.43] | 4 | 30 | 12 | 39 | 0.35 | [0.1; 1.21] |
| 50 | Michalak 2015 | CBASP | TAU | 35 | 14.64 | 8.85 | 35 | 21.16 | 8.16 | -0.77 | [-1.25; -0.28] | 10 | 35 | 1 | 35 | 13.6 | [1.63; 113.25] |
| 51 | Michalak 2015 | CBASP | MBCT | 35 | 14.64 | 8.85 | 36 | 17.86 | 10.37 | -0.33 | [-0.8; 0.13] | 10 | 35 | 1 | 35 | 13.6 | [1.63; 113.25] |
| 52 | Michalak 2015 | MBCT | TAU | 36 | 17.86 | 10.37 | 35 | 21.16 | 8.16 | -0.35 | [-0.82; 0.12] | 10 | 36 | 1 | 35 | 13.08 | [1.57; 108.74] |
| 53 | Moradveisi 2013 | BA | MM | 50 | 4.31 | 5.39 | 50 | 6.9 | 5.39 | -0.48 | [-0.88; -0.08] | 5 | 50 | 15 | 50 | 0.26 | [0.09; 0.78] |
| 54 | Nyström 2017 | BA | WL | 112 | 4.86 | 4.28 | 53 | 9.26 | 6.45 | -0.87 | [-1.21; -0.53] | 10 | 122 | 2 | 55 | 2.37 | [0.5; 11.18] |
| 55 | O'Mahen 2014 | BA | TAU | 41 | 11.05 | 4.71 | 42 | 14.26 | 5.11 | -0.65 | [-1.09; -0.21] | 4 | 41 | 8 | 42 | 0.46 | [0.13; 1.66] |
| 56 | Richards 2016 | BA | CT_CBT | 189 | 8.4 | 7 | 175 | 8.4 | 7.5 | 0 | [-0.21; 0.21] | 36 | 221 | 24 | 219 | 1.58 | [0.91; 2.75] |
| 57 | Rief 2018 | CBASP | CT_CBT | 43 | 17.68 | 10.63 | 88 | 14.58 | 11.1 | 0.28 | [-0.08; 0.65] | 6 | 43 | 15 | 88 | 0.79 | [0.28; 2.2] |
| 58 | Rief 2018 | CBASP | WL | 43 | 17.68 | 10.63 | 42 | 23.38 | 10.78 | -0.53 | [-0.97; -0.1] | 6 | 43 | 5 | 42 | 1.2 | [0.34; 4.28] |
| 59 | Rief 2018 | CT_CBT | WL | 88 | 14.58 | 11.1 | 42 | 23.38 | 10.78 | -0.8 | [-1.18; -0.42] | 15 | 88 | 5 | 42 | 1.52 | [0.51; 4.51] |
| 60 | Schramm 2015 | CBASP | MM | 29 | 21.97 | 9.64 | 30 | 18.75 | 10.63 | 0.32 | [-0.2; 0.83] | 2 | 29 | 4 | 30 | 0.48 | [0.08; 2.86] |
| 61 | Schramm 2017 | CBASP | ActiveCt | 137 | 14 | 9.72 | 131 | 16.49 | 9.96 | -0.25 | [-0.49; -0.01] | 12 | 135 | 14 | 125 | 0.77 | [0.34; 1.74] |
| 62 | Seligman 2006 | PP | TAU | 11 | 5.13 | 3.89 | 9 | 13 | 6.8 | -1.46 | [-2.45; -0.47] | 2 | 13 | 6 | 15 | 0.27 | [0.04; 1.69] |
| 63 | Shapero 2018 | MBCT | WL | 22 | 12.65 | 8.48 | 18 | 21 | 6.55 | -1.09 | [-1.75; -0.42] | 11 | 22 | 6 | 18 | 2 | [0.55; 7.25] |
| 64 | Soucy 2017 | BA | WL | 20 | 6.3 | 1.42 | 20 | 9.07 | 1.12 | -2.17 | [-2.95; -1.39] | 5 | 20 | 2 | 20 | 3 | [0.51; 17.74] |
| 65 | vanAalderen 2012 | MBCT | WL | 34 | 10.2 | 6.7 | 35 | 13.4 | 8.1 | -0.43 | [-0.91; 0.05] | 9 | 111 | 5 | 108 | 1.82 | [0.59; 5.61] |
| 66 | Wiersma 2014 | CBASP | CT_CBT | 67 | 24.1 | 14.1 | 72 | 32 | 14.5 | -0.55 | [-0.89; -0.21] | 14 | 67 | 15 | 72 | 1 | [0.44; 2.28] |
| 67 | Zemestani 2016 | BA | MCT | 15 | 15.38 | 2.25 | 15 | 15.78 | 2.51 | -0.17 | [-0.88; 0.55] | 0 | 15 | 0 | 15 | NA | [NA; NA] |
| 68 | Zemestani 2016 | MCT | WL | 15 | 15.78 | 2.51 | 15 | 29.21 | 2.42 | -5.45 | [-7; -3.89] | 0 | 15 | 0 | 15 | NA | [NA; NA] |
| 69 | Zemestani 2016 | BA | WL | 15 | 15.38 | 2.25 | 15 | 29.21 | 2.42 | -5.92 | [-7.58; -4.26] | 0 | 15 | 0 | 15 | NA | [NA; NA] |
| 70 | Zemestani 2020 | ACT | ActiveCt | 23 | 20.7 | 3.4 | 29 | 32.57 | 5.15 | -2.66 | [-3.41; -1.91] | 4 | 30 | 1 | 30 | 4.46 | [0.47; 42.51] |
| 71 | Zettle 2011 | ACT | CT_CBT | 12 | 11.27 | 7.39 | 13 | 16.2 | 10.83 | -0.53 | [-1.33; 0.27] | 1 | 12 | 3 | 13 | 0.3 | [0.03; 3.41] |

# References S31: References for the included studies

A-Tjak, J. G. L., Morina, N., Topper, M., & Emmelkamp, P. M. G. (2018). A Randomized Controlled Trial in Routine Clinical Practice Comparing Acceptance and Commitment Therapy with Cognitive Behavioral Therapy for the Treatment of Major Depressive Disorder. Psychother Psychosom, 87(3), 154-163.

Arjadi, R., Nauta, M., Scholte, W., Hollon, S., Chowdhary, N., Suryani, A., . . . Bockting, C. (2018). Internet-based behavioural activation with lay counsellor support versus online minimal psychoeducation without support for treatment of depression: a randomised controlled trial in Indonesia [Journal: Article]. The lancet psychiatry, 5(9), 707‐716.

Asgharipoor, N., Farid, A. A., Arshadi, H., & Sahebi, A. (2012). A comparative study on the effectiveness of positive psychotherapy and group cognitive-behavioral therapy for the patients suffering from major depressive disorder. Iranian Journal of Psychiatry and Behavioral Sciences, 6(2), 33‐41.

Ashouri, A., Atef Vahid, M. K., Gharaee, B., & Rasoulian, M. (2013). Effectiveness of meta-cognitive and cognitive-behavioral therapy in patients with major depressive disorder. Iran J Psychiatry Behav Sci, 7(2), 24-34.

Barnhofer, T., Crane, C., Hargus, E., Amarasinghe, M., Winder, R., & Williams, J. M. G. (2009). Mindfulness-based cognitive therapy as a treatment for chronic depression: A preliminary study. Behaviour Research and Therapy, 47(5), 366-373.

Bolinski, F., Hendriks, G. J., Bardoel, S., Hollon, S. D., Martell, C., & Huibers, M. J. H. (2018). Cognitive Therapy or Behavioral Activation for Major Depressive Disorder in Dutch Mental Health Care: Pilot Effectiveness and Process Trial. International Journal of Cognitive Therapy, 11(3), 343-358.

Callesen, P., Reeves, D., Heal, C., & Wells, A. (2020). Metacognitive Therapy versus Cognitive Behaviour Therapy in Adults with Major Depression: A Parallel Single-Blind Randomised Trial. Sci Rep, 10(1), 7878.

Carter, J. D., McIntosh, V. V., Jordan, J., Porter, R. J., Frampton, C. M., & Joyce, P. R. (2013). Psychotherapy for depression: a randomized clinical trial comparing schema therapy and cognitive behavior therapy. J Affect Disord, 151(2), 500-505.

Chaves, C., Lopez-Gomez, I., Hervas, G., & Vazquez, C. (2017). A comparative study on the efficacy of a positive psychology intervention and a cognitive behavioral therapy for clinical depression. Cognit Ther Res, 41(3), 417-433.

Chiesa, A., Castagner, V., Andrisano, C., Serretti, A., Mandelli, L., Porcelli, S., & Giommi, F. (2015). Mindfulness-based cognitive therapy vs. psycho-education for patients with major depression who did not achieve remission following antidepressant treatment. Psychiatry Res, 226(2-3), 474-483.

Cladder-Micus MB, Speckens AEM, Vrijsen JN, Donders ART, Becker ES, Spijker J. Mindfulness-based cognitive therapy for patients with chronic, treatment-resistant depression: A pragmatic randomized controlled trial. Depression and anxiety. 2018;35(10):914-24. (18) (18) (18) (18)

Cullen, J. M. (2002). Testing the effectiveness of behavioral activation therapy in the treatment of acute unipolar depression. Western Michigan University. Retrieved from https://www.proquest.com/docview/305500947?pq-origsite=gscholar&fromopenview=true (Accessed 22.09.2023)

de Jong, M., Peeters, F., Gard, T., Ashih, H., Doorley, J., Walker, R., . . . Mischoulon, D. (2018). A Randomized Controlled Pilot Study on Mindfulness-Based Cognitive Therapy for Unipolar Depression in Patients With Chronic Pain. J Clin Psychiatry, 78(1), 11328.

Dimidjian, S., Goodman, S. H., Sherwood, N. E., Simon, G. E., Ludman, E., Gallop, R., . . . Beck, A. (2017). A pragmatic randomized clinical trial of behavioral activation for depressed pregnant women. J Consult Clin Psychol, 85(1), 26-36.

Dimidjian, S., Hollon, S. D., Dobson, K. S., Schmaling, K. B., Kohlenberg, R. J., Addis, M. E., . . . Jacobson, N. S. (2006). Randomized trial of behavioral activation, cognitive therapy, and antidepressant medication in the acute treatment of adults with major depression. J Consult Clin Psychol, 74(4), 658-670.

Eisendrath, S. J., Gillung, E., Delucchi, K., Mathalon, D. H., Yang, T. T., Satre, D. D., . . . Wolkowitz, O. M. (2015). A Preliminary Study: Efficacy of Mindfulness-Based Cognitive Therapy versus Sertraline as First-line Treatments for Major Depressive Disorder. Mindfulness, 6(3), 475-482.

Eisendrath, S. J., Gillung, E., Delucchi, K. L., Segal, Z. V., Nelson, J. C., McInnes, L. A., . . . Feldman, M. D. (2016). A Randomized Controlled Trial of Mindfulness-Based Cognitive Therapy for Treatment-Resistant Depression. Psychother Psychosom, 85(2), 99-110.

Ekers, D., Richards, D., McMillan, D., Bland, J. M., & Gilbody, S. (2011). Behavioural activation delivered by the non-specialist: phase II randomised controlled trial. Br J Psychiatry, 198(1), 66-72.

Far, S. T., Gharraee, B., Birashk, B., & Habibi, M. (2017). Effectiveness of Acceptance and Commitment Therapy and Cognitive Therapy in Patients With Major Depressive Disorder. Iranian Journal of Psychiatry and Behavioral Sciences, 11(4), 10-14.

Fereidooni, S., Gharaei, B., Birashk, B., Sahraeian, A., & Hoseini, S. M. D. (2015). Efficacy of Group Behavioral Treatment as Supplemental Treatment to Pharmacotherapy in Inpatients with Depressive Disorders. Journal of Mood Disorders, 5(3), 104-112.

Folke, F., Parling, T., & Melin, L. (2012). Acceptance and Commitment Therapy for Depression: A Preliminary Randomized Clinical Trial for Unemployed on Long-Term Sick Leave. Cognitive and Behavioral Practice, 19(4), 583-594.

Foroughi, A., Sadeghi, K., Parvizifard, A., Parsa Moghadam, A., Davarinejad, O., Farnia, V., & Azar, G. (2020). The effectiveness of mindfulness-based cognitive therapy for reducing rumination and improving mindfulness and self-compassion in patients with treatment-resistant depression. Trends Psychiatry Psychother, 42(2), 138-146.

Furchtlehner, L. M., Schuster, R., & Laireiter, A.-R. (2020). A comparative study of the efficacy of group positive psychotherapy and group cognitive behavioral therapy in the treatment of depressive disorders: A randomized controlled trial. The Journal of Positive Psychology, 15(6), 832-845.

Hagen, R., Hjemdal, O., Solem, S., Kennair, L. E. O., Nordahl, H. M., Fisher, P., & Wells, A. (2017). Metacognitive Therapy for Depression in Adults: A Waiting List Randomized Controlled Trial with Six Months Follow-Up. Frontiers in Psychology, 8, 31.

Hamidian, S., Omidi, A., Mousavinasab, S. M., & Naziri, G. (2013). Comparison of the Effect of Mindfulness-based Cognitive Therapy Accompanied by Pharmacotherapy With Pharmacotherapy Alone in Treating Dysthymic Patients. Iran Red Crescent Med J, 15(3), 239-244.

Harley, R., Sprich, S., Safren, S., Jacobo, M., & Fava, M. (2008). Adaptation of dialectical behavior therapy skills training group for treatment-resistant depression. J Nerv Ment Dis, 196(2), 136-143.

Hemanny C, Carvalho C, Maia N, Reis D, Botelho AC, Bonavides D, et al. Efficacy of trial-based cognitive therapy, behavioral activation and treatment as usual in the treatment of major depressive disorder: preliminary findings from a randomized clinical trial. CNS Spectr. 2020;25(4):535-44.

Hopko, D. R., Lejuez, C. W., LePage, J. P., Hopko, S. D., & McNeil, D. W. (2003). A brief behavioral activation treatment for depression - A randomized pilot trial within an inpatient psychiatric hospital. Behavior Modification, 27(4), 458-469.

Jordan, J., Carter, J. D., McIntosh, V. V. W., Fernando, K., Frampton, C. M. A., Porter, R. J., . . . Joyce, P. R. (2014). Metacognitive therapy versus cognitive behavioural therapy for depression: a randomized pilot study. Australian and New Zealand Journal of Psychiatry, 48(10), 932-943.

Kanter, J. W., Santiago-Rivera, A. L., Santos, M. M., Nagy, G., Lopez, M., Hurtado, G. D., & West, P. (2015 A randomized hybrid efficacy and effectiveness trial of behavioral activation for Latinos with depression. Behavior Therapy, 46(2), 177-192.

Keller, M. B., McCullough, J. P., Klein, D. N., Arnow, B., Dunner, D. L., Gelenberg, A. J., . . . Rothbaum, B. (2000). A comparison of nefazodone, the cognitive behavioral-analysis system of psychotherapy, and their combination for the treatment of chronic depression. New England Journal of Medicine, 342(20), 1462-1470.

Kocsis, J. H., Gelenberg, A. J., Rothbaum, B. O., Klein, D. N., Trivedi, M. H., Manber, R., . . . Thase, M. E. (2009). Cognitive Behavioral Analysis System of Psychotherapy and Brief Supportive Psychotherapy for Augmentation of Antidepressant Nonresponse in Chronic Depression. Archives of General Psychiatry, 66(11), 1178-1188.

Kyllonen, H. M., Muotka, J., Puolakanaho, A., Astikainen, P., Keinonen, K., & Lappalainen, R. (2018). A brief Acceptance and Commitment Therapy intervention for depression: A randomized controlled trial with 3-year follow-up for the intervention group. Journal of Contextual Behavioral Science, 10, 55-63.

Lappalainen, P., Langrial, S., Oinas-Kukkonen, H., Tolvanen, A., & Lappalainen, R. (2015). Web-based acceptance and commitment therapy for depressive symptoms with minimal support: a randomized controlled trial. Behav Modif, 39(6), 805-834.

Ledari, R. B., Masjedi, A., Bakhtyari, M., Zarghami, M., Nouri, R., & Hosseini, H. (2018). A Comparison Between the Effectiveness of Acceptance and Commitment Treatment and Behavioral Activation Treatment for Depression on Symptoms Severity and Rumination Among Patients with Treatment-Resistant Depression. Iranian Journal of Psychiatry and Behavioral Sciences, 12(3).

Lee, E., Han, Y., Cha, Y. J., Oh, J.-H., Hwang, N.-R., Seo, H.-J., & Choi, K.-H. (2021). Community-Based Multi-Site Randomized Controlled Trial of Behavioral Activation for Patients with Depressive Disorders. Community Mental Health Journal, 1-13.

Ly, K., Trüschel, A., Jarl, L., Magnusson, S., Windahl, T., Johansson, R., . . . Andersson, G. (2014). Behavioural activation versus mindfulness-based guided self-help treatment administered through a smartphone application: a randomised controlled trial [Comparative Study; Randomized Controlled Trial; Research Support, Non‐U.S. Gov't]. BMJ Open, 4(1), e003440.

Lynch, T. R., Hempel, R. J., Whalley, B., Byford, S., Chamba, R., Clarke, P., . . . Remington, B. (2018). Radically open dialectical behaviour therapy for refractory depression: the RefraMED RCT.

Lynch, T. R., Morse, J. Q., Mendelson, T., & Robins, C. J. (2003). Dialectical behavior therapy for depressed older adults: a randomized pilot study. Am J Geriatr Psychiatry, 11(1), 33-45.

Manicavasgar, V., Parker, G., & Perich, T. (2011). Mindfulness-based cognitive therapy vs cognitive behaviour therapy as a treatment for non-melancholic depression. J Affect Disord, 130(1-2), 138-144.

Michalak, J., Schultze, M., Heidenreich, T., & Schramm, E. (2015). A Randomized Controlled Trial on the Efficacy of Mindfulness-Based Cognitive Therapy and a Group Version of Cognitive Behavioral Analysis System of Psychotherapy for Chronically Depressed Patients. Journal of consulting and clinical psychology, 83(5), 951-963.

Moradveisi, L., Huibers, M., Renner, F., Arasteh, M., & Arntz, A. (2013). Behavioural activation v. antidepressant medication for treating depression in Iran: randomised trial [Randomized Controlled Trial]. British Journal of Psychiatry, 202(3), 204‐211.

Nyström, M., Stenling, A., Sjöström, E., Neely, G., Lindner, P., Hassmén, P., . . . Carlbring, P. (2017). Behavioral activation versus physical activity via the internet: a randomized controlled trial [Randomized Controlled Trial]. Journal of Affective Disorders, 215, 85‐93.

O'Mahen, H. A., Richards, D. A., Woodford, J., Wilkinson, E., McGinley, J., Taylor, R. S., & Warren, F. C. (2014). Netmums: a phase II randomized controlled trial of a guided Internet behavioural activation treatment for postpartum depression. *Psychol Med, 44*(8), 1675-1689.

Richards, D. A., Ekers, D., McMillan, D., Taylor, R. S., Byford, S., Warren, F. C., . . . Finning, K. (2016). Cost and Outcome of Behavioural Activation versus Cognitive Behavioural Therapy for Depression (COBRA): a randomised, controlled, non-inferiority trial. *Lancet, 388*(10047), 871-880.

Rief, W., Bleichhardt, G., Dannehl, K., Euteneuer, F., & Wambach, K. (2018). Comparing the Efficacy of CBASP with Two Versions of CBT for Depression in a Routine Care Center: A Randomized Clinical Trial. Psychother Psychosom, 87(3), 164-178.

Schramm, E., Kriston, L., Zobel, I., Bailer, J., Wambach, K., Backenstrass, M., . . . Harter, M. (2017). Effect of Disorder-Specific vs Nonspecific Psychotherapy for Chronic Depression: A Randomized Clinical Trial. JAMA Psychiatry, 74(3), 233-242.

Schramm, E., Zobel, I., Schoepf, D., Fangmeier, T., Schnell, K., Walter, H., . . . Normann, C. (2015). Cognitive Behavioral Analysis System of Psychotherapy versus Escitalopram in Chronic Major Depression. Psychother Psychosom, 84(4), 227-240.

Seligman, M. E. P., Rashid, T., & Parks, A. C. (2006). Positive psychotherapy. Am Psychol, 61(8), 774-788.

Shapero, B. G., Greenberg, J., Mischoulon, D., Pedrelli, P., Meade, K., & Lazar, S. W. (2018). Mindfulness-Based Cognitive Therapy Improves Cognitive Functioning and Flexibility Among Individuals with Elevated Depressive Symptoms. Mindfulness (N Y), 9(5), 1457-1469.

Soucy, I., Provencher, M., Fortier, M., & McFadden, T. (2017). Efficacy of guided self-help behavioural activation and physical activity for depression: a randomized controlled trial. Cognitive Behaviour Therapy, 46(6), 493-506.

van Aalderen, J. R., Donders, A. R. T., Giommi, F., Spinhoven, P., Barendregt, H. P., & Speckens, A. E. M. (2012). The efficacy of mindfulness-based cognitive therapy in recurrent depressed patients with and without a current depressive episode: a randomized controlled trial. Psychological Medicine, 42(5), 989-1001.

Wiersma, J. E., Van Schaik, D. J. F., Hoogendorn, A. W., Dekker, J. J., Van, H. L., Schoevers, R. A., . . . Van Oppen, P. (2014). The Effectiveness of the Cognitive Behavioral Analysis System of Psychotherapy for Chronic Depression: A Randomized Controlled Trial. Psychother Psychosom, 83(5), 263-269.

Zemestani, M., Davoodi, I., Honarmand, M. M., Zargar, Y., & Ottaviani, C. (2016). Comparative effects of group metacognitive therapy versus behavioural activation in moderately depressed students. J Ment Health, 25(6), 479-485.

Zemestani, M., & Mozaffari, S. (2020). Acceptance and commitment therapy for the treatment of depression in persons with physical disability: a randomized controlled trial. Clin Rehabil, 34(7), 938-947.

Zettle, R. D., Rains, J. C., & Hayes, S. C. (2011). Processes of change in acceptance and commitment therapy and cognitive therapy for depression: a mediation reanalysis of Zettle and Rains. Behav Modif, 35(3), 265-283.

# References

1. Shapero B. G., Greenberg J., Mischoulon D., Pedrelli P., Meade K., Lazar S. W. (2018). Mindfulness-Based Cognitive Therapy Improves Cognitive Functioning and Flexibility among Individuals with Elevated Depressive Symptoms. *Mindfulness* 9(5),1457-69. <https://10.1007/s12671-018-0889-0>.

2. Jordan J., Carter J. D., McIntosh V. V. W., Fernando K., Frampton C. M. A., Porter R. J., et al. (2014). Metacognitive Therapy Versus Cognitive Behavioural Therapy for Depression: A Randomized Pilot Study. *Australian and New Zealand Journal of Psychiatry* 48(10),932-43. <https://10.1177/0004867414533015>.

3. Beck A. T., Steer R. A., Ball R., Ranieri W. (1996). Comparison of Beck Depression Inventories -Ia and -Ii in Psychiatric Outpatients. *J Pers Assess* 67(3),588-97. <https://10.1207/s15327752jpa6703_13>.

4. Radloff L. S. (1977). The Ces-D Scale: A Self-Report Depression Scale for Research in the General Population. *Applied psychological measurement* 1(3),385-401.

5. Fong T. C., Chan C. L., Ho R. T., Chan J. S., Chan C. H., Ng S. M. (2016). Dimensionality of the Center for Epidemiologic Studies Depression Scale: An Exploratory Bi-Factor Analytic Study. *Qual Life Res* 25(3),731-7. <https://10.1007/s11136-015-1105-5>.

6. Cox J. L., Holden J. M., Sagovsky R. (1987). Detection of Postnatal Depression. Development of the 10-Item Edinburgh Postnatal Depression Scale. *Br J Psychiatry* 150,782-6. <https://10.1192/bjp.150.6.782>.

7. Hamilton M. The Hamilton Rating Scale for Depression. *Assessment of Depression*. Berlin, Heidelberg: Springer (1986). p. 143-52.

8. Rush A. J., Giles D. E., Schlesser M. A., Fulton C. L., Weissenburger J., Burns C. (1986). The Inventory for Depressive Symptomatology (Ids): Preliminary Findings. *Psychiatry Res* 18(1),65-87. <https://10.1016/0165-1781(86)90060-0>.

9. Montgomery S. A., Asberg M. (1979). A New Depression Scale Designed to Be Sensitive to Change. *Br J Psychiatry* 134,382-9. <https://10.1192/bjp.134.4.382>.

10. Muller M. J., Himmerich H., Kienzle B., Szegedi A. (2003). Differentiating Moderate and Severe Depression Using the Montgomery-Asberg Depression Rating Scale (Madrs). *J Affect Disord* 77(3),255-60. <https://10.1016/s0165-0327(02)00120-9>.

11. Mittmann N., Mitter S., Borden E. K., Herrmann N., Naranjo C. A., Shear N. H. (1997). Montgomery-Asberg Severity Gradations. *Am J Psychiatry* 154(9),1320-1. <https://10.1176/ajp.154.9.1320b>.

12. Kroenke K., Spitzer R. L., Williams J. B. (2001). The Phq-9: Validity of a Brief Depression Severity Measure. *J Gen Intern Med* 16(9),606-13. <https://10.1046/j.1525-1497.2001.016009606.x>.

13. Rush A. J., Trivedi M. H., Ibrahim H. M., Carmody T. J., Arnow B., Klein D. N., et al. (2003). The 16-Item Quick Inventory of Depressive Symptomatology (Qids), Clinician Rating (Qids-C), and Self-Report (Qids-Sr): A Psychometric Evaluation in Patients with Chronic Major Depression. *Biological psychiatry* 54(5),573-83. <https://10.1016/s0006-3223(02)01866-8>.

14. van Valkenhoef G., Lu G., de Brock B., Hillege H., Ades A. E., Welton N. J. (2012). Automating Network Meta-Analysis. *Res Synth Methods* 3(4),285-99. <https://10.1002/jrsm.1054>.

15. Sterne J. A. C., Savovic J., Page M. J., Elbers R. G., Blencowe N. S., Boutron I., et al. (2019). Rob 2: A Revised Tool for Assessing Risk of Bias in Randomised Trials. *BMJ (Clinical research ed)* 366,l4898. <https://10.1136/bmj.l4898>.

16. Salanti G., Del Giovane C., Chaimani A., Caldwell D. M., Higgins J. P. (2014). Evaluating the Quality of Evidence from a Network Meta-Analysis. *PLoS One* 9(7),e99682. <https://10.1371/journal.pone.0099682>.

17. Turner R. M., Davey J., Clarke M. J., Thompson S. G., Higgins J. P. (2012). Predicting the Extent of Heterogeneity in Meta-Analysis, Using Empirical Data from the Cochrane Database of Systematic Reviews. *Int J Epidemiol* 41(3),818-27. <https://10.1093/ije/dys041>.

18. Cladder-Micus M. B., Speckens A. E. M., Vrijsen J. N., Donders A. R. T., Becker E. S., Spijker J. (2018). Mindfulness-Based Cognitive Therapy for Patients with Chronic, Treatment-Resistant Depression: A Pragmatic Randomized Controlled Trial. *Depression and Anxiety* 35(10),914-24. <https://10.1002/da.22788>.

1. mean baseline score > 20 rated as “high level of depressive symptoms” in the original publication (12). [↑](#footnote-ref-1)
